# A large Megaraptoridae (Theropoda: Coelurosauria) from Upper Cretaceous (Maastrichtian) of Patagonia, Argentina

**DOI:** 10.1038/s41598-022-09272-z

**Published:** 2022-04-26

**Authors:** Alexis M. Aranciaga Rolando, Matias J. Motta, Federico L. Agnolín, Makoto Manabe, Takanobu Tsuihiji, Fernando E. Novas

**Affiliations:** 1 Laboratorio de Anatomía Comparada y Evolución de los Vertebrados, Museo Argentino de Ciencias Naturales “Bernardino Rivadavia” (CONICET), Av. Ángel Gallardo 470, C1405DJR Ciudad Autónoma de Buenos Aires, Argentina; 2 Fundación de Historia Natural “Félix de Azara”, Departamento de Ciencias Naturales y Antropología, CEBBAD - Universidad Maimónides, Hidalgo 767, C1405BDB Buenos Aires, Argentina; 3 National Museum of Nature & Science, 4-1-1 Amakubo, Tsukuba, 305-0005 Japan

**Keywords:** Palaeontology, Evolution

## Abstract

Megaraptora is a theropod clade known from former Gondwana landmasses and Asia. Most members of the clade are known from the Early to Late Cretaceous (Barremian–Santonian), with Maastrichtian megaraptorans known only from isolated and poorly informative remains. The aim of the present contribution is to describe a partial skeleton of a megaraptorid from Maastrichtian beds in Santa Cruz Province, Argentina. This new specimen is the most informative megaraptoran known from Maastrichtian age, and is herein described as a new taxon. Phylogenetic analysis nested the new taxon together with other South American megaraptorans in a monophyletic clade, whereas Australian and Asian members constitute successive stem groups. South American forms differ from more basal megaraptorans in several anatomical features and in being much larger and more robustly built.

## Introduction

Megaraptorans are a group of predatory dinosaurs that inhabited Asia, Australia and South America from Barremian through Maastrichtian times^1–6^. These theropods are diagnosed by their elongate skulls, the presence of apicobasally short and strongly curved teeth that are 8-shaped in cross section, highly pneumatic axial skeleton reaching to the mid-caudal vertebrae, and long and powerful arms bearing large and sharp manual claws on digits I and II^1,2,6–14^.

Although some authors^1,15,16^ have interpreted megaraptorans as an archaic group of allosauroid theropods, increasing evidence lends support to the hypothesis that they are, instead, members of Coelurosauria^2,4,10,12,17–20^. Aside from the consensus currently arrived on the phylogenetic allocation of Megaraptora among Coelurosauria, the internal relations of this clade remain poorly resolved.

The record of Megaraptora in Asia corresponds to Upper Barremian sediments of Sao Khua Formation in Thailand (*Phuwiangvenator yaemniyomi* and *Vayuraptor nongbualamphuensis*^4,5^) and from Upper Barremian Kitadani Formation in Japan (*Fukuiraptor kitadaniensis*^21–23^). In Australia, the oldest records are from Barremian–Aptian rocks of the Strzelecki Group^24^, followed by those of the Albian Eumeralla Formation (*cf*. *Australovenator wintonensis*^24,25^). The Griman Creek Formation (Cenomanian^26^) also yielded megaraptorid remains (‘*Rapator ornitholestoides*’, and two indeterminate megaraptorids^27–29^). The youngest records of Megaraptoridae in Australia are from the Winton Formation (Cenomanian–?Turonian) as represented by *Australovenator wintonensis* and an indeterminate megaraptoran^7–9,27^.

In South America, the oldest record of a probable megaraptoran comes from the Albian Santana Formation in Brazil^30^. An isolated caudal centrum of an indeterminate megaraptorid was also reported from the Upper Cretaceous of Mato Grosso, Brazil^31^.

Argentine Patagonia has the richest megaraptorid record of the continent. The oldest Patagonian records consist of an indeterminate megaraptorid coming from Bajo Barreal Formation (Cenomanian-Turonian^20^) and *Aoniraptor libertatem* found in beds of the Huincul Formation (Cenomanian-Turonian^13,32^). The following records are, from oldest to youngest, *Megaraptor namunhuaiquii* from the Portezuelo Formation (Coniacian^6,10,17^), *Murusraptor barrosaensis* from the Sierra Barrosa Formation (Coniacian^33^), *Aerosteon riocoloradensis* from the Anacleto Formation (Santonian^34^), *Tratayenia rosalesi* from the Bajo de la Carpa Formation (Santonian^19^), *Orkoraptor burkei* from the Cerro Fortaleza Formation (Campanian^35^), and some indeterminate megaraptoran remains from two Maastrichtian units: Lago Colhué Huapi^36,37^ and Chorrillo formations^3^.

The aim of the present contribution is to describe a new species of large-bodied megaraptorid from lower Maastrichtian Chorrillo Formation in southwestern Santa Cruz Province, Argentina, which constitutes one of the youngest records for the entire group. The new materials enable the thoracic anatomy of megaraptorids to be thoroughly examined, and facilitate a review of some aspects of the evolutionary radiation of this group of carnivorous dinosaurs.

## Systematic palaeontology

- Theropoda Marsh, 1881.
- Tetanurae Gauthier, 1986.
- Coelurosauria von Huene, 1920.
- Megaraptora Benson et al., 2010.
- Megaraptoridae Novas et al., 2013.
- *Maip macrothorax* gen. et sp. nov.

Zoo bank registration: urn:lsid:zoobank.org:pub:E447F58E-28D6-4CFD-B5E3-BC4009DE5423.

### Etymology

**Figure 1:**
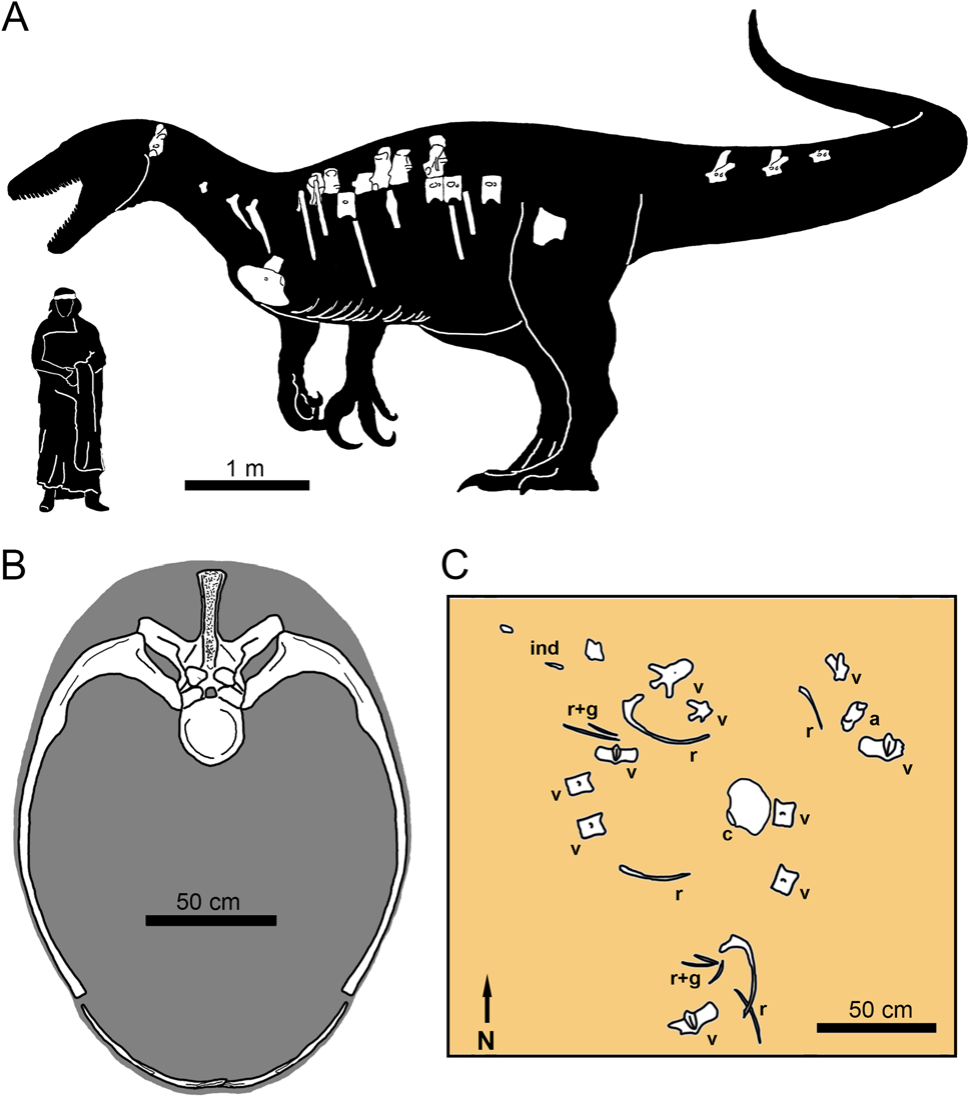
(**A**), silhouette of *Maip macrothorax* showing the preserved bones in white. (**B**), reconstruction of the thoracic cavity of *Maip *at level of D6. (**C**), interpretative drawing of the excavation of *Maip* showing the original disposition of the bones. Abbreviations: a, axis; c, coracoid; ind, indeterminate bone; g, gastralia; r, rib; v, vertebrae.

*Maip* (Fig. 1A) is an evil entity from the Aonikenk mythology that represents “the shadow of the death” which “kills with cold wind”, and roams in the Andes mountains. The specific name, *macro*, derives from the Latin “big” and “thorax” refers to its wide thoracic cavity (which has, approximately, more than 1.20 m width) (Fig. 1B).

### Holotype

MPM 21,545, including axis, dorsals 2, 3, 4, 5, 6, 7, 9, 10 (or 11), and 12 (or 13), two proximal caudals (caudal A, probably pertaining to caudal 3–5 and caudal B, pertaining to caudal 6–8), some fragmentary vertebrae, three incomplete cervical ribs, numerous incomplete or fragmentary dorsal ribs, numerous gastral elements, left coracoid, distal end of a second metatarsal, and fragments of the scapula. The holotype also includes the megaraptorid elements described by Novas et al*.*^3^, including the centrum of dorsal vertebra 12 or 13, a proximal caudal, a proximal right pubis, and the distal end of a second metatarsal (Fig. 1A). The specimen was found associated, but not in articulation, in a 5 × 3 m surface area, in a bed no more than 1 m thick (Fig. 1C).

### Locality

Megaraptorid Site (Locality 3; see Novas et al*.*^3^), La Anita Farm, 30 km SW of El Calafate city, Santa Cruz Province, Argentina. Chorrillo Formation (Maastrichtian^3,38,39^).

### Diagnosis

Megaraptorid theropod diagnosable on the basis of the following combination of characters (autapomorphies marked by an asterisk): (1) mid-dorsal vertebrae with articular surface of parapophyses saddle-shaped*; (2) mid-caudal vertebrae with an accessory PCDL that subdivides the PO-CDF in two pneumatic fossae*; (3) first dorsal rib with honeycomb internal structure on its tuberculum; (4) prominent anterior projection on the coracoid*; (5) coracoid without a subglenoid ridge *; (6) coracoid without a posteroventral fossa; and (7) and coracoid with ventromedial margin forming a dorsoventrally deep articular surface for the sternum*.

## Description

Some bones of holotype of *Maip macrothorax* (D12 or 13) have been previously described by Novas et al.^3^, thus we refer readers to this paper for descriptions and figures of those elements. Novas et al.^3^ described an isolated dorsal centrum of *Maip*, which is interpreted here as D12 or 13 based on a combination of characters (large size, strong transverse constriction, notably transversely wide anterior articular surface) observed in D13 of *Aerosteon*^14^.

### Axial skeleton

**Figure 2:**
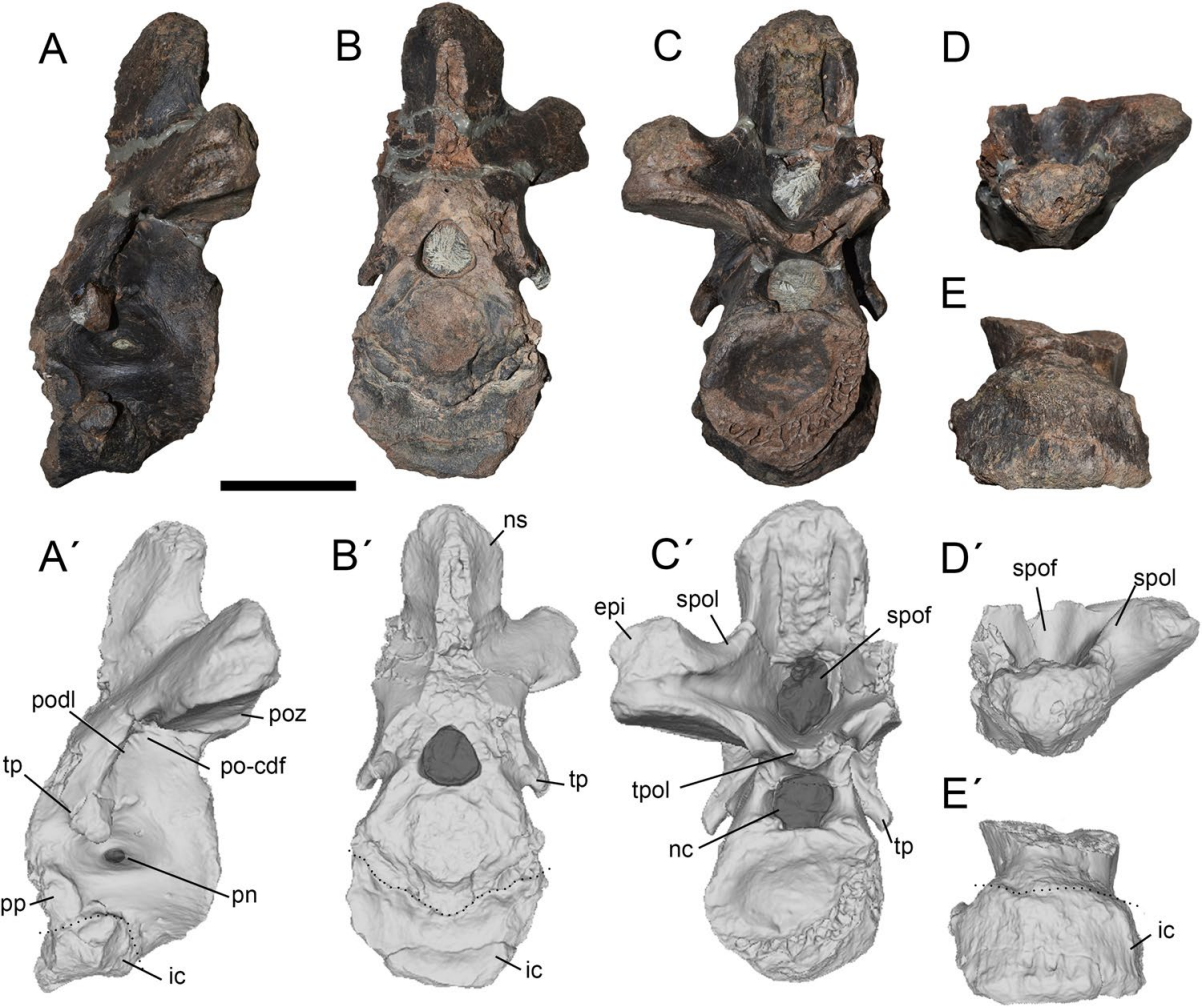
Axis of *Maip* in lateral (**A**, **A´**), anterior (**B**, **B´**), posterior (**C**, **C´**), dorsal (**D**, **D´**) and ventral (**E**, **E´**). Scale bar: 5 cm. Abbreviations: epi, epipophyses; ic, intercentrum; nc, neural canal; ns, neural spine; pn, pneumatopore; podl; postzygapophyseal lamina; po-cdf; postzygapophyseal-centrodiapophyseal fossa; poz, postzygapophyses; pp, parapophyses; prz, prezygapophyses; spof; spinopostzygapophyseal fossa; spol; spinopostzygapophyseal lamina; tp, transverse process; tpol, intrapostzygapophyseal lamina.

**Figure 3:**
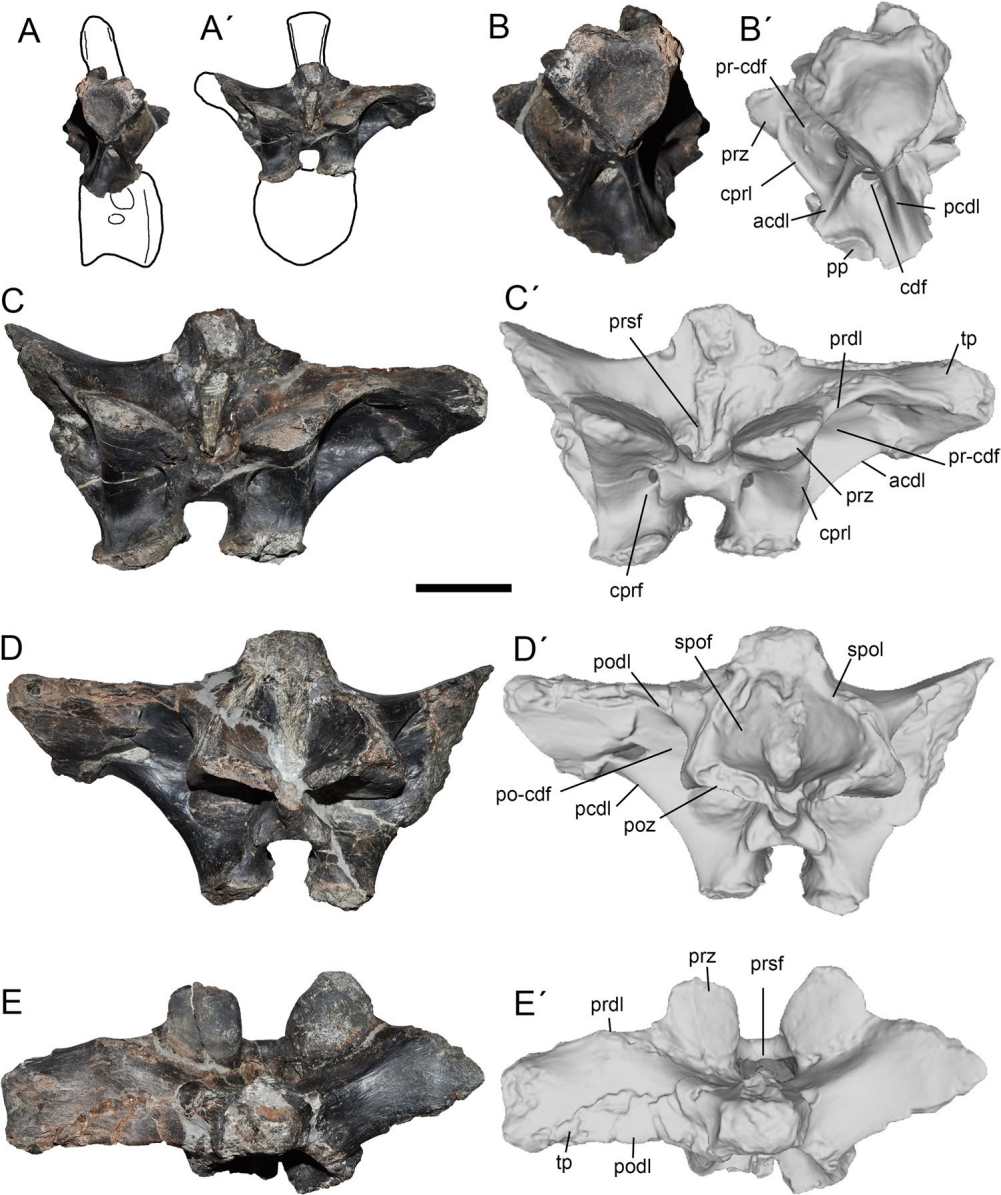
Second dorsal of *Maip*. Interpretative drawing showing the preserved parts of the bone (**A**, **A´**) and lateral (**B**, **B´**), anterior (**C**, **C´**), posterior (**D**, **D´**) and dorsal (**E**, **E´**) views. Scale bar: 5 cm. Abbreviations: acdl; anterior centrodiapophyseal lamina; cdf; centrodiapophyseal fossa; cprf; centroprezygapophyseal fossa; cprl; centroprezygapophyseal lamina; pcdl; posterior centrodiapophyseal lamina; podl; postzygapophyseal lamina; poz, postzygapophyses; po-cdf; postzygapophyseal-centrodiapophyseal fossa; pp, parapophyses; prdl; prezygodiapophyseal lamina; prsf; prespinal fossa; prz, prezygapophyses; pr-cdf; prezygapophyseal-centrodiapophyseal fossa; spof; spinopostzygapophyseal fossa; spol; spinopostzygapophyseal lamina; tp, transverse process.

**Figure 4:**
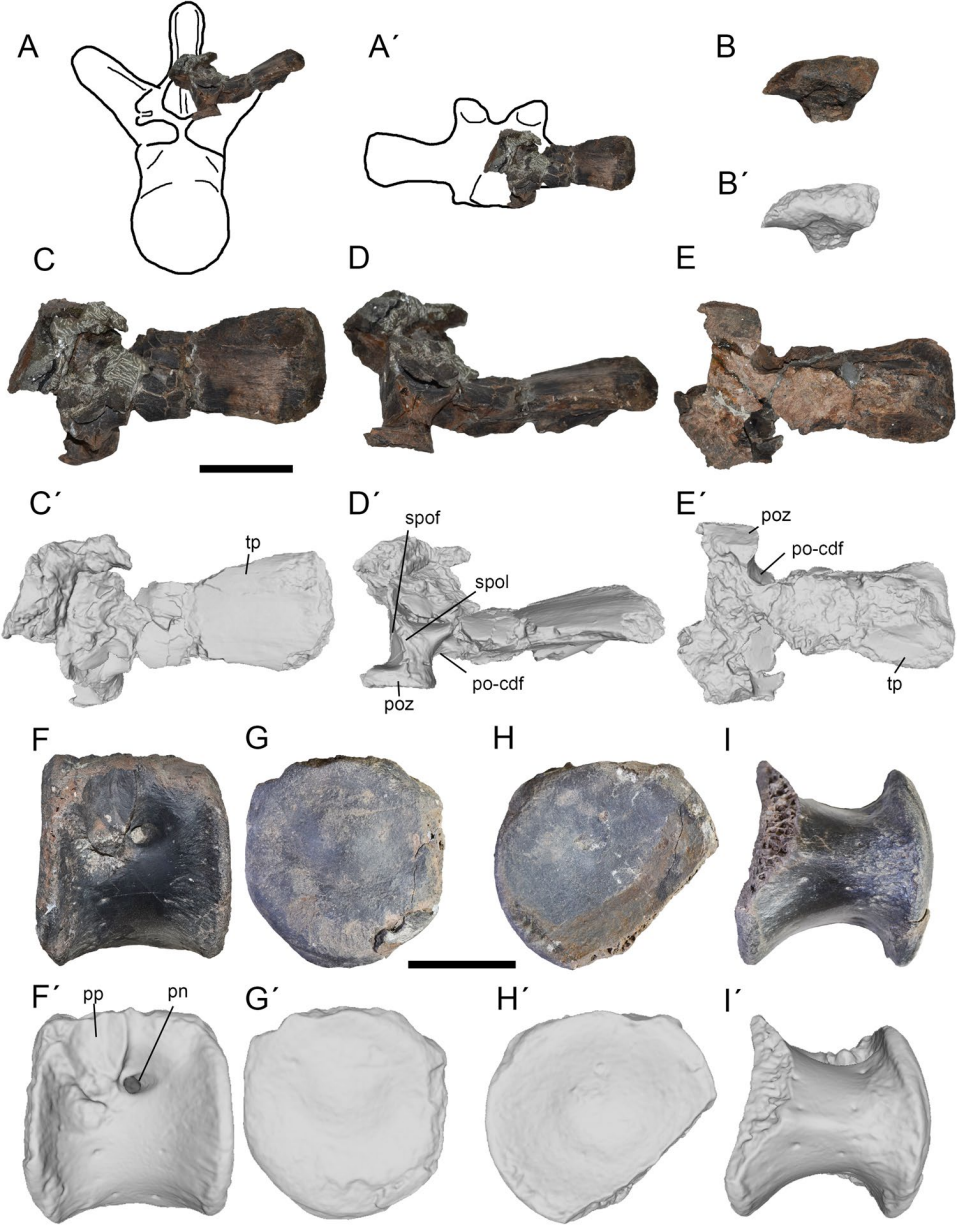
Third and fourth dorsals of *Maip*. Third dorsal, interpretative drawing showing the preserved parts (**A**, **A´**), lateral (**B**, **B´**), dorsal (**C**, **C´**), posterior (**D**, **D´**) and ventral (**E**, **E´**) views. Fourth dorsal in lateral (**B**, **B´**), anterior (**C**, **C´**), posterior (**D**, **D´**) and dorsal (**E**, **E´**) views. Scale bar: 5 cm. Abbreviations: pn, pneumatopore; poz, postzygapophyses; po-cdf; postzygapophyseal-centrodiapophyseal fossa; pp, parapophyses; spof; spinopostzygapophyseal fossa; spol; spinopostzygapophyseal lamina; tp, transverse process.

**Figure 5:**
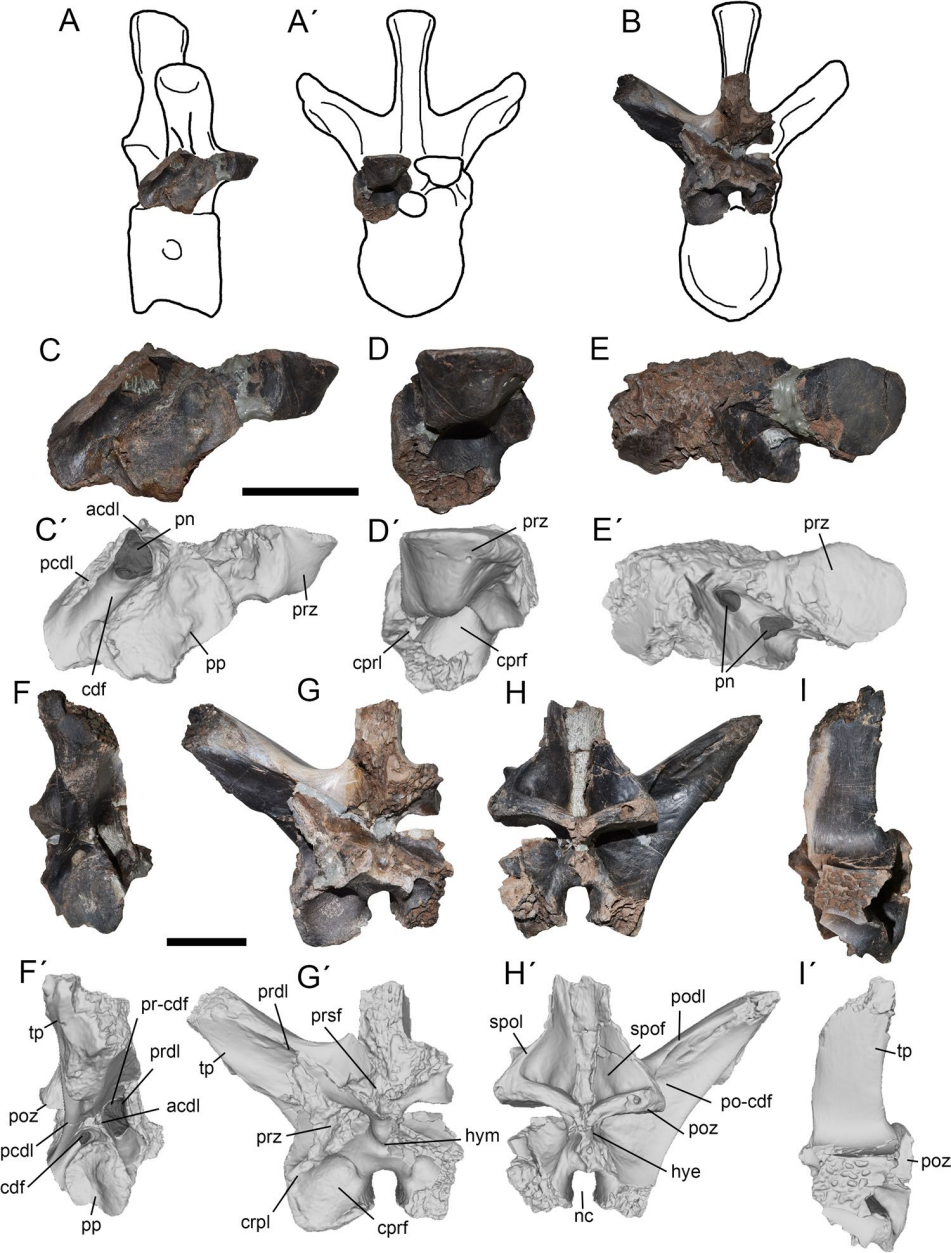
Fifth and sixth dorsals of *Maip*. Fifth dorsal, interpretative drawing showing the preserved parts (**A**, **A´**), lateral (**C**, **C´**), anterior (**D**, **D´**) and dorsal (**E**, **E´**) views. Sixth dorsal, interpretative drawing showing the preserved parts (**B**), lateral (**F**, **F´**), anterior (**G**, **G´**), posterior (**H**, **H´**) and dorsal (**I**, **I´**) views. Scale bar: 5 cm. Abbreviations: acdl; anterior centrodiapophyseal lamina; cdf; centrodiapophyseal fossa; cprf; centroprezygapophyseal fossa; cprl; centroprezygapophyseal lamina; hye, hyposphene; hym, hypantrum; nc, neural canal; pcdl; posterior centrodiapophyseal lamina; pn, pneumatopore; podl; postzygapophyseal lamina; poz, postzygapophyses; po-cdf; postzygapophyseal-centrodiapophyseal fossa; prdl; prezygodiapophyseal lamina; prsf; prespinal fossa; pp, parapophyses; prz, prezygapophyses; spof; spinopostzygapophyseal fossa; spol; spinopostzygapophyseal lamina; tp, transverse process.

**Figure 6:**
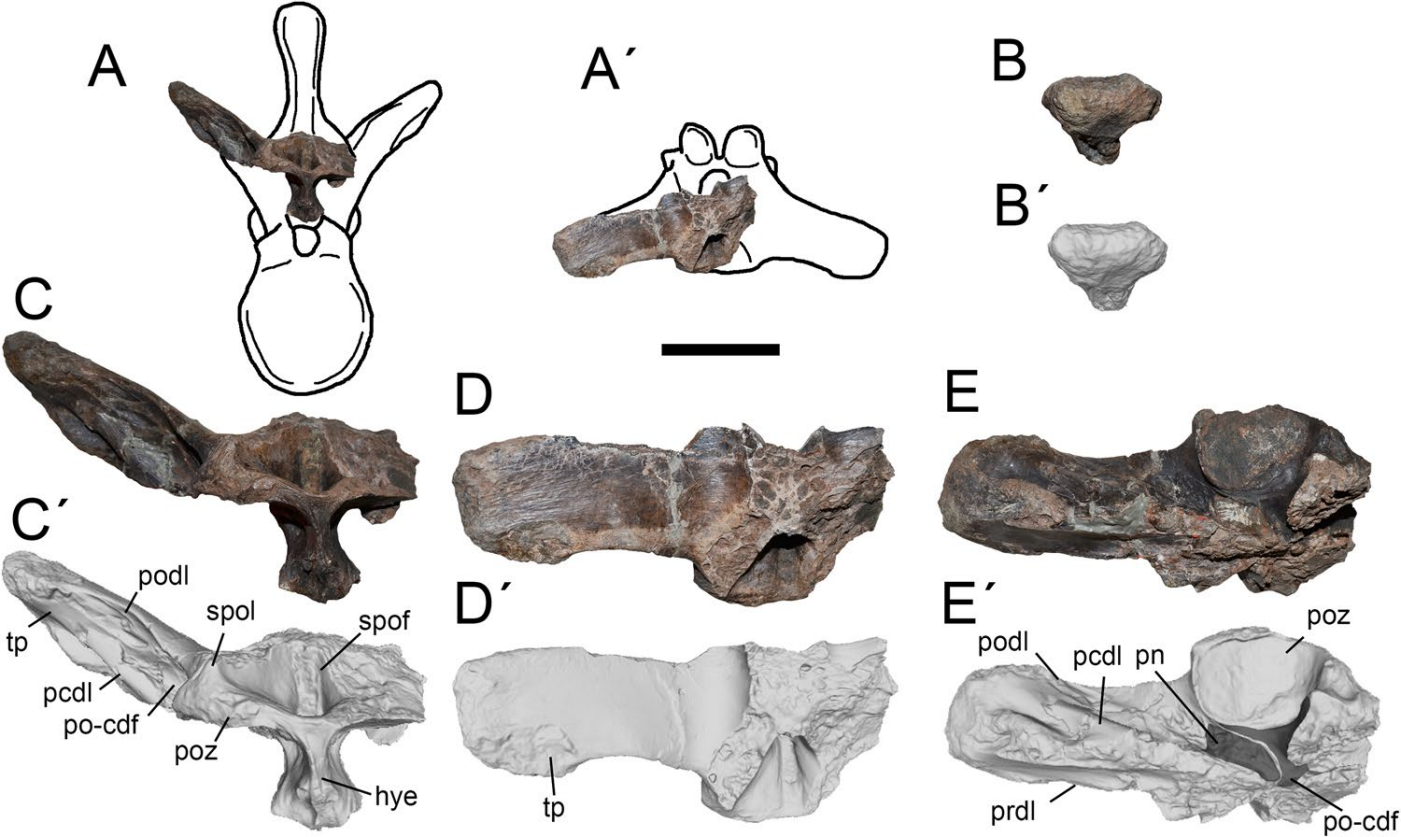
Seventh dorsal of *Maip*. Interpretative drawing showing the preserved parts (**A**, **A´**), lateral (**B**, **B´**), posterior (**C**, **C´**), dorsal (**D**, **D´**) and ventral (**E**, **E´**) views. Scale bar: 5 cm. Abbreviations: hym, hypantrum; pn, pneumatopore; pcdl; posterior centrodiapophyseal lamina; podl; postzygapophyseal lamina; poz, postzygapophyses; po-cdf; postzygapophyseal-centrodiapophyseal fossa; prdl; prezygodiapophyseal lamina; spof; spinopostzygapophyseal fossa; spol; spinopostzygapophyseal lamina; tp, transverse process.

**Figure 7:**
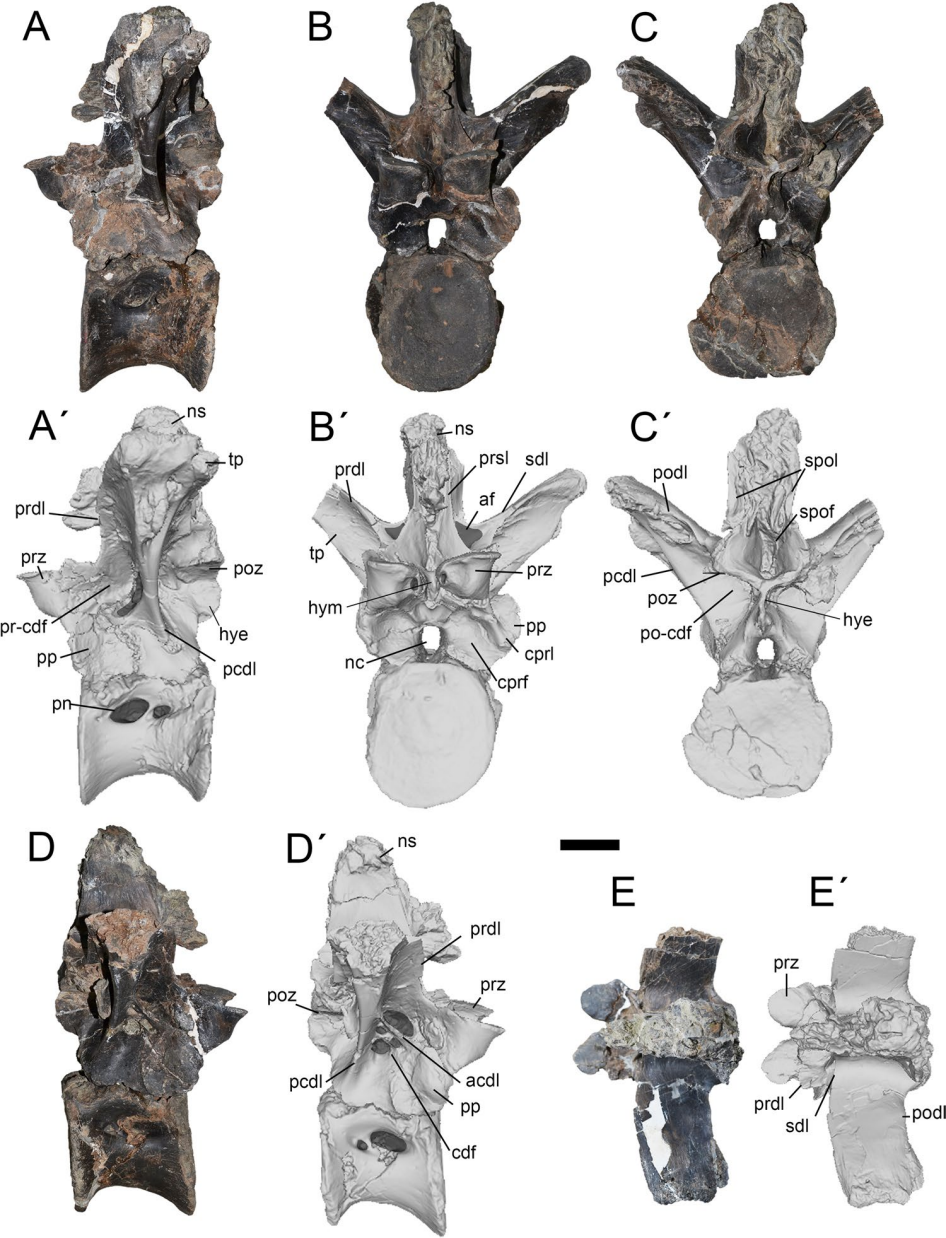
Ninth dorsal of *Maip*. Left lateral (**A**, **A´**), anterior (**B, B´**), posterior (**C, C´**), right lateral (**D, D´**) and dorsal (**E, E´**) views. Scale bar: 5 cm. Abbreviations: acdl; anterior centrodiapophyseal lamina; af, accessory fossa; cdf; centrodiapophyseal fossa; cprf; centroprezygapophyseal fossa; cprl; centroprezygapophyseal lamina; hye, hyposphene; hym, hypantrum; nc, neural canal; ns, neural spine; pcdl; posterior centrodiapophyseal lamina; pn, pneumatopore; podl; postzygapophyseal lamina; poz, postzygapophyses; po-cdf; postzygapophyseal-centrodiapophyseal fossa; prdl; prezygodiapophyseal lamina; prsf; prespinal fossa; pp, parapophyses; prz, prezygapophyses; pr-cdf; prezygapophyseal-centrodiapophyseal fossa; sdl; spinodiapophyseal lamina; spof; spinopostzygapophyseal fossa; spol; spinopostzygapophyseal lamina; tp, transverse process.

**Figure 8:**
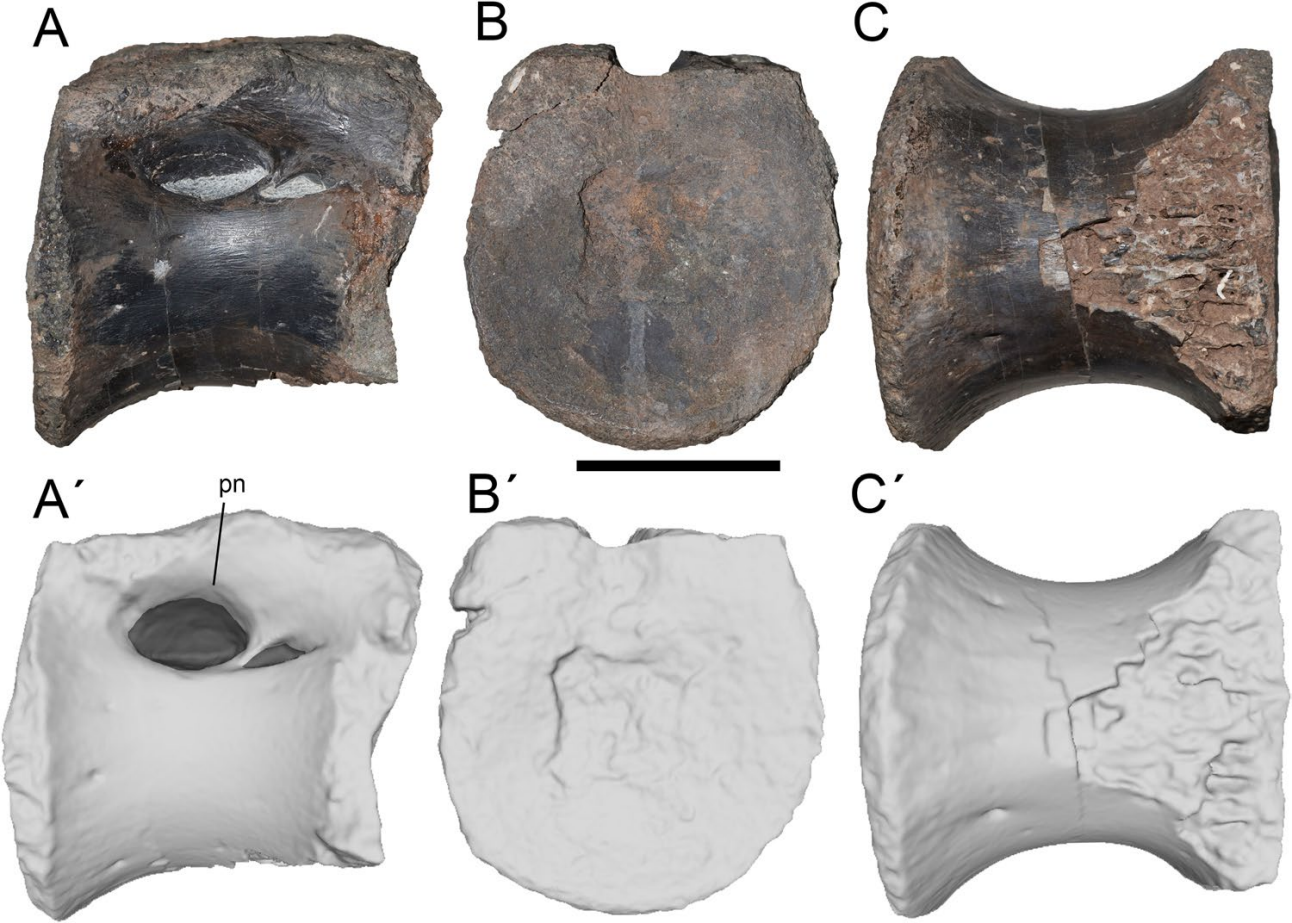
Tenth or eleventh dorsal of *Maip*. Lateral (**A, A´**), anterior (**B, B´**) and ventral (**C, C´**) views. Scale bar: 5 cm. Abbreviations: pn, pneumatopore.

Based on comparisons with the complete cervico-dorsal sequence preserved in a juvenile specimen of *Megaraptor namunhuaiquii*^10^, and holotypes of *Aerosteon riocoloradensis*^14,34^, *Murusraptor barrosaensis*^12,33^, and *Tratayenia rosalesi*^19^, the cervico-dorsal series of *Maip macrothorax* is represented by the axis (Fig. 2), dorsal vertebrae 2–7, 9, 10 or 11, and 12–13 (Figs. 3, 4, 5, 6, 7, 8). All vertebrae are camellated, as observed in many vertebrae because of the presence of broken areas exposing the internal structure of the bone. Only three caudal vertebrae are currently known for *Maip macrothorax*, and all are from the proximal third of the tail, thereby enabling comparison with the proximal caudals of *Megaraptor namunhuaiquii*^10^, *Orkoraptor burkei*^35^, and *Aerosteon riocoloradensis*^14,34^. The holotype of *Maip macrothorax* also preserves several ribs tentatively referred as cervical ribs 5, 7 and 8 (Fig. 10), and dorsal ribs 1, 2, and 6, as well as many other indeterminate rib fragments (Fig. 11). Many lateral and medial gastral elements have been recovered, probably belonging to the anterior half of the chest (Fig. 12).

#### Axis

As observed in other megaraptorids, *Maip macrothorax* exhibits proportionately dorsoventrally low axis as compared with posterior dorsal vertebrae, which are twice the height of the anterior cervical vertebrae. The axis is almost complete (Fig. 2), only lacking both prezygapophyses and its right postzygapophysis. In lateral view (Fig. 2A), it is taller than long, with the neural arch almost two times the height of the centrum. The neural spine is low but robust, being less than half the height of the neural arch. The intercentrum exhibits a rugose texture; it is placed ventral (not anterior) to the pleurocentrum, and immediately ventral to the parapophyses. The intercentrum is subtriangular in lateral view, and shows a moderate dorsolateral projection. Anteriorly, the intercentrum is crescent-shaped and anteriorly concave; it is subtriangular shaped in contour in ventral view. The pleurocentrum shows an irregular anterior surface, the odontoid process being damaged. The pleurocentrum is squared-shaped in lateral view, being slightly taller than long. It is laterally concave and shows an anteroposteriorly long, deep oval depression, perforated by a single pneumatopore on each side of the centrum. The centrum is rounded and moderately posteriorly concave. The posterior articular surface is wider than its anterior counterpart. The parapophyses are small, oval in contour (dorsoventrally taller than anteroposteriorly long), slightly concave and placed on the anterior margin of centrum.

The neurocentral suture is irregular. The transverse process is short, ventrolaterally oriented and close to the anterior margin of the vertebra. A moderately developed, obliquely oriented PODL is observed. This crest delimits a PO-CDF which exhibits a single pneumatic opening on its center. The postzygapophyses are concave and anteroposteriorly long in lateral view; in posterior aspect each is notably mediolaterally wide, with its articular surface ventrally concave and strongly laterally projected. The postzygapophyses bear oval (wider than long) articular surfaces. The TPOL is stout and dorsally concave. Posteriorly, the neural arch shows a deep and anteroposteriorly long SPOF. This pneumatic fossa is deep and subtriangular-shaped. The SPOL are stout, dorsally concave and reach the tip of the neural spine. The SPOL diverges posteriorly.

The epipophyses are stout, dorsoventrally low, and mediolaterally wide. They are subtriangular in side view, and posteriorly do not surpass the posterior level of centrum. In anterior view, the neural canal is subtriangular, but it is oval and notably wider than tall in posterior view. The latter is dorsoventrally short but transversely wide. The neural spine is square-shaped and low in side aspect. The anterior margin of the neural spine is broken, but it is evident that it is much thinner than the posterior margin, conferring a subtriangular cross-section to the neural spine. The intervertebral ligament rugosity is weak.

### Dorsal vertebrae

The transverse process of all dorsal neural arch (except the D9) is upturned (30° from the horizontal axis), mainly oriented perpendicular to the sagittal axis and, in dorsal view, the anterior and posterior margins of the transverse process are subparallel resulting in a subrectangular process. All vertebrae shows well-developed laminae and fossae. Below the transverse process, all vertebrae shows the same structures (but with different shape, sizes, and orientations). The PRDL (anteriorly) and the PODL (posteriorly) connects the transverse process with the pre- and postzygapophyses, respectively. Below the transverse process, are observed the ACDL and PCDL (in the D9 the ACDL is substantially reduced). These four mentioned laminae, delimits three fossae: the PR-CDF (anterior), the CDF (middle and below) and the PO-CDF (posterior). Almost all vertebrae, shows pneumatopores invading the bone on these fossae. Anteriorly, the neural archs shows the CPRL which delimits medioventrally the CPRF. The neural spine has, on its anterior side, the PRSL and, posteriorly, the SPOL. The former delimits the PRSF and the SPOL delimits the SPOF.

#### Dorsal 2.

It is represented by an incomplete neural arch (Fig. 3). The neural spine and the right transverse process are only preserved at its base.

This element is referred to D2 because of the presence of a strong and laterally facing PR-CDF, prominent CPRL, parapophyses placed partially on the centrum and transverse process anterior oriented, as also occurs in D2 of *Megaraptor namunhuaiquii*^10^ and *Murusraptor barrosaensis*^12,33^.

The neurocentral suture is ventrally convex. Only the roof of the parapophysis is placed in the neural arch indicating its anterior position on the column. The articular surface of the parapophysis is concave. In lateral view, the left transverse process is sub-horizontally oriented because of breakage, whereas the left process is dorsally oriented, which seems to be its original morphology. In dorsal view, the transverse process is anteroposteriorly long, and slightly posteriorly oriented. On its posterior surface, this process shows a deep and oval pneumatic opening located at its mid-length. The PR-CDF is subtriangular and pneumatic in nature. The CDF is subtriangular in shape and ventrally facing. This fossa is asymmetric, being pneumatic on the left side and apneumatic on the right. The PCDL is stout and obliquely oriented. The PO-CDF is deep and probably pneumatic. The transverse process shows a big, round, concave and laterally (or slightly ventrolaterally) facing diapophysis. The postzygapophyses face posteroventrally and are placed slightly below the prezygapophyses. In ventral view, the articular surface of the postzygapophyses is oval (wider than long) in contour and flat or slightly concave. More dorsally, the SPOLs are notably thick and stout. The SPOF is subrectangular in shape and exhibits subtle intervertebral ligament tuberosity. Furthermore, the SPOF is deep and shows a step-like convexity on its lateroventral corner. This feature is observed in other anterior dorsals of *Maip* but absent in more posterior elements. The prezygapophyses are strongly anterior- or anterodorsally facing. Its articular surfaces are round, flat and slightly medially inclined. The PRDL is sharp, sub-horizontally oriented and ventrally concave. In anterior view, the PRSF is deep and strongly pneumatic. This fossa shows a wide pneumatic recess that communicates with at least three pneumatic foramina that penetrate the bone. Ventral to the prezygapophyses, the CPRL is laterally convex and there is an oval shaped and pneumatic CPRF. This fossa is delimited medially by a sharp margin. The base of the neural spine is transversely wide. The intervertebral ligament tuberosity is weak, and becomes progressively transversely wider towards the top of the neural spine.

#### Dorsal 3.

This element preserves a partial neural arch with a right transverse process (Fig. 4 A-E). The postzygapophyses are partially preserved. The neural spine is broken but some fragments are observed at the base of the transverse process.

This element is interpreted as a possible D3 based on the presence of a subhorizontally oriented transverse process (similar to D3 of *Murusraptor* and D2 of *Maip*; the transverse process of D4 is more dorsally oriented in *Aerosteon*), proximodistally short transverse process (shorter than D4 of *Aerosteon* but comparable to D3 of *Murusraptor*), laterally oriented transverse process in dorsal view (similar to D3 of *Murusraptor* but contrasting with the anteriorly projected process of D2 of *Maip*), and anteroposteriorly expanded tips of the transverse process (similar to D3 of *Murusraptor*).

The transverse process is less dorsally upturned than in more posterior vertebrae. The transverse process is dorsally subrectangular in contour but expands transversely close to its tip; this condition does not occur in more posterior dorsals. This process is proximodistally short when compared with more posterior vertebrae. The dorsal surface is smooth with some thin longitudinal striations and some rugosities close to its tip. The articular surface of the diapophyses faces ventrally. The postzygapophysis shows a nearly flat articular surface. Dorsal to it, the base of the SPOL is stout and medially concave. The SPOF is deep and shows a step (as in D2 but contrasting with D6). The PO-CDF is poorly preserved but shows, immediately anterior to the postzygapophyses, a deep penetrating pneumatopore.

#### Dorsal 4.

It is represented by an isolated centrum (Fig. 4F–I). This element is identified as the fourth because part of the parapophysis is placed in the neural arch, a single squared or round pneumatopore, as well as the absence of a ventral keel, a condition retained in the first dorsal elements of theropods (such as D1 of *Aerosteon*;^14,34^). Furthermore, this interpretation is tentative giving the lack of articulated dorsal columns in non-juvenile megaraptorid specimens.

The centrum is round or subrectangular being slightly dorsoventrally taller than anteroposteriorly long and with sub-parallel anterior and posterior surfaces. Its ventral and lateral margins are shallowly concave. The posterior articular surface of the centrum is incompletely preserved but seems to be deeply concave, which suggests that the centrum was opisthocoelous. The anterior articular surface of centrum is almost flat and slightly taller than wide. The ventral margin lacks a keel or longitudinal groove, is mostly smooth, and has a strongly rugose anterior surface. The lateral surface of the centrum is longitudinally concave and smooth. At mid-height it shows a deep and round pneumatopore, placed within a shallow pneumatic fossa. Anteriorly, the parapophyses is ovoidal in contour, dorsoventrally taller than anteroposteriorly long. The parapophyses show a rugose surface that might represent the attachment some ligament.

#### Dorsal 5.

This vertebra is represented by a fragmentary neural arch, preserving the right prezygapophysis and the base of the right transverse process (Fig. 5A, C–E). It is interpreted as a D5 because of the presence of an ACDL stouter than in D6, a slightly bigger CDF than in D6, and a slightly smaller parapophysis than that present in D6.

In lateral view, the bases of both CDL can be observed. Whereas the base of the PCDL is robust, the ACDL is gracile (albeit not as gracile as in more posterior dorsal vertebrae). The CDF is deep, subtriangular in contour and exhibits a pneumatopore that penetrates the vertebra. The prezygapophyses are short and stout, and their articular surfaces are subtriangular in contour. The articular surface of each prezygapophysis is flat to slightly convex and slightly medially facing. The CPRL is located ventral to the prezygapophysis. This lamina is relatively short, straight, anteroposteriorly. The CPRF is subcircular in contour and deep. The parapophyses are notably wide and, as in D6, the articular surface is sigmoidal in contour. An oval and deep pneumatopore is present between the ACDL and the prezygapophyses.

#### Dorsal 6.

This vertebra is represented by an incomplete neural arch, the right transverse process, the postzygapophyses and the base of the neural spine (Fig. 5B, F–I). The tip of the transverse process and diapophyses are incompletely preserved.

It is identified as a D6 because of the following characters: parapophyses placed between the centrum and neural arch (placed in the centrum in D3 of *Murusraptor* and D4 of *Aerosteon* but placed in the neural arch in D7 of *Murusraptor* and D8 of *Aerosteon* and *Murusraptor*). The ACDL is much shorter than the posterior one (subequal in length at the anteriormost dorsals of other Megaraptoridae but absent posterior to 10^th^ dorsal). The transverse process is slightly dorsally oriented (laterally oriented in D3 of *Murusraptor* and D4 of *Aerosteon* but more strongly dorsally oriented in D7 of *Murusraptor* and D8 of *Aerosteon* and *Murusraptor*).

In lateral view, the transverse process is subtriangular in contour in cross-section. The articular surface of the process is rounded in contour, slightly concave and ventrolaterally facing. The process shows a rugose anterior surface that probably constitutes the insertion of the ligaments that attach the rib to the vertebra^39^. Posteriorly, the transverse process shows at least three pneumatic foramina closer to its tip. The PODL is notably stout and sub-vertically oriented. The PO-CDF is deep and shows a pneumatopore just anterior to the postzygapophysis. This fossa occupies the preserved posterior surface of the neural arch. Ventral to this surface and placed between the PCDL there is a strongly rugose surface covered with round pits as well as a pair of foramina. The PO-CDF is deep and pneumatic in nature because presence of two strong pneumatopores below the postzygapophyses. The PCDL is long, robust and slightly posteriorly concave. Close to the tip of the transverse process, this lamina connects with the rugose surface that constitutes the insertion of a ligament. This rugose surface is delimited by a small and round concavity. The ACDL is notably thinner and shorter than other laminae, representing one third of the thickness of the PCDL. The CDF is subtriangular in contour, deep and exhibits two pneumatopores: one subrectangular and placed dorsal to the parapophyses and the other one ovoid in contour and placed between the junctions of both CDL. The PRDL is subvertically oriented, deep and nearly straight. The PR-CDF is deep, subrectangular in contour and pneumatic in nature. The parapophyses are subcircular in contour and relatively large, occupying almost two thirds of the anteroposterior length of the neural arch. The parapophyses are asymmetrical in shape (probably owing to taphonomic deformation); whereas the right one is saddle-shaped, the left one is cup-shaped. Saddle shaped parapophyses constitutes an autapomorphy of this species. The articular surfaces of the postzygapophyses are rounded or squared in contour. The right postzygapophysis shows a pneumatic foramen on its posterior margin. The SPOLs are thick and laterally concave. They are separated by a deep, subtriangular-shaped SPOF. The hyposphene is broken and shows a pneumatic foramen on its right side.

There are well-developed CPRL. It is proximodistally short, ventrolaterally oriented and reaches the lateral margin of the neural arch. Ventral to the prezygapophyses, there is a subcircular and deep CPRF. The PRSF is poorly preserved. The neural canal is subcircular in contour and taller than wide. The hypantrum is very wide and deep. A moderate intervertebral ligament tuberosity is observed on the posterior surface of the neural spine. These tuberosities are thinner ventrally but become progressively transversally wider dorsally.

#### Dorsal 7.

This element is represented by a partial neural arch exhibiting a complete left transverse process and the left postzygapophyses (Fig. 6).

It is identified as the seventh dorsal because of the presence of: a moderately upturned transverse processes (less dorsally projected than D8 of *Aerosteon* or D7 and D8 of *Murusraptor* but similar to D6 described above); a transversely thin hyposphene (thinner than in D4 of *Aerosteon* but comparable to D8 of *Aerosteon* and D7 of *Murusraptor*); and deep, abruptly excavated and moderately narrow SPOF (much deeper and narrower than in D4 of *Aerosteon* and D6 of *Maip* and shallower and wider than D8 of *Aerosteon* and D7 and D8 of *Murusraptor*).

In anterior view, the PRDL is thick and straight. The transverse process of this element is long and subtriangular in outline when viewed from the side. The expanded distal end of the transverse process is rugose, probably representing muscular or ligament attachments. The articular surface of the diapophysis is ventrolaterally facing and shows a subtriangular rugosity*.* The PCDL is wide and straight. The PODL is stout. The PO-CDF is deep and close to its dorsal margin it shows small foramina of probable neurovascular nature. Anteromedial to the postzygapophyses, a set of small pneumatic cavities is present. Two pneumatic channels are situated deep within the PO-CDF: one posterior that connects the postzygapophyses to each other, and one anterior which perforates the bone.

The postzygapophysis shows a nearly flat articular surface. The hyposphene is transversely thin and dorsoventrally tall (taller than in D6). The SPOF, dorsally delimits the postzygapophyses. It is notably deep, and subtriangular in contour.

#### Dorsal 9.

This vertebra is represented by a nearly complete element. The neural spine is strongly distorted and its apex is incomplete. The neural arch lacks the tips of the right transverse process (Fig. 7), and the right postzygapophysis is broken. The centrum is incomplete and distorted on its posterior half.

It is identified as a D9 because of the presence of dorsally upturned and long transverse processes (observed in D10 and D11 of *Aerosteon*), anteroposteriorly stout transverse process (stouter than D7 of *Maip* and D8 of *Aerosteon* but resembling D10 of the same taxa), parapophyses placed entirely on the neural arch (a condition present in the posterior half of the dorsal series), presence of a stout PCDL and weak ACDL (observed in D8 of *Aerosteon* but not in D10), and centrum taller than wide, as observed in D8 of *Aerosteon* (but not in more posterior dorsals).

The vertebral centrum in lateral view is subrectangular in contour and slightly taller than long. Its lateral and ventral margins are deeply concave. The pneumatic fossa hosts two pneumatopores. The anterior one is wide and suboval in contour (anteroposteriorly longer than dorsoventrally tall). The posterior pneumatopore is smaller and suboval in outline.

The anterior and posterior articular surfaces of centrum are strongly laterally projected and show striations for the attachment of the intervertebral ligaments. The posterior surface is nearly flat and the anterior one is concave. The ventral surface is smooth and shows numerous neurovascular foramina.

In lateral view, the transverse process is strongly upturned and oriented perpendicular relative to the anteroposterior axis of the vertebra (both features contrast with D6-7 of *Maip*, which show moderately upturned and slightly posteriorly oriented transverse processes). In cross section, the transverse process is subtriangular. The articular surface of the diapophysis is oval in contour (anteroposteriorly longer than dorsoventrally high), slightly concave and ventrolaterally facing. Anteriorly, the transverse process shows a stout, prominent and dorsally concave SDL. Laterally, this lamina becomes anteroposteriorly lower, rugose and dorsoventrally expanded. Ventrally, this lamina is connected with a short PRDL. This lamina is “L” shaped in lateral view and shows a small projection closer to its anterodorsal margin. Close to its tip, the transverse process shows strong transverse striations along its anterior, dorsal and posterior margins. The diapophyses are surrounded by rugosities. The diapophyses show a strong and rugose ventral projection that shows concave anterior and posterior surfaces. The PODL are straight laterally but, on its medial half, the shaft of these laminae becomes ventrally curved when viewed posteriorly. At mid-length to the transverse process, it is observed an oval and teardrop-shaped surface which encloses at least two oval foramina. Ventrolaterally, this teardrop-shaped surface is delimited by a raised, slender and rugose area. The PCDL is notably stouter than the ACDL. On both sides of the vertebra, the laminae are asymmetrical in shape, at the left side the PCDL shows a subtriangular ventral half that is not present at the right side. The ACDL is reduced and more ventrally placed on the left side of the vertebra. The CDF are also asymmetrical, being notably reduced on the left side but much wider on the right. Indeed, the right side shows three pneumatic openings. The parapophyses are large and rounded in contour, occupying almost half the anteroposterior length of the neural arch. The parapophyses are asymmetrical being saddle-shaped on the right side and cup-shaped on the left one. The anterior margin of the right parapophysis is rugose. It is posterodorsally delimited by a concave, smooth and obliquely oriented surface that probably represents the insertion of the costovertebral ligament^40^. The articular surfaces of the postzygapophyses are round or subrectangular, ventrally concave and smooth. The hyposphene is transversely thin and dorsoventrally high. The SPOLs are stout and laterally concave. They are separated by a deep, pneumatic and subtriangular SPOF.

The prezygapophyses are stout and strongly anteriorly projected. Its articular surface is subtriangular in contour, nearly flat and dorsally oriented. Medial to the base of the prezygapophyses there is a small, and probably pneumatic, foramen. Ventral to the prezygapophysis is a well-developed CPRL. This lamina is short and stout, ventrolaterally oriented, and reaches the lateral margin of the neural arch. Ventral to the prezygapophyses a subcircular and deep CPRF is present. The hypantrum is wide and deep. The neural canal is ovoid, being dorsoventrally taller than transversely wide. The PRSF is deep, subtriangular in contour and pneumatic. The PRSL is thick and slightly ventrally expanded. The PRSL, together with the SDL and the PRDL, delimit a subtriangular and deep accessory fossa. A notably anteriorly projected intervertebral ligament rugosity is observed on the anterior surface of the neural spine.

#### Dorsal 10 or 11.

This element consists of an isolated centrum, lacking the posterior articular surface and the posteroventral projection (Fig. 8). This vertebra is interpreted as D10 or D11 based on the presence of a strong projection of the lateral and ventral margins of its anterior and posterior articular surfaces (deeper than D8 of *Aerosteon* but similar to D10 of *Aerosteon* and D11 of *Murusraptor, Tratayenia* and *Aerosteon*), a deep pneumatopore and a blind fossa immediately posterior to it (present in D10 and D11 of *Aerosteon* and D11 of *Murusraptor*).

In lateral view, the vertebral centrum of D10 or 11 is subrectangular and longitudinally concave. The lateral wall is smooth with some obliquely oriented striations, representing the insertion of the intervertebral ligaments, close to the anterior and posterior articular surfaces. In anterior view, the centrum is oval in contour, being slightly taller than wide, and weakly concave. The ventral surface of the centrum is smooth and shows some neurovascular foramina. The anterior articular surface of the centrum is strongly ventrally projected.

In lateral view it shows a deep pneumatic fossa with an anterior pneumatopore and a posterior blind fossa, both separated by an oblique septum. The pneumatopore is very deep, oval in contour and much wider than the blind fossa.

### Caudal vertebrae

Caudal vertebrae are represented by two well-preserved neural arches belonging to the most proximal part of the tail. These vertebrae are very similar in morphology as well as its associated laminae and fossae. The transverse process is horizontally and strongly posteriorly oriented. Below this structure are observed three laminae: the PRDL (anteriorly), the ACDL (anteroventrally) and the PCDL (posteroventrally). These laminae delimits other three fossae: the PR-CDF (anteriorly), the CDF (middle) and the PO-CDF (posteriorly). The PO-CDF is divided by a short but deep accessory PCDL, which has not been observed in other megaraptorids and therefore, could be interpreted as an autapomorphy of *Maip*.

**Figure 9:**
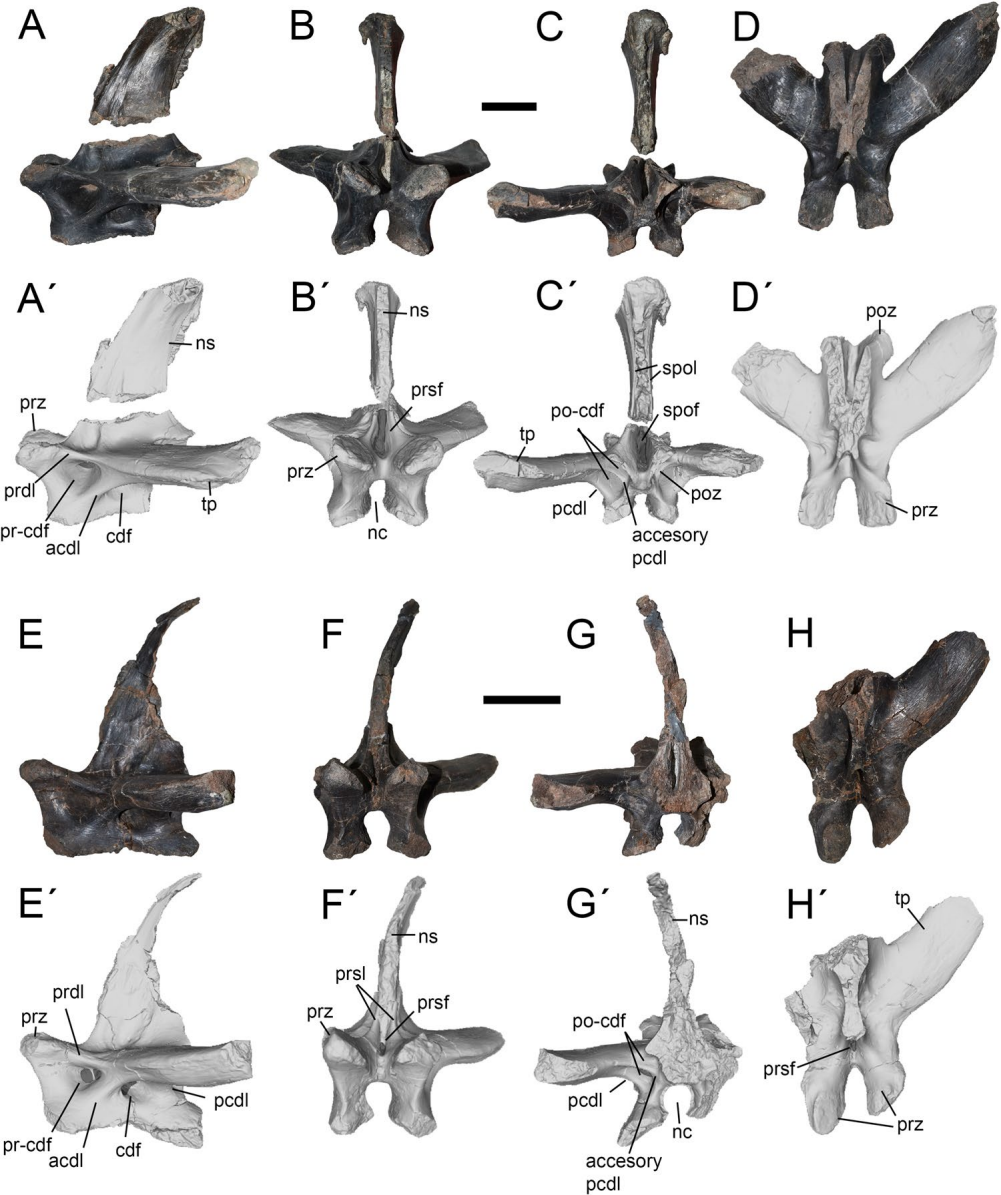
Caudal fourth (**A**-**D**) and caudal seventh (**E**–**H**) of *Maip*. Lateral (**A, A´, E, E´**), anterior (**B, B´, F, F´**), posterior (**C, C´, G, G´**) and dorsal (**D, D´, H, H´**) views. Scale bar: 5 cm. Abbreviations: acdl; anterior centrodiapophyseal lamina; accessory pcdl, accessory posterior centrodiapophyseal lamina; cdf; centrodiapophyseal fossa; nc, neural canal; ns, neural spine; pcdl; posterior centrodiapophyseal lamina; poz, postzygapophyses; po-cdf; postzygapophyseal-centrodiapophyseal fossa; prdl; prezygodiapophyseal lamina; prsf; prespinal fossa; prsl, prespinal lamina; prz, prezygapophyses; pr-cdf; prezygapophyseal-centrodiapophyseal fossa; spof; spinopostzygapophyseal fossa; spol; spinopostzygapophyseal lamina; tp, transverse process.

A proximal caudal is represented by its neural arch and neural spine, lacking the tip of the right transverse process. (Fig. 9A–D). It is tentatively assigned as to Ca4 because of the dorsolateral orientation of the transverse process, the great height of the neural spine, and the strong anterior projection of the pre- and postzygapophyses (as is observed in *Aoniraptor, Megaraptor, Aerosteon* and *Murusraptor*).

The prezygapophyses are short, robust and slightly dorsally upturned. Their articular surfaces are ovoid and face dorsomedially. The PRSF is deep and transversely narrow, and within this fossa there is a subtriangular pneumatopore as occurs in *Aoniraptor*^13^. The left process is completely preserved and becomes dorsoventrally thicker towards its tip. It shows strong striations along its dorsal and ventral margins. On its anteroventral corner, the process shows an oval and rugose concavity that probably constitutes the insertion of the *m. ilio-ischiocaudalis*^41,42^. The PR-CDF is subtriangular and pneumatic. The CDF is rounded, pneumatic and striated. The PO-CDF is oval and shows a deep pneumatopore. The articular surfaces of the postzygapophyses are ovoid in contour and are ventrolaterally facing. The SPOLs are stout and delimit a wide and pneumatic SPOF. The neural spine is dorsoventrally tall, and becomes notably thick towards its apex. The neural spine is strongly posteriorly inclined and shows well-developed intervertebral ligament rugosities. A shallow groove extends along the anterolateral margin of the neural spine. This groove ends at the base of the neural spine in a shallow fossa. In posterior view, the neural canal is dorsoventrally taller than transversely wide.

A more distal caudal (tentatively recognized as Ca7) is represented by an incomplete neural arch, which lacks the right transverse process, the postzygapophyses and part of the neural spine (Fig. 9E–H). It is referred to the proximal region of the tail because of the presence of wide and strongly posteriorly oriented transverse process and a tall and sub-vertically oriented neural spine.

The prezygapophyses are short, stout, and slightly upturned. The articular surfaces are slightly convex, show a subquadrangular contour, and are medially facing. The SPRL is transversely thin, dorsoventrally tall and subparallel to each other. The SPRF is deep and transversally narrow. The transverse process becomes slightly thicker towards its tip. It shows longitudinally oriented striations close to its tip. On its anteroventral margin, the transverse process shows longitudinal rugosities and striations. Ventral to the transverse process there are short and relatively stout ACDL and PCDL. The CDF is ovoid in contour, deep and pneumatic. Anterior to this lies the PR-CDF, which is smaller than the CDF, subtriangular in contour and also pneumatic. The PRDL is short and sub-horizontally oriented. Posteriorly, the PO-CDF is ovoidal in contour, relatively small and pneumatic. The PCDL delimits a blind and subtriangular-shaped concavity. The neural spine is transversally thin, tall and shows smooth lateral surfaces, with exception of a subtriangular-shaped and raised area at its mid-height and that is covered with striations, probably representing a muscle attachment. On its anterior surface, the neural spine shows subtle intervertebral ligament rugosities. The neural canal is ovoidal in contour, dorsoventrally taller than transversely wide.

### Cervical ribs

The cervical rib 5 is represented by its proximal end. It is assigned to CR5 on the basis of the presence of a short and small tubercle, absence of an anterior process, and presence of a large capitulum, a combination of features present in CR5 of *Allosaurus, Tyrannosaurus*^43,44^ and CR5 of *Aerosteon* and CR6 of *Megaraptor*.

The rib head is small compared with cervical ribs 6 and 8. The lateral surface of the rib head is smooth. The tuberculum is short, and shows slightly convergent margins towards the tip. It shows a convex articular surface that exhibits pneumatic foramina on its lateral margin. In anterior view, the tuberculum and capitulum form a right angle to each other. The capitulum is much anteroposteriorly wider but transversely shorter than the tuberculum. The articular surface of the capitulum is flat or rugose and subquadrangular in contour. The anterior pneumatic fossa is deep, dorsoventrally tall and suboval in outline. The ventral surface of the capitulum is smooth and shows pneumatic foramina close to its articular surface. Medially, a straight and thick transverse lamina connects the tuberculum with the capitulum. The posterior pneumatic fossa is deep and ovoid in contour. The shaft of the rib is rod-like and exhibits a longitudinal and striated concave area immediately posterior to the capitulum.

**Figure 10:**
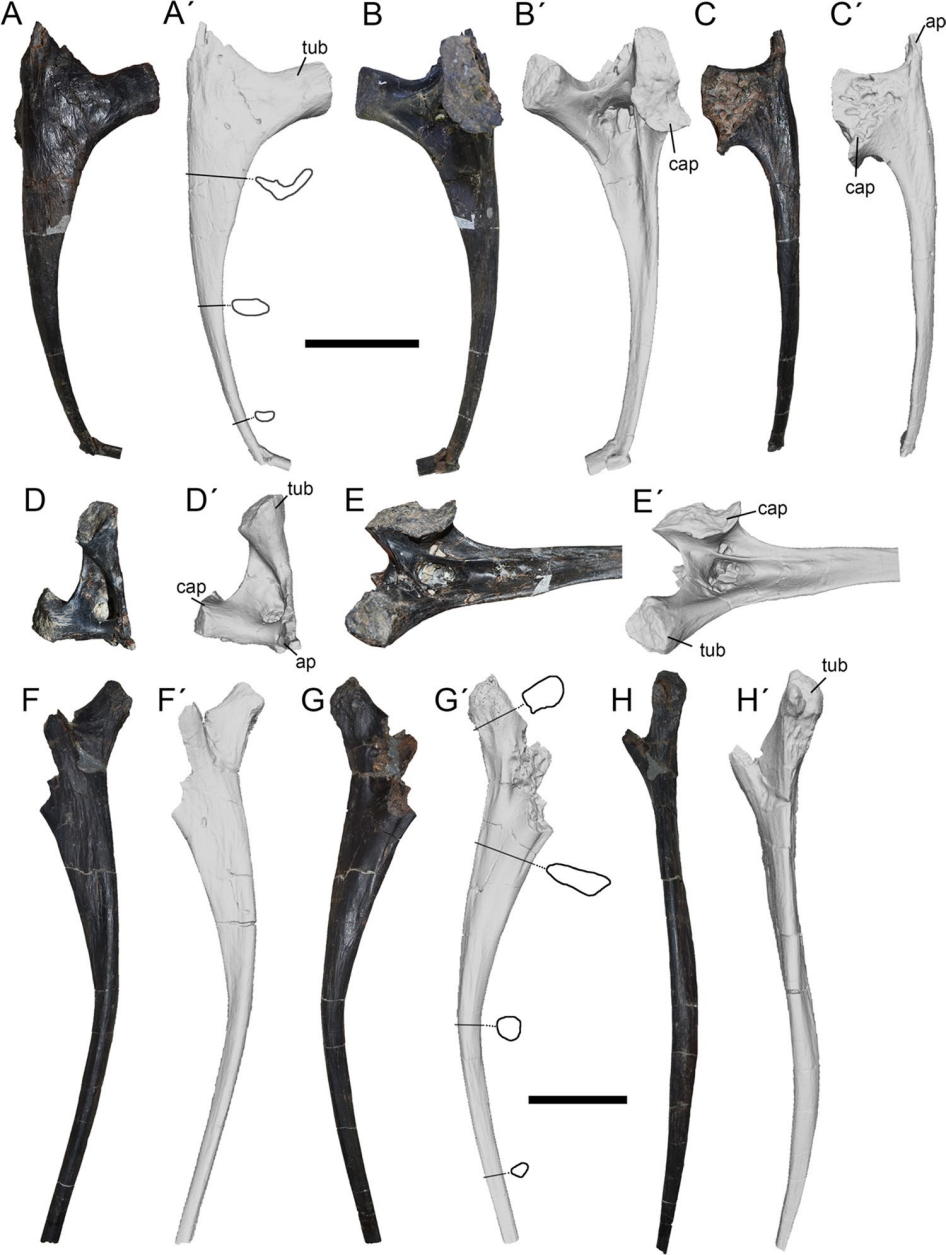
Seventh (**A**–**E**) and eighth (**F**–**H**) cervical ribs of *Maip*. Seventh cervical rib in lateral (**A, A´**), medial (**B, B´**), ventral (**C, C´**), anterior (**D, D´**) and posteromedial (**E, E´**) views. Eighth cervical rib in lateral (**F, F´**), medial (**G, G´**) and dorsal (**H, H´**) views. Scale bars: 5 cm. Abbreviations: ap, anterior process; cap, capitulum; tub, tuberculum.

The cervical rib 7 only lacks its distal tip (Fig. 10A–E). It is assigned to a CR7 on the basis of a well-developed proximal head (absent in anterior ribs); presence of a pointing and well-developed anterior process (present at the mid-length of the neck in *Allosaurus* and *Tyrannosaurus*), as well as a prominent and strongly projected tubercle; the shaft is notably curved (as in posterior ribs) and the tubercle and capitulum form a nearly right angle when viewed anteriorly (at it occurs in ribs of the mid-length of the neck in *Allosaurus*).

The rib head is large, subtriangular in lateral view, and anteroposteriorly long. The lateral and ventral surfaces of the rib head are flat, decorated only with some small longitudinal striations. Medially, in cross-section the rib head is deeply concave being “C”-shaped on its anterior half while is straight and “I”-shaped more distally. The anterior process is subtriangular and sharp. Also, such a process exhibits an almost straight ventral margin and a deeply concave anterior margin. The anterior pneumatic fossa is deep, subtriangular in contour and anteriorly facing. The tuberculum is elongate, thick and rod-shaped, with nearly straight anterior and posterior margins. The tuberculum is much longer and thinner than the capitulum. Its articular surface is dorsolaterally facing, rounded in contour, slightly concave and rugose. Close to its articular surface, the tuberculum shows a small and rugose concavity. In anterior view, the tuberculum and the capitulum form an almost right angle. A stout transverse lamina with a concave dorsal margin connects both structures. This lamina is slightly posteriorly oriented. In medial view, this lamina is perforated by an opening that communicates the anterior pneumatic fossa with the posterior one (see below). The capitulum is slightly eroded, it is ovoidal in cross-section and shows a slightly saddle-shaped dorsally faced and rugose articular surface. The capitulum has longitudinal striations close to its articular surface. Posteriorly, the capitulum shows two ovoid pneumatic foramina that are laterally delimited by a curved lamina that connects the capitulum with the rib shaft and, medially, with a thin and concave ridge. The posterior pneumatic fossa is anteroposteriorly long and becomes shallower posteriorly.

Distally, the shaft of the rib is very thin and delicate and becomes abruptly dorsoventrally thinner and slightly ventrally curved. The shaft shows some longitudinal striations close to the tip of the anterior process. In cross-section, the rib is proximally “V”-shaped and becomes ellipsoidal towards its dorsal half.

A left cervical rib probably corresponding to the C8 is almost completely preserved, lacking its distal tip and part of its proximal head (Fig. 10F–H). It is interpreted as CR8 because it has a notably elongate shaft (albeit shorter than the first dorsal rib) that is wider than that of CR7 and that is ventrally curved (a condition observed in ribs of the base of the neck of other theropods^43^), as well a long and obliquely oriented tuberculum.

The rib head is smaller than in the sixth rib. The tuberculum is slightly anteriorly inclined and strongly offset from the rib head. The anterior margin of the tuberculum is slightly concave and the posterior one is obliquely oriented, straight and dorsally oriented. The articular surface of the tuberculum is convex. The medial surface shows rugosities close to its articular surface. This articular surface shows a posteriorly facing and rugose concavity. Near the base of the tuberculum, part of a pneumatic fossa (probably the posterior one) is preserved. At mid-length, the shaft becomes abruptly thin and rod-like and curves ventrally. This morphology occurs in the posterior half of the neck in other theropods^43–45^. The lateral surface of the rib shaft is flat and dorsoventrally tall anteriorly and becomes progressively shallower posteriorly. The medial margin of the shaft is nearly flat, with a longitudinal rugosity all along its dorsal margin.

Proximally the rib shaft is subrectangular in contour, being transversely compressed, and becomes subcircular in contour at its distal half.

### Dorsal ribs

**Figure 11:**
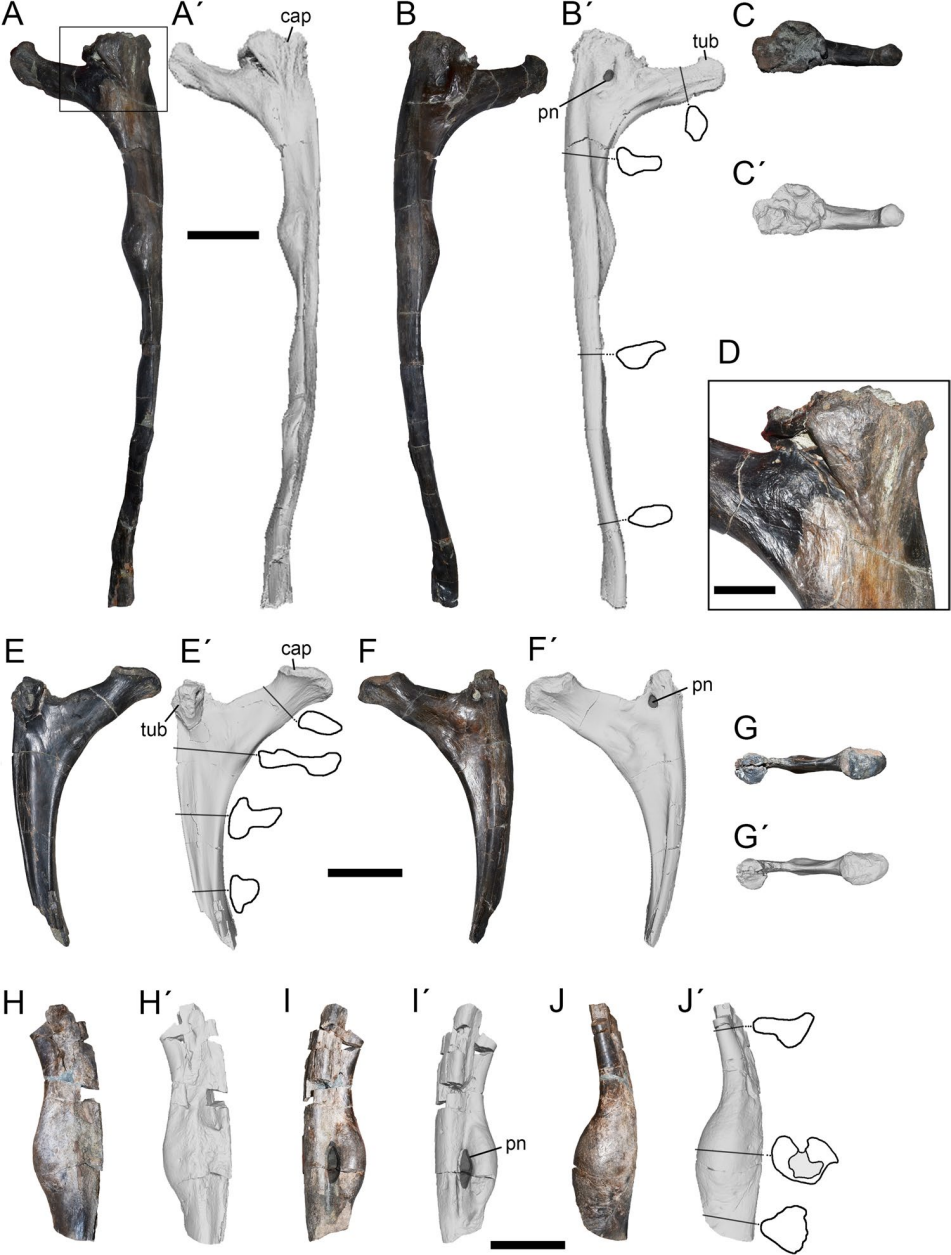
First (**A**–**D**), second (**E**–**G**) and sixth (**H**–**J**) dorsal ribs of *Maip*. First dorsal rib in anterior (**A, A´**), posterior **(B, B´**) and proximal (**C, C´**) views and close-up of the proximal end (**D**). Second dorsal rib in anterior (**E, E´**), posterior (**F, F´**) and proximal (**G, G´**) views. Sixth dorsal rib in anterior (**H, H´**), posterior (**I, I´**) and medial (**J, J´**) views. Scale bars: 5 cm. Abbreviations: ap, anterior process; cap, capitulum; pn, pneumatopore; tub, tuberculum.

A probable first left dorsal rib is almost completely preserved, lacking only its distal end (Fig. 11A–C). It is identified as a first dorsal rib, because of a strong proximal concavity, a slightly medially concave shaft and the presence of one flange on its posterior side and two flanges on its anterior side (as in *Australovenator* but also other theropods such as *Allosaurus* or *Tyrannosaurus*;^43,44^).

The rib head is robust (much stouter than in the cervical ribs) and anteroposteriorly wide, with a long and dorsally upturned capitulum. The dorsal margin of the rib head is deeply concave; its medial margin is concave and has a dorsoventrally oriented and deep and narrow concavity. The dorsal margin of the rib head projects anteriorly forming an anteromedial flange. Below the tuberculum, the rib head inflates as a strong anteroposterior thickening. This thickening affects the entire rib head and becomes progressively wider through the dorsal part of the head. In anterior view, the rib head shows a dorsoventral oblique groove which is delimited by a subparallel ridge. Within this groove is observed a set of aligned pneumatopores. Such pneumatopores become progressively larger dorsally, forming a honey-comb structure. This condition is observed only in this theropod while other theropods shows apneumatic ribs (such as *Allosaurus,* carcharodontosaurids, tyrannosaurids, non-avian paravians, among others;^43–48^) or shows only one pneumatic opening (like abelisaurids;^49^). The posterior surface of the rib head is covered with strong rugosities. Dorsally, the thickening of the rib head is strongly expanded being almost twice the anteroposterior width of the articular surface of the tuberculum. This thickening shows a deep proximal concavity above the head. The tuberculum is dorsally projected, strongly anteroposteriorly thickened and exhibits a rugose articular surface. This surface is oval in contour, concave and dorsally facing. Laterally, the tuberculum shows a rugose bump. At mid-height, the tuberculum shows two foramina within a deep and rugose concavity. The articular surface of the capitulum is rounded in contour. Along its ventral margin, the capitulum shows an anteriorly facing concavity covered by striations and rugosities.

The rib shaft is almost straight and shows an anterior concavity delimited by anteromedial and anterolateral bony flanges. Posteriorly, the rib shaft shows a sharp posterolateral flange that becomes weaker and loses at the mid-height of the rib. The posterolateral flange delimits a posterior concavity.

Proximally, the rib shaft is “L”-shaped in cross section, and becomes ellipsoidal towards its distal end.

A second right dorsal rib is represented by its proximal end (Fig. 11D–F). It is identified as a second rib because of the presence of a mediolaterally long capitulum (longer that in DR1 of *Murusraptor* and *Australovenator* but shorter than in DR2 or 3 of *Australovenator*), a shallow proximal concavity (deeper than DR1 but similar to DR2 or 3 of *Australovenator*), and the presence of one posterior and two anterior longitudinal flanges on the rib shaft.

The rib head is a thin plate, concave at its central part but strongly concave ventrally and laterally. Dorsally, the head is straight with aligned capitulum and tuberculum. Below the capitulum and in the ventral margin of the rib head, are observed a shallow concavity and a deep slender concavity. Posteriorly the rib head is covered by ventromedially–dorsolaterally oriented striations. Ventral to the tuberculum, on the anterior surface of the rib head is observed an oval rugose surface. The tuberculum is dorsally projected, squared in contour and separated from the capitulum by a deep concavity. The tuberculum shows a round but flat articular surface. Below the tuberculum it is observed a big and subtriangular pneumatopore. From the medial side of the tuberculum, rises a strong, dorsally projected and rugose ridge, which projects ventromedially reaching the posterior surface of the rib head. Furthermore, the lateral side of the tuberculum shows a rugose and laterally projected bump. Both structures (ridge and bump), represents the insertions of the costovertebral ligaments^40^. On its posterior surface, the tuberculum shows a raised surface delimited by a round lip and enclosing numerous small foramina (probably of pneumatic origin). The capitulum is dorsomedially facing and shows an expanded and rugose articular surface for the parapophysis. The articular surface of the capitulum is oval in contour (mediolaterally wider than anteroposteriorly long) and saddle-shaped (laterally concave and medially convex). Immediately lateral to the capitulum is observed a short ridge surrounded by striations. Also, a set of rugosities is observed surrounding the articular surface of the capitulum as a ring. Anteriorly, the capitulum shows two sets of striations: one of weak and oblique striations placed laterally to the capitulum and one of almost horizontal and strong striations placed below the capitulum. Both sets of lineations are considered as the insertion of the costovertebral ligament. In anterior view, from the shaft of the rib rises the anterolateral flange, which is deep and runs along the lateral margin of the shaft. On the medial side of the shaft, it is observed the anteromedial flange; which is sharp but weaker than the anterolateral one. Both flanges delimit an anterior concavity and, contrarily to the posterior side, do not intersect each other. Adjacent to the tuberculum and in the posterior face of the rib, it is observed the posterolateral flange which runs along the lateral margin of the bone and following its shaft. This flange is very deep and delimits a posterior concave surface. The preserved part of the rib is “8”-shaped in cross-section.

A fragmentary rib is identified as a possible left sixth dorsal rib, mainly because of its big size (Fig. 11G–J). It is only represented by an incomplete proximal part of shaft. It is notably stout, much more than other ribs. Its proximalmost preserved part shows a “T” shaped cross-section, with concave anterior and posterior margins. The anterior surface is deeper than the posterior one. Ventrally, the shaft becomes strongly thickened forming a hyperostosed “belly”. This belly is posteromedially projected and is thicker at mid-height. Posteriorly, it shows a deep and ovoidal pneumatopore that communicates with a big internal pneumatic chamber and with the posterior concave surface. The surface of the belly is covered with striations and rugosities. A low but stout posterolateral flange runs along the entire length of the rib.

### Gastralia

The total number of gastral ribs in *Maip* is unknown. The length of the largest and most completely preserved medial gastral rib is about 45 cm, with a reconstructed length of roughly 60 cm. The widest point of the thorax of *Maip* (at the level of D6) would have had 140 cm in transverse width.

#### Medial elements

**Figure 12:**
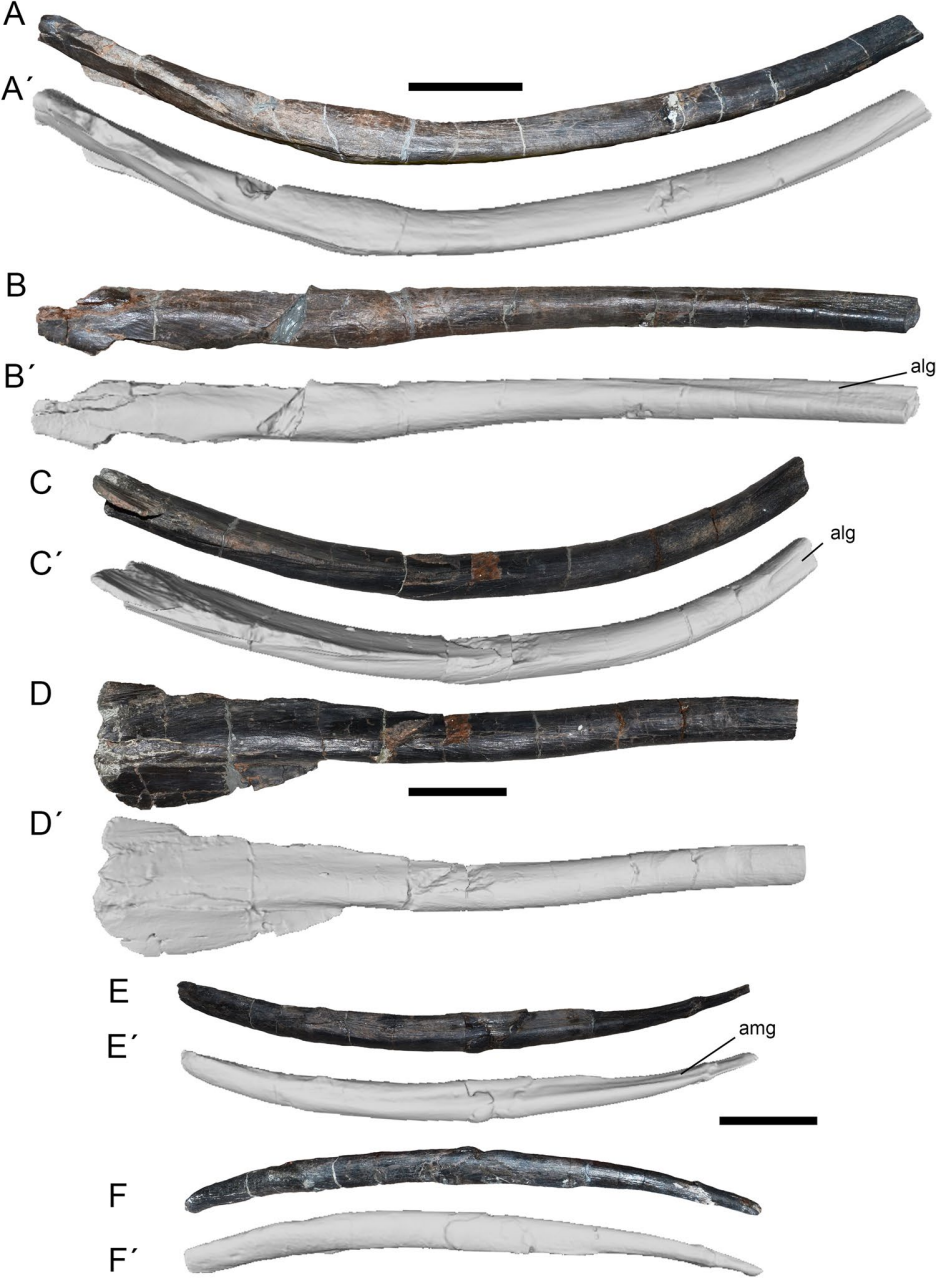
Medial (**A**–**D**) and lateral (**E**–**F**) gastral elements of *Maip* in anterior (**A**, **C**, **E**) and dorsal (**B**, **D**, **F**) views. Scale bar: 5 cm. Abbreviations: alg, articulation surface for lateral gastralia; amg, articulation surface for medial gastralia.

These elements are dorsally curved with a thin shaft and a paddle-shaped medial end (Fig. 12A–D). The anterior medial elements show stouter and less anteroposteriorly curved shafts. The shaft becomes thinner towards its lateral end. In cross-section, the shaft is subcircular or ovoidal at mid-length and becomes sub-quadrangular towards the lateral end. Towards the medial end, the shaft becomes dorsoventrally thinner because of the presence of a medial paddle which constitutes the surface for articulation with the opposite medial gastral element. Anteriorly, the shaft shows the articular surface for the lateral gastral element, which is represented by a concave or flat surface that may reach a fifth of the entire total length of the element. The paddle-shaped medial end shows an anterior flange that is anterodorsally projected and a posterior one that is posteroventrally oriented. Consequently, the medial end of the element is posterodorsally facing. This posterodorsal surface articulates with the anteroventral surface of the posterior gastralia, resulting in the typical theropod interwoven gastral arrangement^43,44^.

#### Lateral elements

These elements are rod-like and gently curved (Fig. 12E,F). The longest preserved element reaches 30 cm. The shaft is oval in cross-section and exhibits its widest point close to the lateral end. Its medial end is relatively thin and shows a concave articular surface with the medial gastral element that is exposed in posterior view. This surface does not reach half of the total length of the element. The lateral end of the element is relatively stout and ends in a rounded surface.

### Pectoral girdle

#### Coracoid

**Figure 13:**
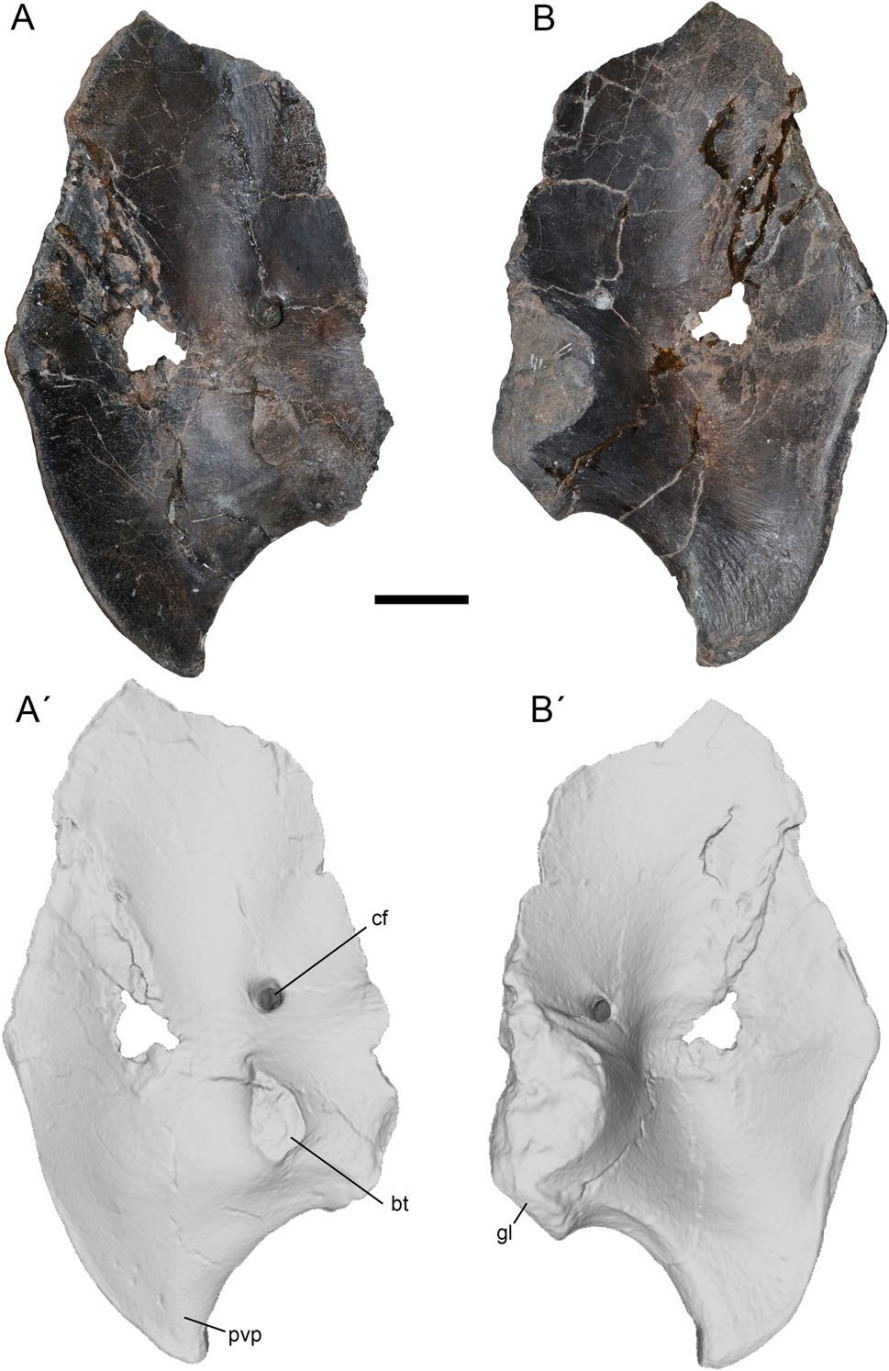
Coracoid of *Maip* in lateral (**A, A´**) and medial (**B, B´**) views. Scale bar: 5 cm. Abbreviations: bt, biceps tubercle; cf, coracoid foramen; gl, glenoid cavity; pvp, posteroventral process.

The left coracoid is almost completely preserved, only lacking its dorsal margin and most of the biceps tubercle (Fig. 13). This bone is very thin with the exception of its posterior half, which shows a strong buttress that was continuous with the scapula. In lateral view the coracoid is ovoidal in contour, being more than twice as tall dorsoventrally as it is long anteroposteriorly. The lateral surface is smooth and concave. Medially, the coracoid is deeply concave, this concavity being deepest at its center. The scapular articulation is rugose.

The anterior margin of the coracoid is convex and shows a prominent anterior projection, which is absent in other megaraptorans (such as *Fukuiraptor, Megaraptor* or *Aerosteon*). Dorsal to this projection, the anterior margin shows rugosities for muscular attachment, and ventrally it shows a posteromedially facing articular surface. This surface appears to be absent in other megaraptorans such as *Fukuiraptor, Aerosteon* and *Megaraptor*. Based on its position it is possible to infer that it constituted the articular surface for the sternum. No signs of the sternum has been recovered in *Maip *and other megaraptorans, and it is probable that it was mostly cartilaginous or poorly ossified.

Only the base of the bicep tubercle was preserved, but it seems to be robust and not reduced as in *Aerosteon*^34^. The coracoid foramen is moderate, ovoidal and subvertically oriented. Posteriorly to the foramen, there are strong anteroposteriorly oriented striations that represent the anchoring of the *m. biceps brevis*^50^. In medial view, the coracoid foramen is ovoidal, posteriorly oriented and close to the limit with the articulation with the scapula. It does not form a groove connecting with the scapula as is observed in *Aerosteon*^14^. The glenoid cavity is straight and does not project laterally to form a lip. The posteroventral process is prominent, subtriangular in contour when viewed from the side, and ventrally projected. It shows on its medial side, strong and curved rugosities that are subparallel to the margins of such a process**.** Such structures may correspond with the attachment of the *m coracobrachialis*^50^. *Maip* shows a relatively simple posteroventral process that lacks the vertical lamina, subglenoid ridge and deep subglenoid fossa present in *Aerosteon*^14^. A subglenoid fossa is very shallow and poorly defined in *Maip*.

## Discussion

### The costovertebral ligaments in *Maip*

**Figure 14:**
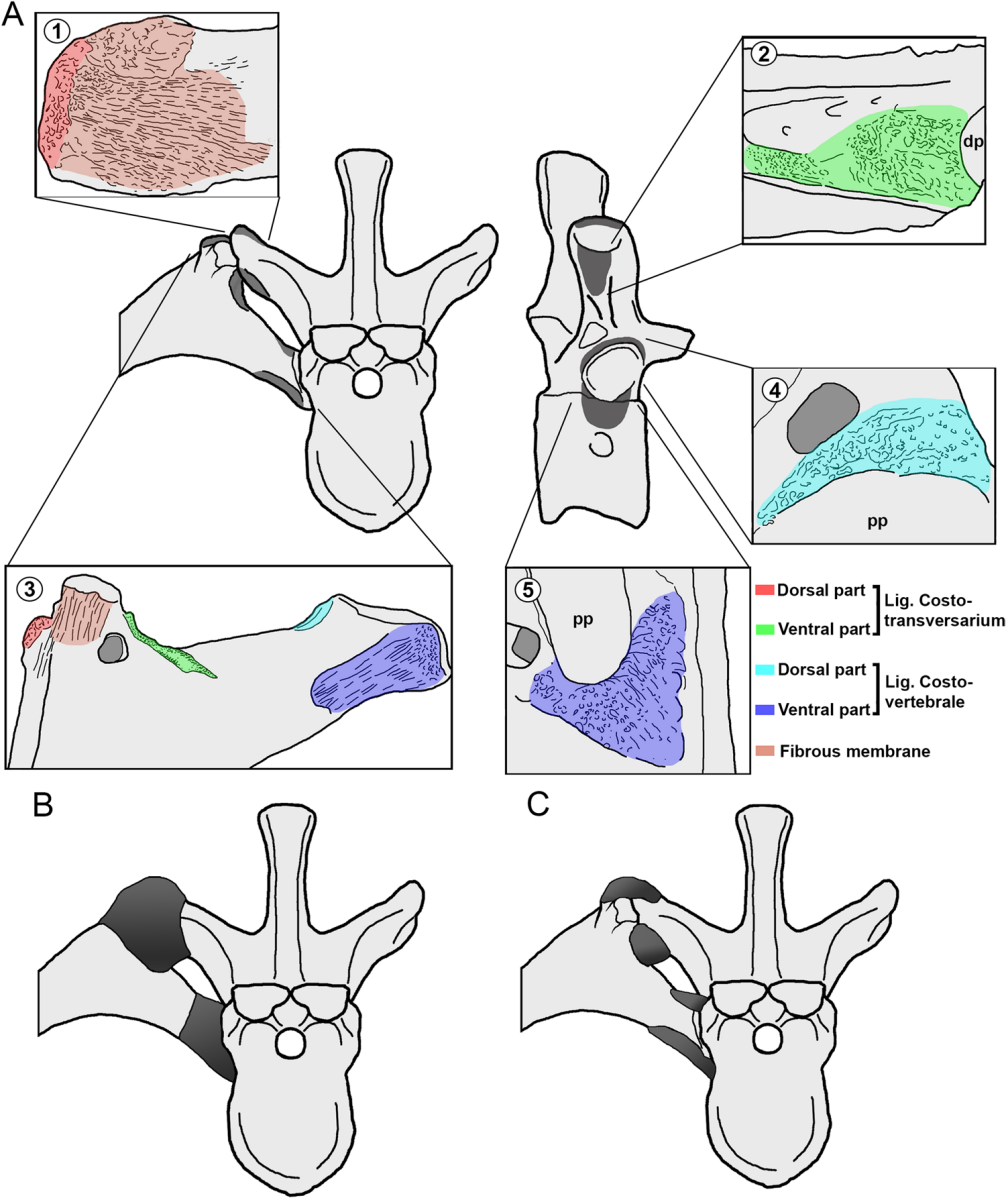
Reconstruction of costovertebral ligaments in dorsal vertebra and its correspondent rib of *Maip*. (**A**), mid-dorsal vertebra showing the scars for costovertebral ligaments. Transverse process in dorsal (1) and ventral (2) views. Dorsal rib (3) in anterior view. Dorsal vertebra in lateral view (4–5). (**B**), reconstruction of costovertebral ligaments (including the fibrous membrane) of a mid-dorsal segment. (**C**), reconstruction of costovertebral ligaments (excluding the fibrous membrane) of the same segment. Figure not to scale. *dp* diapophysis; *pp* parapophysis.

As commented in the description, many vertebrae and ribs of *Maip* show striations or rugosities which should be interpreted as the attachment sites for the costovertebral and costotransversarium ligaments (Fig. 14). This condition is not commonly observed in other theropods, and thus, *Maip* offers the rare opportunity to discuss the costovertebral ligaments in these dinosaurs. In most aspects, these attachments for the costovertebral ligaments resemble the condition described for vertebrae and ribs of tyrannosaurids^40^.

In *Maip,* vertebrae and ribs exhibit striations surrounding the tips of the transverse processes of dorsal vertebrae which are interpreted as the insertion site of the *lig. costotransversarium*.

In most amniotes, the *lig. costovertebrale* is composed by a fibrous membrane that wraps the tip of the transverse process of vertebrae and the tuberculum of the ribs. This ligament act as a sleeve-like structure with two main thickenings (one dorsal and one ventral) which bears all the weight of the bone^40^. These thickenings are stronger parts of the ligament which, by supporting all the weight, leave strong marks in the bone^40^. In tyrannosaurids, the anterior and posterior sides of the tip of the transverse process, as well as its dorsal surface show strong transverse striations, and a raised projection surrounded by concavities and ridges on its ventral side evidence the presence of the *lig. costotransversarium*^40^. As the ribs is concerned, the insertion of this ligament has been inferred in tyrannosaurids by the presence of transverse striations on the anterior and posterior surfaces of the tubercle, a raised and obliquely oriented ridge medial to the tubercle, and a rugose bump-like projection on the lateral margin of the rib^40^. In *Maip,* the transverse processes of D3, D6 and D7 exhibit smooth longitudinal striations at the anterior and posterior faces, as well as strong longitudinal striations on its dorsal surface, which may correspond to the fibrous membrane (Fig. 14A1). The transverse processes show rugosities closer to its tip (Fig. 14A1) and a raised rugose projection immediately ventral to the diapophyses (Fig. 14A2); the former is interpreted to be the dorsal part of the *lig. costotransversarium*, whereas the latter is interpreted as its ventral part. Similarly, the tuberculum of DR1, DR2 and other indeterminate proximal ribs, shows striations along its anterior and posterior surfaces, which may correspond to the fibrous membrane. An obliquely and rugose ridge at the medial side of the tubercle, may represent the insertion of the dorsal part of the *lig. costotransversarium* and a round, raised and rugose bump lateral to the tuberculum, may corresponds to its ventral part (Fig. 14A3). This suggests that, as in tyrannosaurids and birds^40^, the costovertebral joint of *Maip* was covered by a fibrous membrane, and that the *lig. costotransversarium* had two main parts placed at the dorsal and ventral parts of the joint between transverse processes and rib tubercles. Hirasawa^40^ noted that this condition is related with a kinetic joint between vertebrae and rib.

The anchoring of the costovertebral ligaments is evidenced on the vertebrae and ribs of extant birds by means of scars surrounding the vertebral parapophyses and rib capitulum^40^. In *Maip*, some vertebrae (axis, D2, D4-D6 and D9) show strong rugosities surrounding the parapophyses (Fig. 14A4,5). In D4 such rugosities are placed anteroventral to the parapophyses, forming a crescent-shaped surface, probably being the ventral part of the *lig. costovertebrale* (Fig. 14A4). In the axis, D2, D5-D6 and D9 the rugosities are observed dorsally as a narrow and anteroposterior elongate area which probably represents the anchoring of the dorsal part of the ligament (Fig. 14A5). The capitulum of DR1-2 as well as other indeterminate elements shows obliquely oriented striations on its posterior surface, which could represent the fibrous membrane of the joint. Along the ventral surface of the capitulum there is a concave area with strong distal striations and small proximal rugosities; at the dorsal surface of the capitulum there exists a subtriangular area with a raised ridge and smooth rugosities, which corresponds to the dorsal part of the ligament (Fig. 14A3). In contrast to the *lig. costotransversarium*, the scars for the *lig. costovertebrale* are less clearly defined; however, based on preserved scars this ligament probably had dorsal and a ventral attachment sites.

When ribs and corresponding vertebrae of *Maip* are articulated together, the four sets of striae (two per ligament) become aligned in an obliquely oriented main axis. This may have allowed rib rotation when the animal was alive, and therefore, it was able to expand and contract the thoracic cavity during respiration, as was previously reconstructed for tyrannosaurids^40,51,52^. This kind of rib movements is correlated with some other morphological traits present in most tetanuran theropods *Maip* such as the presence of long and upturned transverse process of dorsal vertebrae, ventrolaterally oriented diapophyses and long and curved dorsal ribs with long and well-separated capitula and tubercula^40,51,52^.

It is possible that the ligament system related to advanced respiratory rib movements was widespread among tetanurans. However, because striations surrounding transverse processes of dorsal vertebrae and the capitula and tubercula of dorsal ribs are subtle, they might be easily lost by erosion.

### The Campanian through Maastrichtian megaraptorid record from southern Patagonia

*Maip*, from the Chorillo Formation, is not the only record of Megaraptoridae from the uppermost Cretaceous, although it is currently the most complete. In addition to *Maip,* the Chorrillo Formation has yielded at least four additional megaraptorid specimens: an isolated dorsal centrum (MPM 21,546); an isolated tooth (MACN-Pv 19,066); and two sets of associated teeth (MPM-PV-22864–5^3,39^). It must be said, however, that comparisons of the isolated dorsal centrum (MPM 21,546) with unenlagiids, such as *Unenlagia,* better suggests that this bone might pertain to Unenlagiidae rather than to Megaraptoridae.

Teeth recovered from the Chorrillo Formation (MACN-Pv 19,066, MPM-PV-22864–5) exhibit a similar size and morphology to those of medium- to large-sized megaraptorids, such as *Murusraptor, Orkoraptor,* and *Megaraptor*. MPM-PV-22864 and MPM-PV-22865 are almost identical in morphology differing, as expected, in those traits that vary within the anterior and posterior parts of the jaw (such as the symmetry or not of the tooth crowns). Conversely, one tooth (MACN-Pv 19,066) differs from the rest in having a high density of distal denticles (5 per mm vs 3 per mm in all other megaraptorids^3,39^). It should be noted that MACN-Pv 19,066 was collected in the area in 1980 but lacks specific stratigraphic and geographic provenances^3^.

In sum, *Maip* is the most informative megaraptorid recovered from the Chorillo Formation and the only with enough diagnostic anatomical traits and stratigraphic control to be considered as a new taxon.

Another Maastrichtian unit from Patagonia which yielded megaraptorid remains is the Lago Colhué Huapi Formation, in Central Patagonia (Chubut Province^20,36,37,53^). These records are: two isolated unguals (UNPSJB-PV 1028 and UNPSJB-PV 1102^36,37^); an associated manual ungual I and metatarsal III (UNPSJB-PV 1046 and UNPSJB-PV 1066^36,37^), and an as yet undescribed skeleton (UNPSJB-PV 1104^53^). Except for the latter specimen, the size of which is unknown, all these records represent medium- to large-sized megaraptorids (more than 6 m long^20^). Nevertheless, the isolated nature of the specimens makes it impossible to identify the ontogenetic age and size of the above lised specimens; thus they do not allow us to determine differences or similarities with *Maip*.

The Cerro Fortaleza Formation underlies the Chorrillo beds, and is often ascribed a Campanian age. This unit has yielded the medium-sized megaraptorid *Orkoraptor* (roughly 6 m long^35^). The main difference between both taxa relay in the respective size of the specimens (6 m vs. 9.5 in *Maip*; see Supplementary Information I and II). Unfortunately, *Orkoraptor* shows minor overlapping materials with the skeleton of *Maip*, being the mid-caudal vertebrae the only elements that are present in the holotype specimens of these two megaraptorids, although the vertebral positions are not the same. *Maip *differs from *Orkoraptor* by the presence, on its mid-caudal vertebrae, of deep PR-CDF, horizontally disposed pre- and postzygapophyses, anteroposteriorly long hypantrum, and strong and stout transverse processes. All these differences can be considered as valid, but we are cautious because they are not based on exactly corresponding caudal vertebrae.

In sum, *Maip macrothorax* is the first megaraptorid taxon to be named for the Maastrichtian of southern Patagonia, representing the biggest among currently known megaraptorids (Lamanna et al.^20^ and Supplementary Information II), and one of the youngest records (alongside with those from Lago Colhué Huapi Formation) for the entire clade^39^.

### *Maip* compared with other megaraptorid and non-megaraptorid theropods

The main difference between *Maip* and all other megaraptorids is its large size. In this regard, when compared with *Aerosteon* (the second largest megaraptorid), the vertebrae of *Maip* are slightly larger but also notably bulkier.

The axis of *Maip* is anteroposteriorly short and dorsoventrally tall. Besides the axis of *Maip*, the other known axis from Megaraptoridae is that of the juvenile of *Megaraptor namunhuaiquii* (MUCPv-595^10^). Compared with *Maip*, the axis of *Megaraptor* is much anteroposteriorly longer and dorsoventrally lower and the postzygapophyses and the neural spine are much transversally wider. Moreover, the morphology observed in *Megaraptor* resembles more to the condition of most theropods (such as *Allosaurus* or *Sinraptor*^43,45^) than that of *Maip*. Within Theropoda, the proportions of the axis of *Maip* more closely resemble tyrannosaurids (such as *Tyrannosaurus*^44^) and carcharodontosaurids (such as *Acrocanthosaurus*), which have anteroposteriorly short axes. The height/width ratio of the axis is 2.1 times in *Maip* and *Tyrannosaurus*, 1.8 in *Acrocanthosaurus* and 1.3 in *Allosaurus*. The intercentrum of *Maip* is notably ventrally placed, being slightly posterior to the parapophyses. This contrasts with most theropods in which this structure is observed anteriorly to the pleurocentrum of the axis. Unfortunately, the axis of the juvenile specimen of *Megaraptor* does not preserve the intercentrum^10^; thus whether or not this condition is unique to *Maip* is unknown. The neural spine of the axis of *Maip* lacks a transversely wide spine table as observed in tyrannosaurids^44,54^. Nevertheless, the neural spine of *Maip* is notably low, being slightly lower than those of *Allosaurus* or *Tyrannosaurus* but much lower when compared with carcharodontosaurids (such as *Acrocanthosaurus* or *Concavenator*^55,56^). In *Maip* the postzygapophyses are rounded, being slightly transversely wider than anteroposteriorly long, which seems the condition for *Tyrannosaurus*^44^. In contrast, allosauroids such as *Allosaurus* and carcharodontosaurids (e.g., *Acrocanthosaurus*, *Giganotosaurus*) show large postzygapophyses that are notably transversely wider than long^33,43,55^. Furthermore, the orientation of the articular surface of the postzygapophyses is sub-horizontal or slightly upturned in *Maip* and other theropods such as *Allosaurus* or *Tyrannosaurus*^43,44^. By contrast, in carcharodontosaurids the articular surface of the postzygapophyses are strongly dorsally oriented. The epipophyses of *Maip* are moderately developed as occurs in many tetanurans^57^; however, in carcharodontosaurids, the epipophyses are notably developed and strongly dorsolaterally projected;^55,56^. In sum, the axis of *Maip* shows a unique morphology which could be autapomorphic of this species or common to its group; until new megaraptorid skeletons come to light this cannot be confirmed. Finally, the axis of *Maip* shows substantial differences with other theropods but particularly with carcharodontosaurids.

The D2 of *Maip* resembles in morphology of comparable vertebrae of *Murusraptor* and *Aerosteon*. However, *Maip* and *Aerosteon* are much bigger than *Murusraptor*. Likewise, the neural arch of the D2 of *Maip* is much stouter and dorsoventrally lower than those of *Allosaurus, Sinraptor* and *Concavenator*^43,45,56^. On the other side, larger theropod species like *Tyrannosaurus* or *Giganotosaurus* and *Mapusaurus* show stout columns that resemble more to those observed in large-sized megaraptorans^33,44,47^. Furthermore, the articular surface of the prezygapophyses of anterior dorsal vertebrae of *Maip* and *Aerosteon* are round, stouter and more anteriorly projected than those of *Murusraptor*. Interestingly, the prezygapophyses of *Maip* are placed below in the neural arch and do not reach the base of transverse process and the base of the neural spine. This condition is shared with the D4 of *Aerosteon* and the first dorsal vertebrae of derived tyrannosaurids^14,44^. In contrasts, the prezygapophyses surpass the dorsal margin of the base of the transverse process in D3 of the mid-sized megaraptorid *Murusraptor*, and the D2 of small-sized tyrannosauroids (such as *Guanlong*^58^), as well as most tetanurans such as *Allosaurus, Sinraptor, Lajasvenator* and *Neovenator*^43,45,59,60^. In sum, the dorsal vertebrae of *Maip* shares some conditions that are only observed in very large theropod species which suggests that these could be size-dependent traits.

In the mid-dorsals of megaraptorids (such as the D6 and D9 of *Maip*, the D7 of *Murusraptor*, the D8 of *Aerosteon* and the articulated column of the juvenile of *Megaraptor*) the transverse process is dorsally directed (or slightly posterodorsally) but not as in most theropods (such as *Allosaurus, Tyrannosaurus* or carcharodontosaurids^43,44,55,56^) in which the transverse processes are strongly posterodorsally projected. This unique condition of megaraptorids is directly related with a more lateral position of the ACDL and PCDL (in most theropods, these laminae are posterolaterally oriented in association with the posterior projection of the transverse process^43,44,55,56^) and the minor size of the PR-CDF. In sum, all megaraptorids share the presence of a less posteriorly oriented transverse process in mid-dorsal vertebrae. This condition has been noted for abelisaurids such as *Carnotaurus* and *Majungasaurus*;^60–62^.

The proximal caudal vertebrae of *Maip* shows tall neural spines. This condition is shared with other megaraptorids (such as *Megaraptor* or *Murusraptor*^6,33^) as well as tyrannosaurids and *Allosaurus*. On the contrary, carcharodontosaurids show taller proximal caudal neural spines^56^. In addition, the dorsoventral height/anteroposterior width ratio of the proximal caudal vertebrae of megaraptorids, tyrannosaurids and *Allosaurus* is less than 2^6,33,43,44^, while in carcharodontosaurids (such as *Mapusaurus* and *Concavenator*^48,56^) the ratio ranges between 2 and 4.

Among megaraptorids, the coracoid is known in *Maip*, *Aerosteon* and *Megaraptor*. *Maip* resembles *Aerosteon* in having a strongly projected ventral process and a deep posteroventral fossa^14^. Notwithstanding, in the above description it was noted that *Maip* exhibits a simpler posteroventral cavity than *Aerosteon*, which results in an autapomorphic character of the former taxon. Furthermore, in *Maip* the coracoid is stouter and anteroposteriorly wider than in *Aerosteon*, being the high/width ratio 1.6 in *Maip* and 2.0 in *Aerosteon*. In sum, the coracoid of *Maip* shows a unique anatomy and proportions, which reinforces its condition as a new taxon.

#### Phylogenetic results

**Figure 15:**
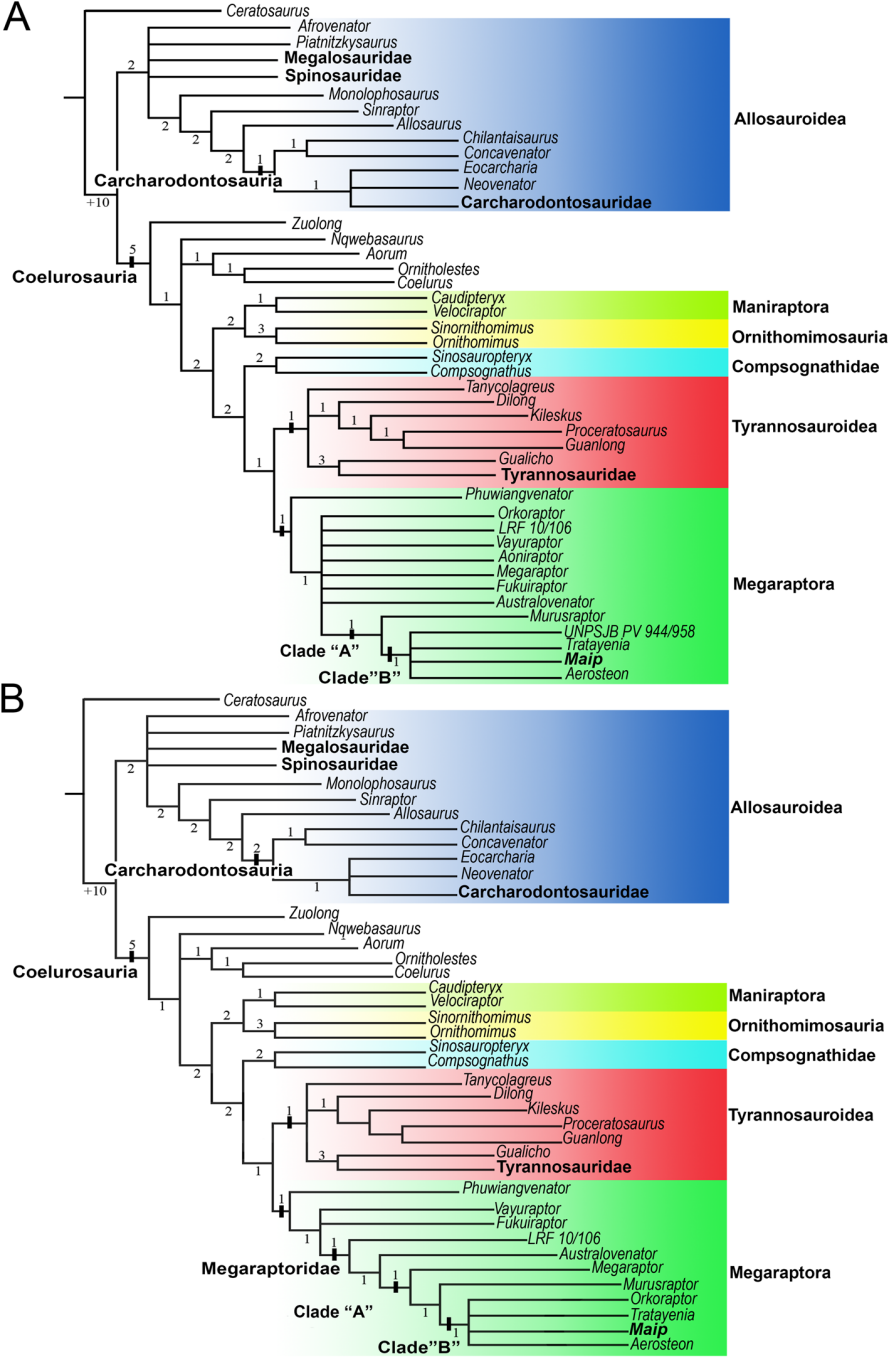
Phylogenetic relationships of *Maip* of the analysis with (**A**) and without (**B**) fragmentary taxa. Numbers represents Bremer values for each node.

With the aim of testing the taxonomic validity of *Maip* as well as its phylogenetic relationships with other theropods, two different phylogenetic analyses were performed (see Supplementary Information I). The analyses with and without fragmentary taxa show a topology similar to those of previous analyses^12,19^, with megaraptorans nested within Coelurosauria, and forming the sister group of Tyrannosauroidea (Fig. 15A).

The analysis including all taxa results in 2560 most parsimonious trees (MPTs) of 1453 steps. Within Megaraptora, this analysis shows *Phuwiangvenator* as the earliest-branching member of the clade, and as the sister taxon to a polytomy including *Vayuraptor*, an indeterminate Australian megaraptorid (LRF 100–106), *Fukuiraptor, Aoniraptor*, *Australovenator*, the indeterminate Patagonian megaraptorid from the Bajo Barreal Formation (UNPSJB-PV-944/958), *Megaraptor* and *Orkoraptor*. These theropods constitutes the sister group to the clade including *Murusraptor, Aerosteon, Tratayenia* and *Maip*.

*Aoniraptor* is resolved as a megaraptoran of uncertain affinities (Fig. 15A), as has been observed in other works^13,32^. The enigmatic theropod *Gualicho*^18^, which in some analyses^63^ has been considered as a possible synonym of *Aoniraptor*, here is deeply nested within Tyrannosauroidea, but far from *Aoniraptor* (Fig. 15A–B). Furthermore, 17 extra steps are required to move *Gualicho* as the sister taxa to *Aoniraptor*. In sum, following Aranciaga Rolando et al*.*^13^, we conclude that both taxa are not closely related.

Regarding the indeterminate megaraptorid from Bajo Barreal Formation (UNPSJB-PV-944/958;^20^), this specimen shares with other South American taxa a dorsal vertebral centrum that is anteroposteriorly longer than dorsoventrally tall (Ch. 106-1) and a manual ungual phalanx with a flexor tubercle that shows a wide mediolateral sulcus (Ch. 292-2). In spite of its fragmentary nature, UNPSJB-PV-944/958 is considered to be more closely related to South American forms than with those of other landmasses of Gondwana. Other isolated megaraptoran records from South America are those of the Campanian Uberaba Formation^64^, and Upper Cretaceous sediments of Brazil^31^, which remain as indeterminate megaraptorans because of their fragmentary nature.

The second analysis, after pruning fragmentary taxa with less than 15% of the skeleton, results in 80 most parsimonious trees (MPTs) of 1455 steps (Fig. 15B). The Consistency Index (CI) is 0.312 and the Retention Index (RI) is 0.599. The tree topology is very similar with the first analysis but shows a higher resolution within Megaraptora. As in previous works, the more basal forms of this clade are the Asian *Phuwiangvenator*, *Vayuraptor*, and *Fukuiraptor*; which constitute successive sister taxa to Megaraptoridae^2,7,10,12,19^. Among megaraptorids, the Australian LRF 100–106 and *Australovenator* represent the earliest-branching members of the clade and are more closely related to each other than to South American megaraptorids (See Supplementary Information I). The latter include a clade with taxa from the early Late Cretaceous (*Megaraptor* and *Murusraptor*) are another clade formed by younger Patagonian megaraptorids (*Tratayenia, Orkoraptor, Aerosteon* and *Maip*). Regarding robustness values, Bremer support and bootstrap frequencies (absolute and GC) are relatively low (as in all previous phylogenetic analyses of this clade) and are slightly higher in the analysis when fragmentary taxa are excluded (Fig. 15; Supplementary information I).

#### Recovering two clades of South American megaraptorids

One of the most outstanding results of the present analysis (Fig. 15B) is the recovering of two new clades (the first including the second one) comprising some derived megaraptorids from South America. The more inclusive clade (Clade “A”) comprises *Megaraptor, Murusraptor* and the less inclusive clade (Clade “B”). This latter includes *Orkoraptor, Tratayenia, Aerosteon* and *Maip*.

**Figure 16:**
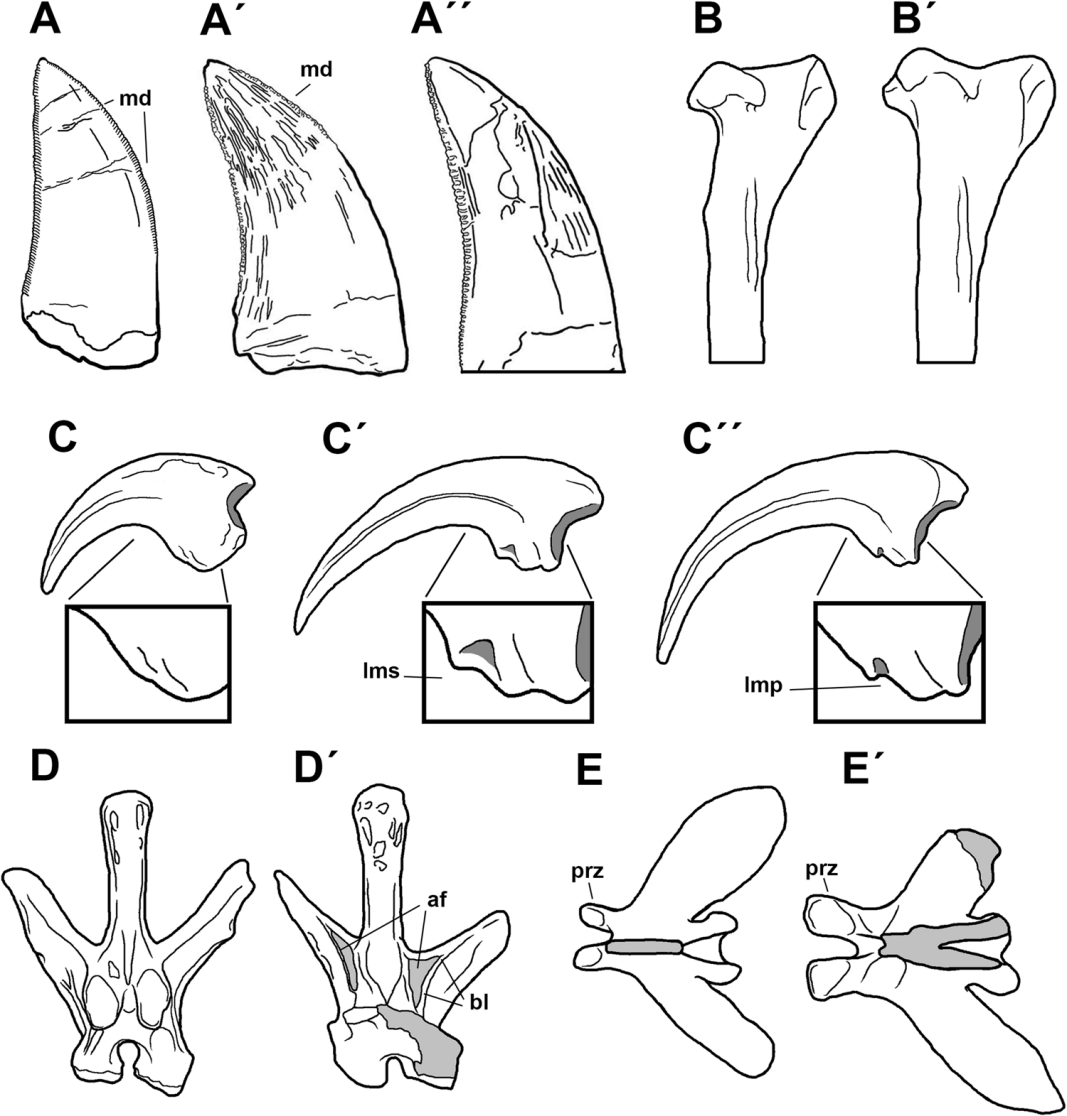
Comparative drawings showing the features that supports Clade “A” (**A**–**C**) and Clade “B” (**D**–**E**). A, maxillary tooth of *Fukuiraptor* (**A**), *Australovenator* (**A´**) and *Murusraptor* (**A´´**). B, proximal end of tibia of *Australovenator* (**B**) and *Aerosteon* (**B´**). C, manual phalanges and close-ups of their flexor tubercles of *Fukuiraptor* (**C**), *Australovenator* (**C´**) and *Megaraptor* (**C´´**). D, dorsal vertebrae of *Murusraptor* (**D**) and *Tratayenia* (**D´**). E, dorsal view of a mid-caudal vertebra of *Megaraptor* (**E**) and *Maip* (**E´**). Abbreviations: af, accessory fossa; bl, bifurcated lamina; lmp, lateromedial platform; lms, lateromedial sulcus; md, mesial denticles; prz, prezygapophyses.

Clade “A” is supported by three synapomorphies (see SI): (1) Absence of mesial denticles (Ch. 2–2; Fig. 16A): such denticles are observed in *Fukuiraptor, Australovenator* and the isolated megaraptoran teeth from Strzelecki Group of Australia^24^, but they are absent in *Megaraptor, Murusraptor, Orkoraptor* and the megaraptorid tooth from the Chorillo Formation (MACN-Pv 19,066). (2) Tibia, dorsally curved in lateral view (Ch. 355-0; Fig. 16 B) which is observed in *Murusraptor, Aerosteon* and *Orkoraptor* but it is absent in *Australovenator*. (3) Manual ungual phalanges with flexor tubercle with a wide lateromedial and smooth platform (Ch. 292-2; Fig. 16C). This trait is absent (state 0) outside Megaraptora as well as on its basal forms such as *Fukuiraptor,* the Thai taxa and the fragmentary ungual of Strzelecki Group (NMV P186153^42^); instead, the Australian *Australovenator,* LRF 100/106 and Megaraptoridae cf. *Australovenator wintonensis* (NMV P239464^25^) show a mediolateral sulcus (state 1). By contrast, in *Megaraptor,* the megaraptorids from Bajo Barreal Formation (UNPSJB-PV 944;^20^) and Lago Colhué Huapi Formation (UNPSJB-PV 1102 and 1046;^36,37^) and the referred ungual from *Aerosteon*^34^, a wide mediolateral platform is present (state 2).

Clade “B” (including *Maip*, *Aerosteon*, *Orkoraptor* and *Tratayenia*) is supported by two synapomorphies (See SI): 1) Dorsal vertebrae with a bifurcated lamina anterior to the transverse process and forming an accessory fossa (Ch. 352-1; Fig. 16D), which is observed in all the dorsal vertebrae of *Maip, Tratayenia* and *Aerosteon* but not in the Bajo Barreal Formation megaraptorid (UNPSJB-PV-944/958), *Murusraptor* or *Megaraptor*; and 2) Round and large articular facets of pre- and postzygapophyses of proximal caudal vertebrae (Ch. 353–0; Fig. 16E). This condition is observed in the caudal vertebrae of *Maip, Orkoraptor* and *Aerosteon*, contrasting with *Megaraptor, Aoniraptor* and *Murusraptor*, the proximal caudal vertebrae of which have smaller prezygapophyseal facets.

Interestingly, Clade “A” includes most of the Cenomanian–Turonian Patagonian forms. Furthermore, all of these forms exceed 6 or 7 m in length. This supports conclusions by Lamanna et al.^20^, who hypothesized that megaraptorids underwent a trend towards body size increase during the Late Cretaceous. Moreover, Clade “B” gathers Santonian through Maastrichtian megaraptorids from South America, which also constitute the largest known megaraptorans (between 8 and 10 m long,^33,34,64,65^). These new clades reinforce the proposal made by Lamanna et al.^20^ that Patagonian megaraptorans diversified and increased their size during the Late Cretaceous.

#### Other potential characters for the South American megaraptorids

**Figure 17:**
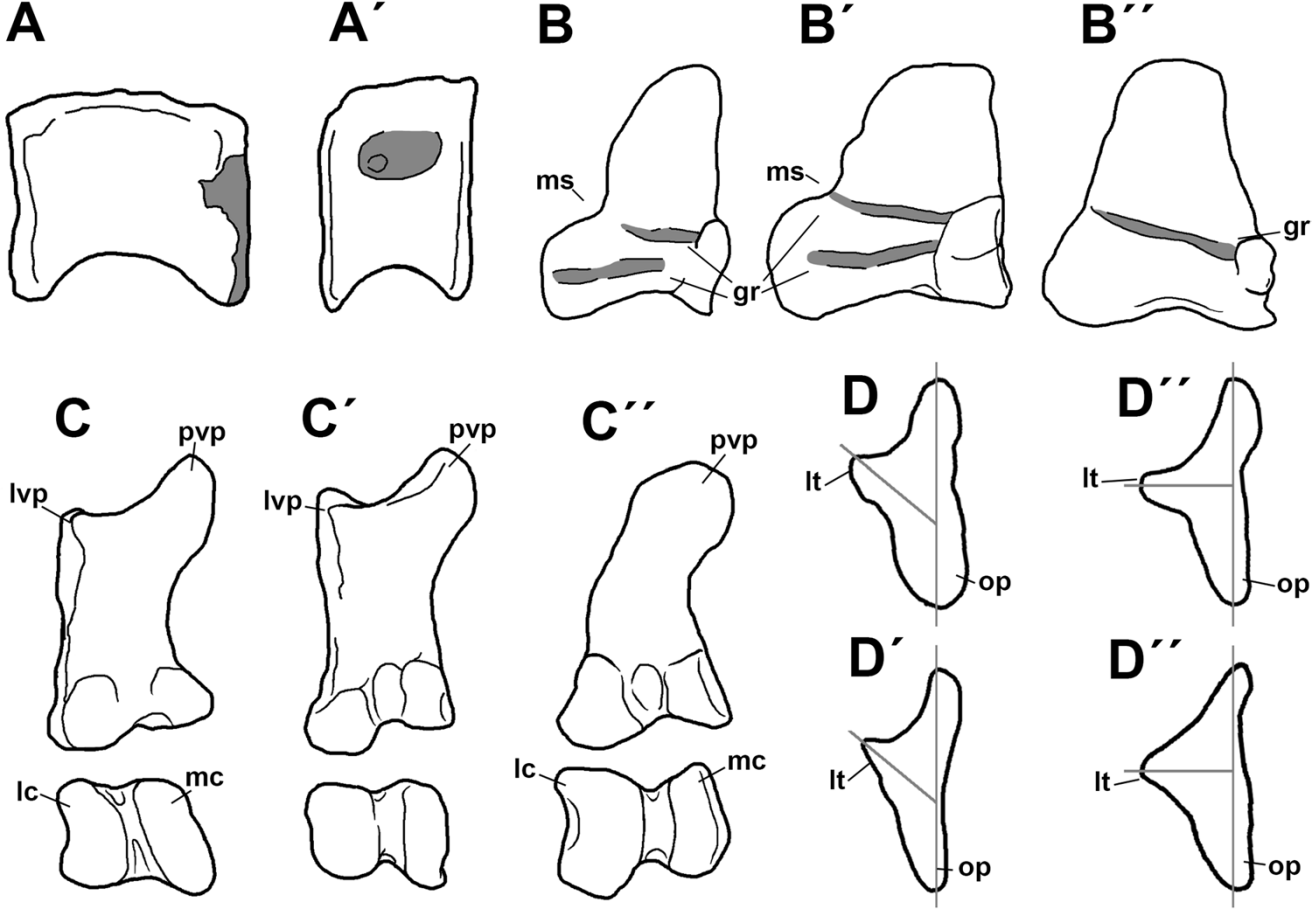
Comparative drawings of potential features supporting a natural clade of South American megaraptorids. A, dorsal vertebra of *Fukuiraptor* (**A**) and *Aerosteon* (**A´**). B, anterior view of astragalus of *Vayuraptor* (**B**), *Australovenator* (**B´**) and *Aerosteon* (**B´´**). C, dorsal (up) and distal (down) views of first of *Australovenator* (**C**), ´*Rapator´* (**C´**) and *Megaraptor* (**C´´**). D, proximal view of ulna of *Australovenator* (**D**), a Megaraptoridae indet from Australia [NMV P186076] (**D´**) and *Megaraptor* (**D´´**). Abbreviations: gr, groove; lc, lateral condyle; lt lateral tuberosity; lvp, lateroventral process; mc, medial condyle; ms, medial shelf; op, olecranon process; pvp, posteroventral process.

In our phylogenetic analyses, we found some other potential traits which might be apomorphies of Megaraptora, but are only observable in a few taxa owing to incompleteness. Nevertheless, these were described here for future analyses. One of these characters is the presence dorsal centra anteroposteriorly longer than dorsoventrally tall (Ch. 106–1; Fig. 17A), a condition that it is shared by *Megaraptor, Murusraptor, Tratayenia, Maip, Aerosteon* and the indeterminate megaraptorid from Bajo Barreal Formation (UNPSJB-PV-944/958; described by Lamanna et al.^20^ as a caudal element, but here reinterpreted as a dorsal centrum). In contrast, the dorsal centra are longer than tall in *Fukuiraptor, Phuwiangvenator* and the isolated dorsal centrum from the Strzelecki Group in Australia (NMV P221187^24^).

Another two features differently distributed among megaraptorans are: presence of two transverse grooves on the anterior surface of the astragalar body, and ascending process not reaching the entire width of the astragalar body, and with a medial “step” in anterior view (Fig. 17B). These features are observed in *Fukuiraptor, Vayuraptor, Phuwiangvenator, Australovenator,* cf. *Australovenator wintonensis* from the Eumeralla Formation (NMV P253701^25^), and an isolated astragalus from Strzelecki Group (NMV P150070^42^). By contrast, the astragalus of *Aerosteon* shows only one groove at the base of the ascending process and the later one extends transversally all the width of the astragalar body, and forming a “step” in anterior view.

On the other side, ‘*Rapator ornitholestoides*’ and *Australovenator*^8,27^ have a metacarpal I with a strongly proximally projected ventrolateral process, a posteroventral process notably more proximally projected than the rest of the bone, and distal articular hemicondyles subequal in size (Fig. 17C). All these features contrast with *Megaraptor*^7^, which lacks of a ventrolateral process, bears a posteromedial process that does not proximally surpass the rest of the bone, and exhibits the lateral hemicondyle notably larger than the medial one. Moreover, the available megaraptorid ulnae from Australia (e.g., NMV P186076, LRF 100/106 and *Australovenator*^8,24,26^) share the presence of a lateral tuberosity dorsolaterally projected in proximal view (Fig. 17D). This feature contrasts with the ulna of *Megaraptor*^6,7^, in which the lateral tuberosity is projected outwards.

#### The diversification of Megaraptora in South America

**Figure 18:**
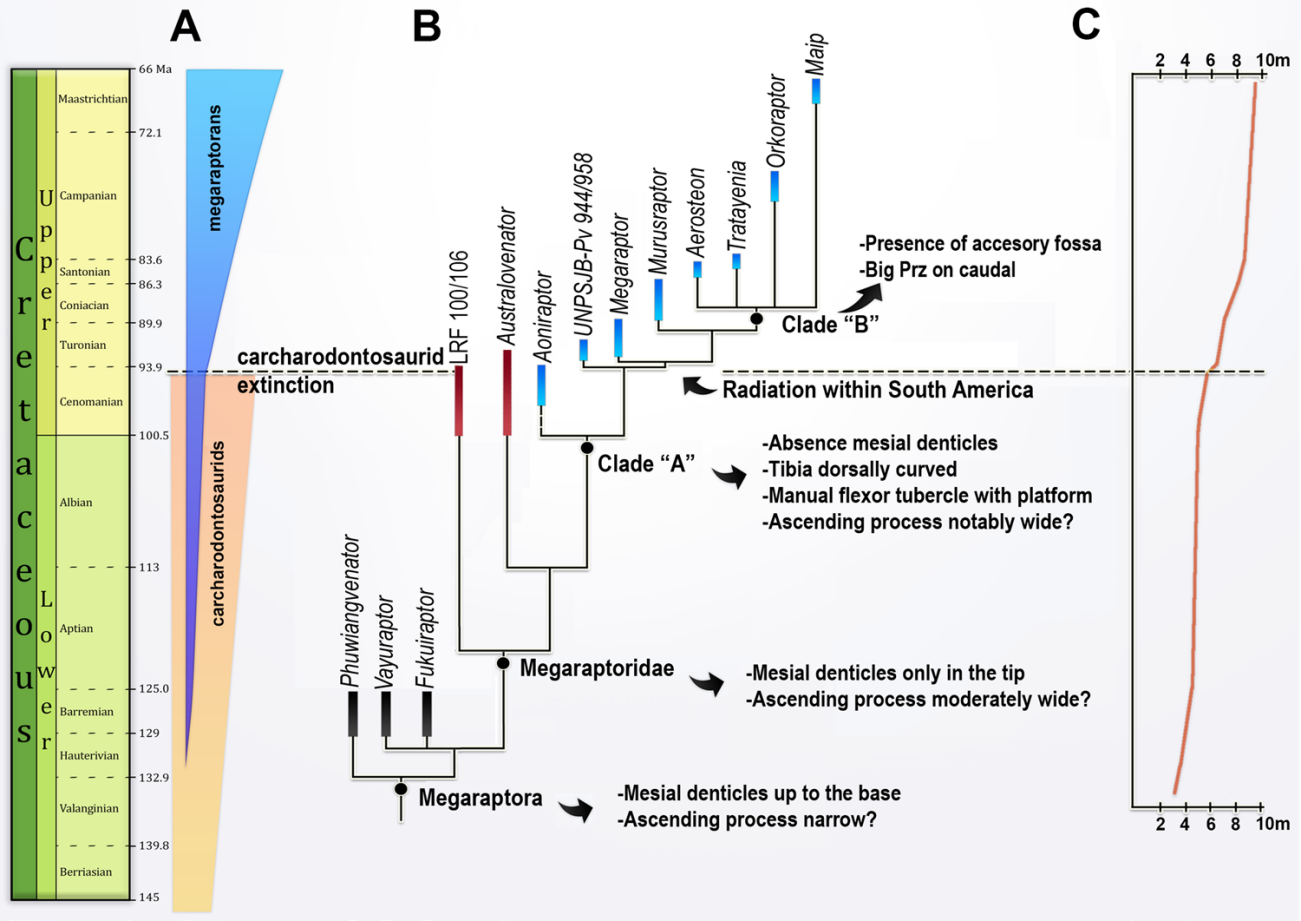
Evolutionary trends of Megaraptora. (**A**) Temporal scale and bars depicting currently known temporal distributions of Megaraptora and Carcharodontosauridae. (**B**) Time-calibrated phylogeny of megaraptoran taxa, showing most relevant genera from Asia (black bars), Australia (red bars) and South America (blue bars). Main synapomorphies supporting each node are indicated by arrows. Tree topology follows the results of the present work. (**C**) Curve showing the increasing in average body size of megaraptorans during Barremian through Maastrichtian time bins.

Lamanna et al.^20^ noted that within South America, megaraptorids exhibit a size increase and a taxonomic diversification in the Late Cretaceous. Our analysis supports this and also, based on our phylogenetic results, we observe a correlation between the morphology, size and age in the megaraptoran records (Fig. 18A–C). Smaller (4–4.5 m), early-branching megaraptoran species evolved during the Barremian–Aptian of Asia, South America and Australia^4,5,11,22,24^. Medium-sized (4.5–6 m) megaraptorids appeared during Aptian through lower Turonian times in Australia and South America^20,24,26,28,29,32^. In the course of the Turonian and Coniacian, the megaraptorids are only known from South America, with medium- to large-size (6–7.5 m) forms more closely related to each other than to other members of the group. Finally, from Santonian through Maastrichtian times, the endemic clade of South American megaraptorids became bigger (8–10 m).

In Turonian–Coniacian sedimentary rocks, records of Megaraptoridae are notably more numerous in South America, suggesting that the group diversified (Fig. 18B), and also came to occupy an important role in Late Cretaceous ecosystems in this continent.

Notably, pneumaticity within the axial skeleton became progressively more developed in more derived megaraptorans. This could be related with a major necessity of making the skeleton lighter but stronger, at the same time, as well as the necessity to reduce the metabolic cost of the skeleton in bigger forms^66,67^. However, quantifying the pneumatic condition of these theropods requires for further analyses.

In sum, our results support the hypothesis advocated by Lamanna et al*.*^20^ that megaraptorids became progressively bigger, much more abundant numerically and taxonomically more diverse, throughout the Cretaceous. Furthermore, we find that within South America, and after the Cenomanian, these theropods experienced a more abrupt size increase (Fig. 18A–C). Notably, the extinction of carcharodontosaurids and spinosaurids—which had been the apex predators in Gondwana^2,68,69^—occured at the end of the Cenomanian.

Several authors have proposed that an extinction event occurred during the Cenomanian–Turonian boundary, affecting many vertebrate groups in both hemispheres^68,70,71^. Some authors discussed the impact of such an event, considering the posibility that this extinction was a step-wise process involving several million of years^69^. Nevertheless, there is a consensus that after the Cenomanian–Turonian time interval, many dinosaurian groups became extinct in tandem with the origin, diversification, and increased numerical abundance of others^2,68,70–73^. For the Northern Hemisphere, several authors proposed an ecological replacement during the Cenomanian–Turonian boundary^16,72,73^. During this interval, carcharodontosaurids (or allosauroids) and tyrannosaurids coexisted, with tyrannosaurids being represented by modest-sized forms occupying the role of small- to mid-size predators, and carcharodontosaurids being larger species and playing the role of top predators^16,72,73^. After the Turonian, carcharodontosaurids went extinct and tyrannosaurids underwent a notable diversification involving a higher number of taxa alongside with a sustained increase in body size, thus occupying the role of apex predators^16,72,73^. We observe that, previous to Cenomanian–Turonian times, small- to mid-sized megaraptorids coexisted with carcharodontosaurids but, after the Turonian, they showed an increase in the body size (Fig. 18D). We hypothesize that megaraptorids (and other theropod groups) replaced carcharodontosaurids in the role of apex predators within the Southern continents in the course of the Late Cretaceous.

Similarly, the record of abelisaurids and unenlagiids shows a body-size increase after the Cenomanian–Turonian extinction^74,75^. This work suggests that, contrary to Laramidia, where carcharodontosaurids were replaced by tyrannosaurids^16,72,73^, in Gondwana a much more diverse array of large theropods, including the megaraptorids, abelisaurids and possibly large-sized unenlagiids (i.e., *Austroraptor*), became the top predators of their ecosystems after carcharodontosaurid went to extinct^12,14,15^.

## Methods

### Description

Several bones were digitally scanned by surface scanning with a Shinning 3D Einscan Pro. The resulting. obj files were then rendered with 3D Slicer software and converted to image files. For description of the column, each vertebrae or rib were nominated with a “D” for dorsal, “Ca” for caudal, “CR” for cervical rib, “DR” for dorsal rib followed of the number of the bone. For the nomenclature of laminae and fossae we use the terminology proposed by Wilson^76^ and Wilson et al.^77^. The abbreviations of the laminae and the fossa of the vertebrae used in the text are the following: ACDL, anterior centrodiapophyseal lamina; CDF, centrodiapophyseal fossa; CPRF, centroprezygapophyseal fossa; CPRL, centroprezygapophyseal lamina; PCDL, posterior centrodiapophyseal lamina; PO-CDF, postzygapophyseal-centrodiapophyseal fossa; PODL, postzygapophyseal lamina; PP, parapophyses; PR-CDF, prezygapophyseal-centrodiapophyseal fossa; PRDL, prezygodiapophyseal lamina; PRSF, prespinal fossa; PRSL, prespinal lamina; SPOF, spinopostzygapophyseal fossa; SPOL, spinopostzygapophyseal lamina; TPOL, intrapostzygapophyseal lamina.

### Phylogenetic analyses

We use the data matrix provided by Aranciaga-Rolando et al.^12^. To this data set, we also added six new osteological characters and some fragmentary megaraptoran taxa (such as *Aoniraptor*, LRF 100–106 and UNPSJB PV-944/958). Two different phylogenetic analyses were performed: one with some fragmentary specimens and one without them (see Supplementary Information I for more details).

### Analyses of theropod faunas of Patagonia

Estimation of body-length of Megaraptora members follows Lamanna et al*.*^20^ (See Supplementary information I and II). *Maip* was approximately nine to ten meters long; its body-length was estimated based on comparisons with the proportions of the vertebrae of *Megaraptor, Aerosteon* and *Murusraptor* (Supplementary information II).

## Supplementary Information

Supplementary Information 1.

Supplementary Information 2.

## Abbreviations

MACN-CH: Museo Argentino de Ciencias Naturales “Bernardino Rivadavia”, Colección Chubut, Ciudad Autónoma de Buenos Aires, Argentina
MPM: Museo Padre Molina, Río Gallegos, Santa Cruz, Argentina
