# Supplementary material for: A large Megaraptoridae (Theropoda: Coelurosauria) from Upper Cretaceous (Maastrichtian) of Patagonia, Argentina
Source: Sci Rep. 2022 Apr 26;12:6318. doi: 10.1038/s41598-022-09272-z (PMC9042913; doi:10.1038/s41598-022-09272-z)
Supplement: Supplementary file 1 — Supplementary Information 1. [file 41598_2022_9272_MOESM1_ESM.docx]

A large Megaraptoridae (Theropoda: Coelurosauria) from Upper Cretaceous (Maastrichtian) from Patagonia, Argentina

Alexis M. Aranciaga Rolando ^1*^, Makoto Manabe^2^, Takanobu Tsujishi^2,3^, Matías J. Motta^1^ *and* Fernando E. Novas^1^.

Supplementary information A

**Size estimation:**

The size estimation in megaraptorids is a big problem giving the absence of, at least, one complete skeleton. Grillo and Delcourt^1^ comments that rule of three is less reliable for estimate body lengths, except when the bone scale isometrically (as for example, vertebrae, hindlimb and scapulocoracoid). In this sense, the most complete specimen is the juvenile of *Megaraptor*, which has many skull bones as well as articulated cervical and dorsal series and both scapulae but lacks most of the tail^2^. This specimen has 1,5 meters between the tip of the skull to the third sacral centrum. Regarding the tail, usually represents the half of the total body length (or slightly more; see Grillo and Delcourt^1^). Consequently, the body length of the juvenile specimen of *Megaraptor* (MUCPv-595) is 3 meters long. With this data, we take nine different measures (five in the sixth cervical, three in the scapula and one in the coracoid; See Fig. S1 and Supplementary information II) between the juvenile and adult specimen of *Megaraptor* (MUCPv-341). These were used to calculate (by a rule of three) an estimated adult size of *Megaraptor* (See Supplementary Information II). The resulted value is 7.63 (consistent with previous estimation of the species^2^; but see also Lamanna et al.^4^).

Furthermore, Sereno et al.,^5^ preliminary estimate the body length of *Aerosteon* in 9-10 meters. We compare the absolute measures (the same used for the previous analysis; see Fig. S1 and Supplementary Information II) of the C5 and the scapulocoracoid of *Aerosteon* with the C6 and the scapulocoracoid of *Megaraptor*, respectively. This results in nine absolute comparisons. With all these, we apply our estimated total body length of *Megaraptor* to calculate that of *Aerosteon*. This results in a total body length of 8.3 meters for *Aerosteon* (slightly minor with the estimated by Sereno et al.,^5^).

**The size of *Maip* vs *Aerosteon* and *Murusraptor***

Calculating the size of megaraptorids is hard and poorly reliable giving the absence of complete skeletons. Thus, preliminary estimations are the best that we have to work with until more complete materials comes to light. With the aim of estimate the size of *Maip* (without using less reliable estimations), we take twelve measurements to this specimen (seven on the dorsal vertebral and five of the coracoid; See Fig. S1 and Supplementary Information II). These values were treated as absolute values and were compared with those of the same elements (or the neighbor element in the case of vertebrae) of other megaraptorids like *Aerosteon* and *Murusraptor*. Finally, an average value was calculated for each bone of *Maip* respect the other megaraptorid. This allow us to quantify how much bigger (or smaller) was this bone.

The D1, D4, D9 and coracoid of *Aerosteon* were compared with the D2, D4, D9 and coracoid of *Maip*, respectively. Twenty absolute comparisons (Fig. S1 and Supplementary Information II) were taken being all bigger in *Maip*. The average value of all measures results that *Maip is* 17% bigger than *Aerosteon*. This data reveals that *Maip* is the biggest known megaraptorid. Furthermore, Sereno et al.^5^ estimate a body length of 9-10 meters to *Aerosteon* (while our own estimation results in 8.3 meters; see Supplementary Information II and the previous paragraph of size estimation). Based in our measurements, the approximate body length for *Maip* is 9.7 meters.

Regarding *Murusraptor*, the D3, D7, D9 and D11 of this specimen were compared with the D2, D6, D9 and D10 or 11 of *Maip*, respectively. Nineteen absolute comparisons were taken, being *Maip* significantly bigger than *Murusraptor*. The average value shows that *Maip* is 47% bigger than *Murusraptor*. Coria and Curie^6^ estimated the size of this latter specimen using hindlimb bones, which results in a total length of 6.5 meters long. Extrapolating this value with the proportion obtained for *Maip*, allow us to estimate a body length of 9.5 meters for this taxon.


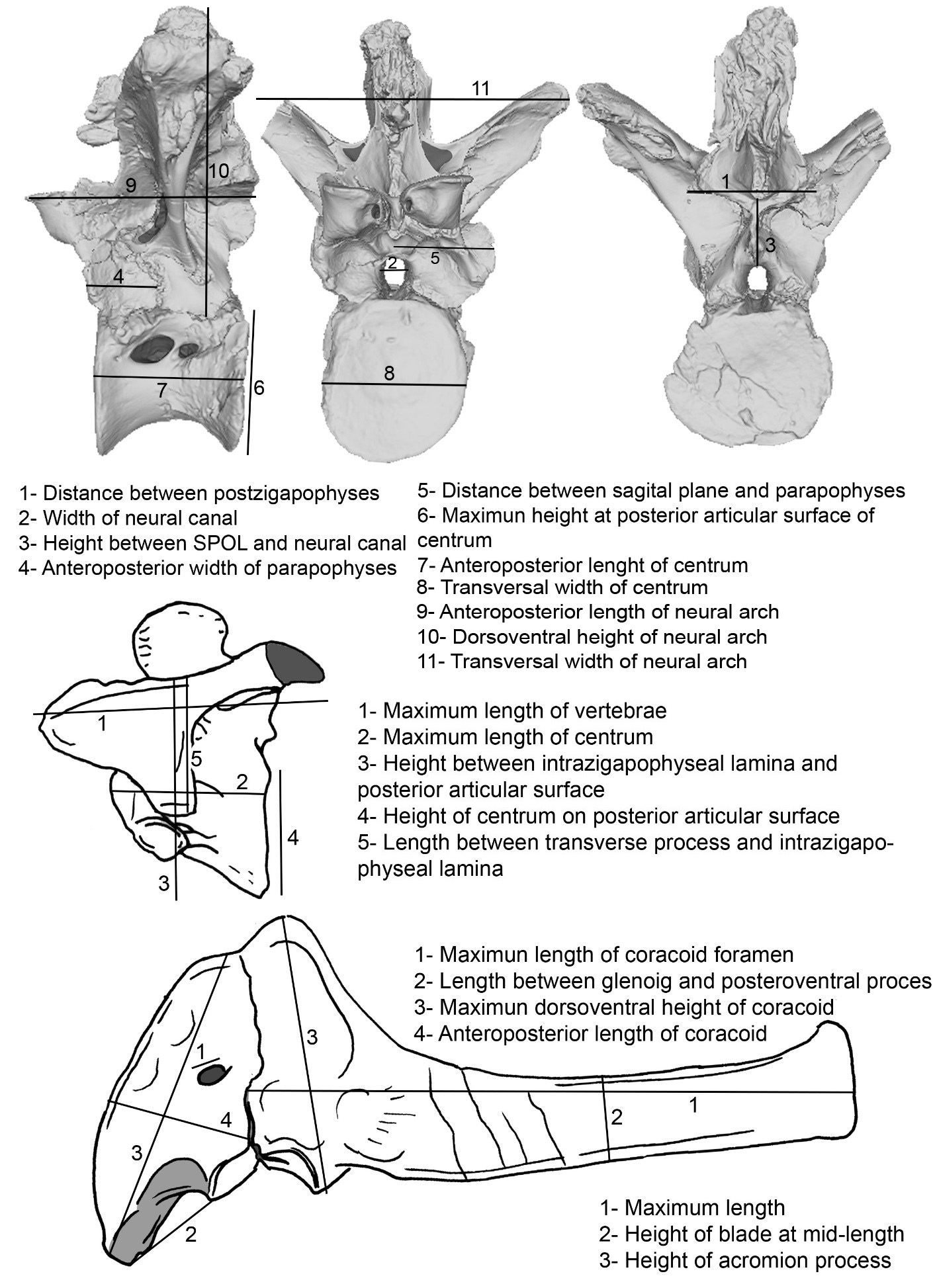


**Fig. S1:** measurements used to estimate the size of different megaraptorids

**Estimation of the transversal width of gastralia**

Entire gastral baskets are known in several tyrannosaurids^7^ and has been described for some megaraptorids such as *Aerosteon, Murusraptor* and *Megaraptor*^2,5-6^. These which gives us a wide framework to determine, orient and reconstruct those of *Maip.* In this latter taxa, the more preserved medial element has 45 cms. The morphology of this gastral rib indicates that pertains to a middle element. Nevertheless, this elements lacks almost a 10% of its shaft, so we calculate an approximate total width of 60 cms when complete (based on some complete elements of *Aerosteon, Megaraptor* and *Murusraptor*). The gastral ribs of megaraptorids are fused in most cases^5-6^ and shows, closer to mid-line, a constriction. This constriction is observed in several medial gastral elements of *Maip* (being the previously mentioned one of them). For these reasons, we estimate a total width of 120 cms when both medial elements were fused.

The lateral elements are completely preserved in *Maip*. The bigger ones has 20 cms; however, as occurs in other theropods, the lateral and medial elements articulates overlapping almost half of its transversal length^7^. In this sense, over the 120 cms previously estimated, we have to add 20 cms to the transversal width of the gastral basket (10 cms from each lateral gastral rib). The total length of the gastral basket would be approximately 140 cms (calculated closer to the middle of the chest). Compared with other megaraptorids, the biggest preserved gastral complex (two medial plus two lateral elements) of *Murusraptor* has 80 cms.

**Phylogenetic analysis**

To evaluate the phylogenetic position of *Maip macrothorax*, we use the dataset presented in Aranciaga Rolando *et al*.^8^ which includes the codifications from several previous analyses that have focused largely on megaraptorans^9-12^. To this matrix we add six new characters: dorsal vertebrae with a bifurcated lamina anterior to the transverse process and forming an accessory fossa (Ch. 352); presence of elongated articular facets on pre- and postzygapophyses of caudal vertebrae (Ch. 353); orientation of the cnemial crest of tibia (Ch. 354); presence of a dorsal curvature on the tibial shaft (Ch. 355); astragalus with one or two transverse grooves in anterior view (Ch. 356); and ascending process of astragalus reaching the entire width of the astragalar body (Ch. 357). To this data matrix we also include the basal megaraptoran *Aoniraptor libertatem*^13^, and some materials referred as Megaraptora or Megaraptoridae indet (such as LRF 100-106 and UNPSJB PV-944/958). The resulting dataset is composed of 356 morphological characters and 58 theropod taxa (56 when the taxa with less than the 15% of the skeleton are pruned) (Supplementary information I). The data matrix was analyzed using TNT version 1.5^14^. The methodology used was the traditional search function (TBR) with a swapping algorithm and random seed 1. The search includes 15000 replications and 10 trees saved per replication.

Both analyzes (with and without pruned taxa) were made under equally weighted parsimony using TNT 1.5^14-15^. A new technologies search of 1500 replicates of Wagner trees were conducted in both cases, followed by TBR branch-swapping algorithm (holding 10 trees per replication) and with one random seed. The best obtained trees were submitted to a final round of TBR branch swapping. The branches with a maximum possible length of zero among any of the recovered most parsimonious trees were collapsed. Characters 1, 3, 5, 12, 14, 16, 26, 68, 105, 147, 154, 157, 159, 168, 170, 178, 180, 193, 194, 204, 207, 216, 234, 240, 258, 266, 270, 309, 315, 316, 317, 322, 323, 324, 338; were treated as additive (=ordered). Decay indices (=Bremer support) were obtained (Bremer 1988, 1994), as well as a bootstrap resampling analysis. The consistency and retention indexes of the analyses were calculated.

***Character list modified from Aranciaga Rolando et al.,^8^***

Multistate characters were considered as ordered (= additive) only when indicated. Character 251 of Apesteguía et al.,^11^ was removed from the data matrix because it is redundant with other character. Also this data matrix includes characters 288-291 from an analysis in progress of a sacrum of Brazil^16^. Characters 3,4,8,9,12-19,21-32,34-40,85,86,88-92,94,100,103,141-147, 196 of *Megaraptor* were scored based on the new material descripted in Porfiri et al.,^2^. Characters 7, 9, 88, 89, 90, 243 and 271 of *Australovenator* were rescored based on the new dentary of White et al.,^17^.

1. Teeth, enamel wrinkles near the denticles: absent (0); present (1)^1^.
2. Teeth, denticles density: mesial and distal denticles subequal (0); more mesial denticles than distal denticles (1); mesial denticles absent (2)^1^. The isolated shed tooth described with the holotype of *Aerosteon* is considered here as belonging to an abelisaurid theropod.
3. Teeth, premaxillary teeth subequal to rostral maxillary teeth in size (0) or significantly smaller (1)^1^.
4. Teeth, position of mesial carina in premaxillary teeth: offset mesial to distal carina on all teeth (teeth ziphodont) (0); rotated distally on premaxillary teeth 1 and 2 (anterior teeth “D-shaped” and posterior teeth ziphodont) (1); rotated distally on all teeth (2)^1^
5. Teeth, curvature of posterior crowns in the premaxillary teeth: recurved (0); straight (1)^1^
6. Teeth, median vertical ridge on lingual surface of premaxillary teeth: absent (0); present as subtle structure in anterior (mesial) premaxillary teeth (1); present as pronounced structure in all premaxillary teeth (2)^1^
7. Teeth, contour of maxillary and dentary crowns in cross-section: elliptical (labiolingually compressed) (0); subcircular with serrated carinae (1); subcircular without serrated carinae (2).^1^
8. Teeth, maxillary and dentary crown curvature: mesial margin strongly curved and distal margin slightly curved or straight (0); mesial and distal margins strongly curved, with the apex positioned well distally from the distal margin (1); mesial and distal margins nearly straight (2)^1^
9. Maxilla and dentary, alveolar contour: ovoidal or subcircular (0); subrectangular (1)^1^
10. Premaxilla, shape of anterior margin: smoothly curved (0); discrete inflection point between nearly vertical anterior region and more horizontal dorsal region (1)^1^
11. Premaxilla, deep foramen or fossa on the lateral surface of the base of the nasal process, within the anteroventral corner of the narial fossa: absent (0); present (1).^1^
12. Premaxilla, form of narial fossa ventral to external naris: shallowly excavated (0); deeply excavated, anterior margin invaginated as a deep groove (1)^1^
13. Premaxilla, orientation of tooth row: strongly parasagitally (anteroposteriorly) (0); first two teeth oriented mediolaterally and third and fourth teeth oriented parasagitally (1); entire tooth row oriented mediolaterally and all teeth visible in anterior view (2)^1^
14. External naris, less (0) or greater than (1) 20% of skull length^1^
15. Premaxilla, number of teeth: 4 (0); 3 (1); 5 (2); more than 5 (3); absent (4)^1^
16. Orbit, contour: rounded (0); dorsoventrally elongated (1)^1^
17. Antorbital fossa, openings within it: absent (0); one antrum/opening (1); two antrum/opening (2); more than three openings (3)^1^
18. Maxilla, orientation of promaxillary foramen: absent or laterally oriented (0); posteriorly oriented (1)^1^
19. Maxilla, position of the maxillary fenestra with respect to the anterior corner of antorbital fossa: maxillary fenestra absent or anterior margin ends posterior to the anterior margin of the antorbital fossa (0); ends at the anterior margin of antorbital fossa (1)^1^
20. Maxilla, medial wall of the promaxillary recess: absent or reduced (0); continuous (1); fenestrated, medially opened (2)^1^
21. Maxilla, morphology of the lateral lamina of ascending process: absent or reduced (0); developed as a wide shelf that covers the anterior margin of antorbital fossa (1)^1^
22. Maxilla, orientation of its contact with jugal: horizontal (0); approximately 20º oriented ventrally (1)^1^
23. Maxilla, anterior portion of alveolar margin: subhorizontal (0); dorsally oriented (1)^1^
24. Maxilla, ornamentation: absent or restricted to anterior and lateral margins of the dental series (0); present, and covering all lateral surface of maxilla principally formed by neurovascular grooves (1)^1^
25. Maxilla, posterior paradental plates: separated (0); fused (1)^1^
26. Maxilla, dorsoventral depth of anterior paradental plates: less than twice anteroposterior length (0); more than twice (1)^1^
27. Premaxilla, depth/length ratio under external nares: 0.5-1.25 (0); <0.5 (1); >1.25 (2)^1^
28. Premaxilla, orientation of ventral edge of anterior margin: subvertical (0-9º) (0); slightly posterodorsally oriented (10-20º)^1^
29. Maxilla, orientation of the pair of maxillae when viewed dorsally to each other: posteriorly divergent (0); subparallel (1)^1^
30. Maxilla, anterior ramus: confluent with ascending process (0); slightly convex and poorly extended anteriorly, being shorter than tall (1); subrectangular and well extended anteriorly, being as tall as long as or longer than tall (2)^1^
31. Maxilla, anteroposterior extension of antorbital fossa on the ascending ramus of maxilla: being at least 10-25% total anteroposterior length of antorbital fenestra (0); more than 40% (1)^1^
32. Maxilla, development of ventral antorbital fossa, along maxillary body: poorly developed on the ventral margin of antorbital fenestra, the lateral margin of the fenestra at the same level than its medial margin (0); well developed, the lateral margin of the fenestra located ventrally with respect to the medial one. This fossa is clearly observed in lateral view (1)^1^
33. Nasals, fused on midline: absent (0); present (1)^1^
34. Nasals, ornamentation: weak or absent (0); homogenous (1); strong rugosities and knob-like projections in some portions of the bone (2); high median crest starting directly posteriorly to the external nares (3)^1^
35. Nasals, contour in dorsal view: posteriorly expanded (0); medial and lateral margins subparallel (1)^1^
36. Nasals, posterior margin: medial projection extending more than the lateral one (0); lateral projection extending more than the medial one (1)^1^
37. Nasals, pneumatization: absent (0) ; present (1)^1^
38. Nasals, lateral crests: absent (0); present (1) ^1^
39. Prefrontal: well-developed (0); reduced or absent (1) ^1^
40. Prefrontal, co-ossified with lacrimal: absent (0); present (1) ^1^
41. Frontal, morphology of anteromedial corner of supratemporal fossa: dorsally opened (0); covered by a frontoparietal shelf (1)^1^
42. Frontals: separated (0); fused (1) ^1^
43. Frontal and parietals: separated (0); fused (1) ^1^
44. Lacrimal-postorbital, contact: absent, separated by frontal (0); present (1) ^1^
45. Postorbital-squamosal, articulation: wide and flat contact (0); joined suture, transversely wide (1); spiral suture, with a wide medial process (2) ^1^
46. Postorbital, suborbital flange: absent (0); present (1) ^1^
47. Postorbital, ventral extension of ventral process: at the same level of the ventral margin of the orbit (0); dorsal to the ventral margin of the orbit (1) ^1^
48. Postorbital, ventral process in cross section: triangular (0); “U”-shaped (1) ^1^
49. Postorbital, distal extreme of jugal process: undivided (0); divided by a vertical sulcus (1) ^1^
50. Postorbital, dorsal protuberance: absent or poorly developed (0); bulbous protuberance (1)^1^
51. Postorbital, anterior process: dorsally oriented (0); anteriorly oriented (1) ^1^
52. Postorbital, medial contact for laterosphenoid: deep and cup-shaped (0); almost flat (1) ^1^
53. Postorbital, edge for the articulation with jugal on its ventral process visible in lateral view: absent (0); present (1) ^1^
54. Lacrimal, pneumatic recess: absent (0); present as a large recess (1); present as a small foramen (2) ^1^
55. Lacrimal, horn: absent (0); present as a small rugosity or conical horn (1); present as a robust rugose ridge (2)^1^
56. Jugal, pneumaticity: absent (0); pierced by a foramen on the posterior margin of antorbital fossa (1) ^1^
57. Jugal, with a foramen on its medial surface: absent (0); present (1) ^1^
58. Jugal, quadratojugal processes: subequal (0); dorsal process shorter than ventral one (1) ^1^
59. Jugal, depth of the postorbital process with respect to the jugal body: lower than the jugal body (0); well-developed (1) ^1^
60. Jugal, depth of the postorbital process with respect to the anteroposterior length at base of this process: wide base, depth of the process less than twice its base width (0); narrow base, depth of the process more than twice its base width (1) ^1^
61. Jugal, contour of anteroventral margin in lateral view: curved or sigmoidal (0); straight (1) ^1^
62. Jugal, morphology of antorbital fossa: absent or poorly developed (0); well developed as a deep depression (1) ^1^
63. Squamosal, edge of ventral process in the infratemporal fenestra: absent (0); present (1) ^1^
64. Squamosal, ventral process: longer than the posterior one (0); subequal to the posterior one (1) ^1^
65. Quadratojugal, posteroventral process: present (0); absent, quadratojugal L-shaped (1) ^1^
66. Quadrate foramen: absent (0); present, located between the quadrate and quadratojugal (1); present, surrounded by the quadrate (2) ^1^
67. Quadrate, pneumaticity: absent (0); present, deep recess on the anterior surface where the pterygoid wing and condyles meet (1) ^1^
68. Braincase, angle between the main axis of the occipital condyle and the transverse section of basal tubera: aproximately 90º (0); less than 75º, occipital surface sloping posteroventrally (1) ^1^
69. Braincase, pneumatization: apneumatic (0); moderately pneumatic (1); highly pneumatic (2) ^1^
70. Basal tubera, composition: formed by basioccipital and basisphenoid, not subdivided (basioccipital located posteriorly and basisphenoid located anteriorly) (0); subdividided by lateral grooves (basioccipital located medially, basiesfenoid located laterally) (1) ^1^
71. Basal tubera, transverse width with respect to the occipital condyle width: equal or greater (0); lower (1) ^1^
72. Paroccipital processes, orientation: laterally or ventrolaterally oriented, with its distal extreme not surpassing completele the ventral margin of the foramen magnum (0); strongly tilted ventrally, with its distal end entirely located under the ventral level of the foramen magnum (1) ^1^
73. Laterosphenoid, location of the opening for V cranial nerve: anterior or ventral to the nuchal crest (0); posterior to the nuchal crest (1) ^1^
74. Interorbital septum: absent (0); present (1) ^1^
75. Basicranium, opening of the VI cranial nerve divided by a medial crest: present (0); absent (1)^1^
76. Basisphenoid, neck of the occipital condyle invaded by a pair of pneumatic cavities: absent (0); present (1) ^1^
77. Occipital condyle, contour: subesferical (0); dorsoventrally compressed (1) ^1^
78. Basisphenoid, fossa deep and funnel-shaped: absent (0); present (1) ^1^
79. Parietal, posterodorsal projection: absent or very low (0); well-developed (1)^1^
80. Parietal, sagittal crest: absent (0); present (1) ^1^
81. Supraoccipital, dorsal expansion width: less or near twice the width of foramen magnum (0); at least twice the width of foramen magnum (1) ^1^
82. Palatines, pneumatic recess: absent (0); present as a well developed fossa, pierced by a foramen (1) ^1^
83. Palatines, medial contact: absent (0); present (1) ^1^
84. Ectopterigoids, pneumatic recess: absent (0); shallow ventral depression (1); deep and elongate depression (2); deep and subcircular depression (3) ^1^
85. External mandibular fenestra, size: more than 10% total mandibular length (0); less than 10% total mandibular length (1) ^1^
86. Articular, retroarticular process: long, narrow and rod-shaped (0); wide, with a posterior groove (1) ^1^
87. Surangular, dorsoventral depth: less than half the maximum width of the mandible above the mandibular fenestra (0); more than half the maximum width of the mandible above the mandibular fenestra (1) ^1^
88. Dentary, rostral end: rounded (0); subrectangular (1) ^1^
89. Dentary, anteroventral process: absent (0); present (1) ^1^
90. Dentary, anterior portion of vascular groove: straight (0); ventrally oriented (1) ^1^
91. Mandibular, arcade contour: V-shaped (0); U-shaped (1) ^1^
92. Atlas, triangular neurapophyses in lateral view: absent (0); present (1) ^1^
93. Axis, orientation of the ventral margin of the intercentrum with respect to the ventral margin of the axis: subparallel (0); dorsally oriented (1) ^1^.
94. Axis, epipophyses: small or absent (0); well-developed (1) ^1^.
95. Axis, distal extreme of neural spine: not expanded (0); expanded, forming a spine table (1) ^1^
96. Axis, ventral keel: present (0); absent (1) ^1^
97. Cervical vertebrae, anterior articular surface: slightly opisthocoelous (0); highly opisthocoelous (1); amphicoelous (2) ^1^
98. Postaxial cervical vertebrae, pleurocoels: absent (0); one pleurocoel (1); two pleurocoels, one located behind the parapophyses, the other near the cuadal margin of vertebral centrum (2); two pleurocoels, both located behind the parapophyses and separated by an anterodorsally oriented thin lamina (3) ^1^
99. Anterior and mid-cervical vertebrae, postzygadiapophyseal laminae: feebly developed and posteriorly concave (0); developed as a thick lamina and subvertically oriented, being the diapophyses extensive and subtriangular in lateral view (1) ^1^
100. Mid-cervical vertebrae, neural spines: thin, with a homogeneous anteroposterior length along all its depth (0); robust, with its base strongly anteroposteriorly wider than its distal end (1) ^1^
101. Cervical vertebrae, epipophyses: poorly developed and rounded (0); well-developed and finger-like (1) ^1^
102. Cervical vertebrae, hyposphene-hypantrum accessory articulations: absent (0); present (1) ^1^
103. Cervical vertebrae, prezygoepipophyseal laminae: absent (0); present as a ridge, separating the neural arch in a dorsal and lateral faces (1); present as a deep lamina (2) ^1^
104. Presacral vertebrae, centrum pneumatization: apneumatic (0); camerate (1); camellate (2) ^1^
105. Anterior dorsals, opithocoelous: absent (0); present (1) ^1^
106. Dorsal vertebrae, pleurocoels: absent (0); a single pleurocoel on anterior dorsals (1); single pleurocoel on all dorsals (2); two pleurocoels on all dorsals (3); a single pleurocoel on anterior dorsals and two pleurocoels on posterior ones (4)^1^
107. Posterior dorsal vertebrae centra: anteroposterior length: equal or longer than its depth (0); shorter than its depth (1) ^1^
108. Posterior dorsals, neural spines: subvertical (0); anteriorly oriented (1) ^1^
109. Sacral vertebrae, pleurocoels: absent (0); present (1) ^1^
110. Sacrum, laterally compressed and ventrally concave in lateral view: absent (0); present (1)^1^
111. Caudal vertebrae, hyposphene-hypantrum accessory articulations: absent or poorly developed, restricted to the base of the tail (0); well-developed and extended approximately along the first third of the tail (1)^1^
112. Caudal vertebrae, pleurocoels : absent (0) ; present (1) ^1^
113. Caudal vertebrae, ventral surface: flat (0); double keel (1); single keel (2) ^1^
114. Distal caudal vertebrae, prezygapophyses length: reaching at least 40% or more preceeding vertebral centrum (0); less than 40% (1) ^1^
115. Proximal caudal vertebrae, centrodiapophyseal laminae: weak (0); comparable in prominence or more developed than those of the dorsal vertebrae (1) ^1^
116. Dorsal ribs: apneumatic (0); proximally pierced by foramina (1) ^1^
117. Gastralia: no more than a pair proximally fused at midline (0); more than a pair proximally fused at midline with expanded club-shaped proximal ends (1) ^1^
118. Scapula-coracoid, deep notch between the acromial process and coracoid: absent (0); present (1) ^1^
119. Scapula, scapular blade length compared with minimum width of scapula: less than 7,5 (0); more than 7,5 (1) ^1^
120. Scapula, anterior margin of scapular blade: straight (0); strongly concave (1) ^1^
121. Scapula, distal end: expanded (0); not expanded (1), relative to proximal portion of scapula^1^
122. Scapula, acromion process: does not match any of the following descriptions (0); rectangular with its dorsal edge forming a 90º angle with the dorsal edge of the scapular blade (1); a quarter-circle in shape (2); triangular, with apex pointing away from and subparallel to scapular blade (3) ^1^
123. Coracoid, coracoid foramen: present (0); absent or extremely small (1) ^1^
124. Coracoid, caudoventral process: absent or poorly developed (0); well-developed, crescent-shaped (1) ^1^
125. Humerus, shape in lateral view: sigmoid (0); straight (1) ^1^
126. Humerus, form of distal condyles: lateral and medial condyles expanded equally (offset from shaft in anterior or posterior view is equal) (0); medial condyle expanded further medially than the lateral condyle is laterally (1) ^1^
127. Ulna, shape of the olecranon process: anteroposteriorly short and square-shaped in lateral view (0); anteroposteriorly long, being extended at the base of the coronoid process, and widely sub-triangular in lateral view (1) ^1^
128. Ulna, olecranon: wide, transversely expanded (0); transversely compressed (1) ^1^
129. Ulna, lateral tuberosity: absent or poorly developed (0); developed as a prominent crest continuous with a longitudinal ridge and forming an obtuse angle with the olecranon (1); developed as a prominent crest continuous with a longitudinal ridge and forming an right angle with the olecranon (2) ^1^
130. Forelimb, ratio of radius length/humerus length: radius more than 50% humeral length (0); radius less than 50% humeral length (1) ^1^
131. Forelimb, hand length/humerus+radius length: <2/3 (0); >2/3 (1) ^1^
132. Metacarpal II, at least half of proximal end closely appressed to metacarpal III: absent (0); present (1) ^1^
133. Metacarpal II, mediolateral width at midpoint: equal to or narrower than (0); or more robust than (1) metacarpal I. ^1^
134. Metacarpal II, maximum length: equal to or less than 3 times its distal transverse width (0); more than 3 times its distal transverse width (1) ^1^
135. Metacarpal III, length: >0.75 length of metacarpal II (0); <0.75 length of metacarpal II (1) ^1^
136. Metacarpal III, transverse proximal expansion: prominent, much wider than distal articular surface (0); incipient or absent (1) (Brusatte and Sereno, 2008).
137. Metacarpal IV: present (0); absent (1) ^1^
138. Manual phalanx I-1, ventral groove: absent (0); present (1) ^1^
139. Manual phalanx I-1, proximal end: subtriangular in proximal view, with the dorsal portion transversely narrower than the ventral one (0); subquadrangular in proximal view, with the dorsal portion transversely wider than the ventral one (1) ^1^
140. Manual phalanx I-1, shape: short (0); elongate (its lenght is three times its proximal depth) (1) ^1^
141. Manual ungual I-2, transverse compression: absent; sub-rectangular to sub-triangular in proximal view (0); strongly transversely compressed, being oval with a distinct dorsoventral axis in proximal view (1) ^1^
142. Manual phalanx III-3, length: subequal to or shorter than combined lengths of phalanges III-1 and III-2 (0); longer than their combined lengths (1) ^1^
143. Manual unguals, flexor tubercle, form: large, robust, rugose, conical structure (0); reduced to a small convexity (1) ^1^
144. Manual unguals, lateral grooves of manual ungual of digit I: nearly symmetrical (0); asymmetrical, with the internal furrow occupying a higher position than the external (1)^1^
145. Manual unguals, ventral margin of manual unguals I and II: flat or slightly convex (0); a well-developed ridge that connects the flexor tubercle (1) ^1^
146. Ilium, anteroposterior length of pubic peduncle equals or surpasses two times its transverse width: absent (0); present (1) ^1^
147. Ilium, lateral wall of brevis fossa: ventrally extended (0); poorly extended ventrally (1) ^1^
148. Ilium, fossa cuppedicus: absent (0); present (1); present and bounded dorsomedially by a prominent shelf (2)^1^ ORDERED.
149. Ilium, anteroposterior length compared to length of femur: 70-95% (0); 95-115% (1). ^1^
150. Ilium, dorsal margin of blade, position relative to sacral neural spines: separated by a gap (0); lies against neural spines and opposing iliac blades may make contact above neural spines in some individuals (1) ^1^
151. Ilium, supraacetabular crest, maximum lateral projection relative to ischial peduncle: significantly greater (0); subequal (1). ^1^
152. Ilium, postacetabular process, form of posterior margin: tapering, posterior margin convex (0); squared-off, posterior margin vertical (1) ^1^
153. Ilium, ratio of anteroposterior length to dorsoventral depth above acetabulum: equal or greater than 2.8, ilium is long and low (0); less than 2.8, ilium is subovoid shape (1) ^1^
154. Ilium, exposition of fossa brevis in lateral view: widely exposed and inviding the base of the ischiadic peduncle (0); mostly hidden by the brevis shelf and not inviding the base of the ischiadic peduncle (1) ^1^
155. Pubis, obturator opening: foramen (0); incipient notch (1); wide and well-developed opening (2) ^1^
156. Pubis, shaft in lateral view: straight (0); anteriorly convex (1); anteriorly concave (2) (Rauhut, 2003). In *Neovenator* the “the curvature of the shaft in lateral view cannot be determined due to crushing”. ^1^
157. Pubis, orientation: propubic (0); vertical or posteriorly oriented (1) ^1^
158. Pubis, pubic tubercle: absent (0); present as a convexity on the anterior margin of the pubis (1); present as a rugose flange that is discretely offset from the anterior margin of the pubis and is bordered posteriorly by heavy rugosities on the lateral surface on the obturator region of the pubis (2) ^1^
159. Pubis, pubic symphisis in anterior view: continuous up to the distal end of the bone (0); interrumpted distally by a large median fenestra (1) ^1^
160. Pubis, length of pubic boot: ≤40% length of the bone (0); ≥40% length of the bone (1); ≥60% length of the bone (2) ^1^ ORDERED.
161. Pubis, pubic boot, position of anterior process relative to posterior process: displaced dorsally, resulting in a highly convex ventral margin of the boot (0); placed at the same level, ventral margin of the boot essentially straight (1) ^1^
162. Pubis, anterior projection of pubic boot compared with posterior one: well-expanded (0); small or absent (1) ^1^
163. Ischium, obturator opening: absent (0); foramen (1); notch (2) ^1^
164. Ischium, ventral groove separating the obturator process and ischiadic diaphysis: absent (0); present (1) ^1^
165. Ischium, lenght compared with pubis lenght: >2/3 (0); <2/3 (1) ^1^
166. Ischium, surface for articulation with the ilium: flat (0); deeply concave (1) ^1^
167. Ischium, ischial tubercle ventral to iliac peduncle: absent or present as a groove (0); present as a convex bulge on the posterior surface of the ischium (1); present as a rugose, ovoid or triangular flange whose lateral surface is depressed relative to the remainder of the ischium (2) ^1^
168. Ischium, symphysis: unexpanded (0); expanded as apron (1) ^1^
169. Ischium distal expansion: absent (0); present (1); well-developed, forming a boot (2) ^1^ ORDERED.
170. Ischium, presence of a flange posteriorly oriented in the iliac peduncle: absent (0); present (1) ^1^
171. Femur, femoral head angle with respect to the shaft: less than 90° (0); perpendicular (1); more than 90° (2) ^1^
172. Femur, femoral head orientation: anteromedially oriented (0); medially oriented (1) ^1^
173. Femur, greater trochanter prominent, proximally projected: absent (0); present (1) ^1^
174. Femur, lateral border of the femoral head in proximal view: rounded, equal or less than half of the maximum anteroposteiror length of the femoral head (0); strongly tapering (1); squared, more than half of the maximum anteroposteiror length of the femoral head (2) ^1^
175. Femur, fossa on the medial surface of the head, lateral to the trochanteric fossa, form: absent or shallow (0); deep (1) ^1^
176. Femur, anterior trochanter with an anterior proyection at mid-length: present (0); absent, anterior margin of the anterior trochanter straight or gently convex in lateral or medial view (1) ^1^
177. Femur, lateral condyle, shape in distal view: circular or ovoid (0); ovoid, but with an anterior bulge that is slightly separated from the remainder of the condyle (1) ^1^
178. Femur, tibiofibular crest: sub-triangular or sub-rectangular (0); kidney-shaped (1) ^1^
179. Femur, anterior trochanter: distally positioned, under the ventral level of the femoral head (0); more proximally positioned, but distal to the greater trochanter (1); at the same level or proximal to the greater trochanter (2) ^1^ORDERED.
180. Femur, fourth trochanter: robust (0); reduced or absent (1) ^1^
181. Femur, extensor groove: absent (0); wide and shallow (1); narrow and deep (2) ^1^
182. Femur, ridge for cruciate ligaments: absent (0); present (1) ^1^
183. Femur, tibiofibular fossa: wide, more than 90° between the lateral margin of tibiofibular crest and posterior margin of lateral condyle (0); narrow, less than 90° between the lateral margin of tibiofibular crest and posterior margin of lateral condyle (1) ^1^
184. Femur, lateral condyle: does not project further distally than medial condyle (0); projects distinctly further than medial condyle and distal surface of medial condyle is gently flattened in comparison (1) ^1^
185. Femur, mediodistal crest: absent or poorly developed (0); extended, representing more than a quarter of femoral length (1) ^1^
186. Tibia, maximum length: equal to or less than 12 times the anteroposterior width at mid-length (0); more than 12 times the anteroposterior width at mid-length (1) ^1^
187. Tibia, form of medial malleolus: oriented distally and medial surface smooth (0); oriented distally and distinct 'shoulder' present in outline of medial surface in posterior view (1); oriented almost medially, 'shoulder' absent (2) ^1^
188. Tibia, median prominence in the anterior surface of the distal end: absent, anterior margin straight or gengly concave in distal view (0); present (1) ^1^
189. Tibia, posteromedial notch separating lateral condyle from tibial shaft in proximal view: absent or poorly developed (0); developed as a deep notch (1) ^1^
190. Tibia, medial expansion of distal medial malleolus: slightly expanded (0); expanded 40% or more than tibial mid-shaft width (1) ^1^
191. Tibia, expansion of lateral malleolus with respect to medial malleolus: at the same level or slightly extended distally (0); extended distally beyond the medial malleolous more than 5% of tibial total length (1) ^1^
192. Fibula, length compared with femoral length: subequal (0); shorter (ca. 70 %) (1)^1^
193. Fibula, deep groove on medial side of proximal end: absent (0); present, but covering less than two-thirds of the width of the fibula (1); present and wide, covering more than two-thirds the width of the fibula (2); present and opening posteromedially (3) ^1^
194. Fibula, ratio of anteroposterior width of distal end to minimum shaft width: 2.3 or greater (0); 1.9–2.1 (1); less than 1.7 (2) ^1^ ORDERED.
195. Astragalus, ascending process height: less than 0.4 times the width of the astragalar body (0); equal to or less than 0.5 times the width of the astragalar body (1); more than 0.5 times the width of the astragalar body (2)^1^ ORDERED.
196. Astragalus, transverse width of ascending process: not occupying total width of anterior surface of distal tibia (0); occupying total width of anterior surface of distal tibia (1)^1^
197. Astragalus, distinct anterior development of the lateral condyle of the astragalar body: absent, lateral half of the anterior margin of the astragalar body slightly concave in distal view (0); present, strong inflexion in the anterior margin of the astragalar body in distal view (1) ^1^
198. Astragalus, with a prominent posterolateral extension: absent (0); present (1) ^1^
199. Astragalus, fibular facet: well-developed and proximally oriented (0); reduced and laterally oriented or absent (0) ^1^
200. Calcaneum, transverse development: moderately wide (0); strongly compressed, being a disc-like element (1) ^1^
201. Calcaneum, shape in lateral or medial view: roughly symmetric, with wide angles in the posterior border (0); strongly assymetric, with a right angle in the posterior border (1) ^1^
202. Pes, shafts of metatarsals: not appressed (0) or appressed (1) ^1^
203. Metatarsal III, distal articular surface in anterior view: poorly extended dorsally and slightly excavated (0); well-developed ginglymoid, very extended proximally (1) ^1^
204. Metatarsal III, extensor fossa of the distal end: superficial (0); deeply excavated with a very wide and crescent-shaped flexor fossa (1) ^1^
205. Metatarsal IV, distal end, ratio between anteroposterior long axis (measured from midpoint of condyles posteriorly to anterior surface of bone) and mediolateral width (measured at midpoint): greater than 1.40, distal surface is elongate anteroposteriorly (0); between 1.40 and 1.20 (1); less than 1.20, distal surface nearly square-shaped with nearly flat anterior surface (2)^1^ ORDERED.
206. Quadratojugal, anterior process: equal or longer than the dorsal process (0); shorter than the dorsal process (1)^1^
207. Pubis, distal end: transversely broad, expanded from the transverse width of the shaft (0); transversely flattened (1)^1^
208. Tibia, facet for the reception of the ascending process of the astragalus at the distal end: distinct step running obliquely from mediodistal to lateroproximal (0); very slight edge without well-defined borders (1); anterior side of tibia more or less flat (2) ^1^
209. Metatarsal III, ratio of length to minimum width: short and stout, ratio smaller than 10 (0); slender, ratio larger than 10 (1)^1^
210. Chevrons, proximal elements: rod-like (0); boat shaped (1)^1^
211. Astragalus, ascending process: nearly confluent with astragalar distal condyles (0); offset from distal condyles by a pronounced groove (1)^1^
212. Metatarsal III, anterior view: exposed surface wider or subequal to metatarsals II and IV (0); exposed surface clearly smaller than metatarsals II and IV (1); proximal half of metatarsal III strongly decrease in width towards the proximal end, pinched (2) ^1^
213. Cervical vertebrae, anterior articular surface: subcircular or ellipsoidal (0); kidney-shaped (1) ^1^
214. Sacrum, ventral surface: keeled (0); flattened, usually with a ventral sulcus (1)^1^
215. Carpus, semilunate carpal: absent (0); present (1)^1^
216. Manual unguals, proximodorsal lip: absent (0); present (1) ^1^
217. Premaxilla, with a long and tapering subnarial ramus: absent (0); present, shorter than premaxillary main body (1); present, longer than premaxillary main body (2) ^1^ ORDERED.
218. Forelimb, distal end of radius and ulna adhering tightly distally: absent (0); present (1) ^1^
219. Dorsal vertebrae, neural spines shape: subrectangular or cuadrangular (0); fan-shaped (1) ^1^
220. Digit 1 wider than the radius: absent (0); present (1) ^1^
221. Tibia, lateral condyle position in the proximal end: at the posterior rear of the tibia, its posterior margin located at the same than the posterior margin of the medial condyle (0); being its posterior margin well posteriorly to the level of the posterior edge of the medial condyle (1) ^1^
222. Fibula, shaft distal to the *iliofibularis* tubercle narrows abruptly: absent (0); present (1) ^1^
223. Hindlimb, tibia/femur ratio: tibia shorter than femur (0); tibia longer than femur (1) ^1^
224. Posterior dorsal vertebrae, with a lateral process on postzygapophyses: absent (0); present (1) ^1^
225. Caudal vertebrae, number: more than 50 (0); less than 50 (1) ^1^
226. Ilium, anterior margin of preacetabular blade: straight or convex (0); with a rostrodorsal notch (1) ^1^
227. Squamosal-frontal contact: distant (0); in near touch (1) ^1^
228. Ulna, shaft: straight (0); caudally bowed (1)^1^
229. Nasal, dorsal extent of antorbital fossa: dorsal rim of antorbital fossa below nasal suture, or formed by this suture (0); antorbital fossa extending onto the lateral surface of the nasals (1)^1^
230. Lacrimal, suborbital process: absent (0); present (1)^1^
231. Postorbital, lateral surface of anterior process: thin and unornamented or weakly rugose (0); dorsoventrally thickened into a laterally projecting and highly rugose platform (1) ^1^
232. Chevrons, mid-caudal elements: rod-like or only slightly expanded ventrally (0); L-shaped (1) ^1^
233. Humerus, length relative to the femur: 50-70% (0); 50-30% (1); 20-30% (2) ^1^
234. Humerus, anterior surface of bone adjacent to ulnar condyle: smooth or gently depressed (0); bears well-defined fossa (1) ^1^
235. Axis, diapophyses: moderate (0); reduced/absent (1) ^1^
236. Axis, parapophyses: moderate/prominent (0), reduced/absent (1) ^1^
237. Manus, metacarpal III to metacarpal II width ratio: more than 0.55 (0); less than 0.35 (1) ^1^
238. Lacrimal, recess: single opening (0); multiple openings (1)^1^
239. Palatine, jugal process: tapered (0); expanded (1) ^1^
240. Coracoid, tubercle (= acrocoracoid process or biceps tubercle): absent or poorly developed (0); conspicuous and well developed as a tuber (1); developed as an obliquely oriented ridge (2) ^1^
241. Humerus, deltopectoral crest length to humeral length ratio: less than 0.4 (0); 0.43–0.49 (1); more than 0.52 (2) ^1^ ORDERED.
242. Vertical ridge on iliac blade above acetabulum: absent (0); developed as a low swollen ridge with associated foramina (1); present as a well-developed ridge (2) ^1^
243. Dentary, number of Meckelian foramina: one (0); two (1) ^1^
244. Dorsal vertebrae, hyposphene: laminae diverge ventrolaterally to form a triangular shape in posterior view (0); laminae vertical forming a sheet-like hyposphene (1) ^1^
245. Ilium, lateral surface of ilium with large external foramina and internal pneumatic spaces: absent (0); present (1) ^1^
246. Tibia, lateral condyle of proximal end curves ventrally as a pointed process: absent (0); present (1) ^1^
247. Coracoid, ventral portion to the glenoid: flat or slightly concave (0); exhibiting a prominent fossa (1) ^1^
248. Maxilla, size of ascending ramus, anteroposterior chord directly above maxillary fenestra compared to dorsoventral depth of maxilla below anterior edge of antorbital fenestra: greater than 1.75 times (ascending ramus large) (0); less than 1.60 times (ascending ramus small) (1)^1^
249. Maxilla, swollen rim separating antorbital fossa and subcutaneous surface: present (0); absent (1) ^1^

250 Maxilla, foramen (probably promaxillary foramen) within the extreme anteroventral corner of antorbital fossa: absent (0); present (1) ^1^

251. Maxilla, foramen (probably promaxillary foramen) within the extreme anteroventral corner of antorbital fossa: absent (0); present (1) ^1^

252. Maxilla, posterior region of the main body (portion including the final 3-5 teeth and anterior to the jugal process), shape: maintains a relatively constant dorsoventral depth (0); tapers in depth posteriorly (1) ^1^

253. Skull, area of antorbital fenestra: greater than that of orbit (0); less than that of orbit (1) ^1^

254. Nasal, medial processes of frontal articulation, shape: processes absent (0); lanceolate (1); tapered (2) ^1^

255. Lacrimal, ventral ramus: broadly triangular, articular end nearly twice as wide anteroposteriorly as lacrimal body at juncture between anterior and ventral ramus (0); bar- or strut-like, roughly same width anteroposteriorly throughout ventral ramus (1) ^1^

256. Jugal, suborbital process: short and dorsoventrally stout (0) or elongate and dorsoventrally narrow (1) ^1^

257. Jugal, postorbital ramus, orientation relative to ventral margin of jugal: approximately perpendicular (0); posterodorsal (obtuse angle between the long axis of the process and the ventral margin) (1) ^1^

258. Jugal, cornual process: absent (0); present (1); present and distinctive (mediolaterally wide and heavily rugose (2). ^1^ ORDERED.

259. Prefrontal, contacts nasal: yes (0); no, excluded by frontal-lacrimal contact (1) ^1^

260. Frontal, size of single frontal: ratio of anteroposterior length of exposed portion on skull roof to mediolateral width at midpoint: greater than 2.5 (0); less than 2.0 (1) ^1^

261. Postorbital, anterior ramus, form: short, long axis is equal or less than half the length of the ventral ramus (0); long, long axis is greater than 55% of the length of the ventral ramus (1) ^1^

262. Postorbital, ventral ramus, anteroposterior width at midpoint: approximately the same width as (0) or substantially wider than (1) ventral ramus of the lacrimal. ^1^

263. Postorbital, squamosal ramus, extent: reaches or extends posterior to (0) or terminates anterior to (1) posterior margin of lateral temporal fenestra. ^1^

264. Quadratojugal and squamosal, constriction of lateral temporal fenestra: absent, anterior margins of both bones are approximately vertical (0); present, convex kink along the suture between the two bones that projects into the fenestra, constricting it to approximately one half of its maximum anteroposterior length (1); present, dorsal region of quadratojugal expanded anteroposteriorly relative to the remainder of the bone, constricting fenestra to at least one half of its maximum anteroposterior length (2)^1^

265. Parietal, skull table between supratemporal fossae, width: wide, more than 30% of the mediolateral width of the fossa (0); broad, 10-30% of the mediolateral width of the fossa (1); extremely reduced, sagittal crest or crests pinched between opposing fossae (2)^1^

266. Supraoccipital, contribution to dorsal rim of foramen magnum: forms entire rim (0); makes limited contribution to rim via triangular ventral process (1); completely excluded from rim (2) ^1^ ORDERED.

267. Exoccipital-opisthotic, crista tuberalis (= metotic strut), extent in posterior view: limited, mediolateral width across opposing cristae less than one half the dorsoventral depth of the braincase from the dorsal tip of the supraoccipital to the ventral tip of the basal tubera (0); extensive, width greater than one half the braincase depth (1) ^1^

268. Basisphenoid, basisphenoid recess, orientation of central axis: vertical, recess obscured in posterior view (0); posteroventral, recess partially or widely visible in posterior view (1). ^1^

269. Basisphenoid, with pronounced muscle scars flanking basisphenoid recess: no (0), yes (1). ^1^

270. Dentary, anterior alveoli, size in comparison to alveoli in middle of tooth row: approximately same size (0); first alveolus substantially smaller (1); first two alveoli substantially smaller (2)^1^ ORDERED.

271. Teeth, blood grooves: horizontal (0); oblique (1) ^1^

272. External mandibular fenestra, dorsoventral depth relative to depth of mandible at midpoint of fenestra: greater (0) or less than (1) 25%^1^

273. Surangular, surangular shelf, orientation relative to the long axis of the lower jaw: anterodorsal (0); anteroventral (1); straight anteroposteriorly (2) ^1^

274. Articular, mediolateral width of jaw muscle attachment site: less than (0) or equal to or greater than (1) width of glenoid for articulation with quadrate^1^

275. Articular, smooth non-articular region between glenoid and attachment site for depressor mandibular muscles: present (0); absent (1)^1^

276. Axis, “epineurapohyseal” processes on neural spine, absent (0) or present (1) ^1^

277. Cervical vertebrae, hypapophysis on anterior region of ventral surface: absent (0); present (1) ^1^

278. Cervical vertebrae, orientation of posterior centrodiapophyseal lamina in anterior-middle cervicals: projects posteroventrally, infrapostzygapophyseal fossa located primarily posterior to lamina (0); nearly horizontal, fossa located primarily dorsal to lamina (1) ^1^

279. Cervical vertebrae, anterior and middle cervical vertebra centra: anterior and posterior articular surfaces at the same horizontal level (0); posterior articular surface strongly ventrally offset from the anterior one (1) ^1^

280. Cervical vertebrae, anterior and middle cervical prezygapohyses: most with a pointing anterior margin (0); most with a rounded anterior margin (1) ^1^

281. Dorsal vertebrae, posterior dorsal neural spines: ≥1.5×taller than long (0) or height <1.5×length (1) ^1^

282. Sacrum, position of ventral margin of posterior articular face of sacral vertebra five in lateral view: at same level as (0) or positioned ventral to (1) ventral margin of anterior articular face^1^

283. Caudal vertebrae, neural spines: simple, undivided (0); separated into anterior and posterior alae throughout much of caudal sequence (1)^1^

284. Quadrate shaft, pneumatic foramen on posterior face: absent (0) or present (1) ^1^

285. Premaxillary symphysis, ventral view: acute, V-shaped (0) or broad, U-shaped (1) ^1^

286. MC II, proximal end: narrow, less than twice as wide as midshaft (0) or expanded, twice as wide as midshaft (1) ^1^

287. Manus: more than two digits (0) or only two digits present (1) ^1^

288. Sacrum, neural spines: (0) straight; (1) strongly curved posteriorly. ^1^

289. Sacral vertebrae, height/ length ratio: (0) almost or more tall than long; (1) more long than tall^1^

290. Sacral centrums, with a constriction at mid-length: (0) absent; (1) present^1^

291. Sacrum, neural spines: (0) surpassing the dorsal margin of the ilium; (1) at the same level^1^

292. Flexor tubercle of ungual phalanxs transversally divided by a lateromedial sulcus: (0) absent; present, but sulcus is not anteroposteriorly extended (1); present, sulcus anteroposteriorly wide and forming a smooth platform (2). ^1^

293. Humerus with a deep longitudinal furrow that runs on the medial surface of the shaft, distally to the internal tuberosity: (0) absent or (1) present.^1^

294. Distal end of humerus in anterior view: (0) with poorly developed condyles, with a shallow sulcus, or (1) with well-defined condyles and much more rounded separated by a deep groove. ^1^

295. Semilunate carpal: (0) in contact with metacarpals I and II, or (1) in contact with metacarpals I, II and III. ^1^

296. Semilunate carpal with a pair of distal projections for the articulation with metacarpals: (Madsen, 1976; Currie 2000) (0) present or (1) absent. ^1^

297. Profile of semilunate carpal: (0) disc like shape without articular surfaces or (1) proximodistally deep with obvious articular surfaces^1^

298. Distal end of metacarpal I, angle between the anteroposterior axis of the bone and the surface of the distal condyles: (0) very asymmetric, less than 50 grades, (1) relatively asymmetric, between 50-70 grades, or (2) relatively symmetric, between 70-90 grades. ^1^

299. Proximal head of Metacarpal II: (0) not being embraced, parallel surfaces dorsoventrally oriented, (1) being embraced by the lateroventral margin of metacarpal I (which is laterally projected) or (2) medially expanded, embracing metacarpal I. ^1^

300. Phalanx 1-I in proximal view: (0) transversally narrow, or (1) transversally wider (for the presence of proximolateral lips. ^1^

301. First ungual reaching the length of the ulna: (0) absent, or (1) present. ^1^

302. Ventral surface of the first ungual, with a sharp longitudinal keel, lateral displaced: (0) absent, or (1) present.^1^

303. Unguals, flexor tubercle with flexor fossae: (0) absent, or (1) present. ^1^

304. The proximal articular surface of phalanx 1.II is obliquely oriented with respect to the distal articular trochlea: (0) absent or (1) present. ^1^

305. Digit II with ventromedial longitudinal ridges: (0) absent, or (1) present. ^1^

306. Third distal tarsal, size: (0) similar than TIV, or (1) notably bigger than TIV. ^1^

307. Position of distal tarsal III: (0) over the surface of metatarsus, or (1) overlapping the posterior half of metatarsus^1^

308. Metatarsal III, in anterior view: (0) subequal in length and distal width with other metatarsals, (1) longer and laterally wider distal end than the other metatarsals. ^1^

309. Margins of the shafts of metatarsals IV: (0) sinuous or concave in the entire length, (1) straight only curving in the distal end, or (2) straight in the entire length. ORDERED^1^

310. Second and third metatarsal contact: (0) by a concave-convex surface, (1) by a smooth surface, or (2) by a flat surface. ^1^

311. Distal end of third metatarsal: (0) quadrangular in distal view, (1) trapezoidal in distal view (posterior margin expanded transversally). ^1^

312. Metatarsal IV, length in anterior view: (0) subequal with metatarsal III (reach or equal the distal end), (1) significantly shorter than Metatarsal III (not reaching the distal end). ^1^

313. Proximal end of Metatarsal IV, in lateral view: (0) single or concave, or (1) with two well-developed condyles. ^1^

314. Metatarsal III with a lateral process for the contact with metatarsal IV: (0) absent, (1) present but no in an angle of 90° respect an anteroposterior axis of the bone, (2) present in an angle of 90° respect an anteroposterior axis of the bone. ^1^

315. Internal tuberosity (= ventral tubercle) of humerus direction: (0) projected ventrally; (1) projected proximally; (2) projected caudally, separate from head by deep capital incision. ORDERED^1^

316. Metacarpal IV: (0) present with phalanges, (1) present without phalanges. ORDERED^1^

317. Metacarpal I size: (0) slightly greater than one half of metacarpal II length, but less than metacarpal II length; (1) one half to one third metacarpal II length; (2) subequal or greater than to metacarpal length. ORDERED^1^

318. Proximal articulation of metacarpal III: (0) subquadrilateral; (1) triangular; (2) crescent shaped. ^1^

319. Longest digit of the manus: (0), digit II; (1) digit I. ^1^

320. First phalanx of pollex: (0) less than or subequal to length of metacarpal II; (1) greater than length of metacarpal II. ^1^

321. Pollex ungual size: (0) subequal to ungual of digits II and III in size: (1) larger than others manual unguals. ^1^

322. Manual ungual length: (0) Short; (1) moderate; (2) extremely long; ORDERED. ^1^

323. Manual ungual curvature: (0) moderate, length/height (*Sensu* Ostrom, 1969) ratio between 8 and 14.5 ; (1) extremely curved, length/height (*Sensu* Ostrom, 1969) ratio minor to 8; (2) straight, length/height (*Sensu* Ostrom, 1969) ratio major to 14,5. ORDERED^1^

324. Metatarsal III, area of the proximal surface: (0) similar in size to metatarsal II and IV; (1) clearly larger than metatarsals II and IV; (2) clearly smaller than metatarsal II and IV. ORDERED ^1^

325. Metatarsal III proximal end, slender axis in proximal view: (0) oblique to the anteroposterior axis of the bone; (1) parallel to the anteroposterior axis of the bone. ^1^

326. Metatarsal I length: (0) not reduced; (1) reduced but retains phalanges; (2) absent. ^1^

327. Metatarsal I shaft in lateral or medial views: (0) “J” shaped; (1) straight. ^1^

328. Length of the dorsal ramus of the lacrimal respect the ventral ramus: (0) longer than the ventral ramus; (1) both ramus subequal in length; (2) ventral ramus longer than the dorsal one. ^1^

329. Angle between both rami of the lacrimal: (0) acute; (1) right or (2) obtuse. ^1^

330. Lacrimal, posterior process: (0) absence; (1) presence^1^

331. Lacrimal, posterior process: (0) square, short (same height and length) and horizontal; (1) finger-like, long and upturned; (2) short, upturned and tapering; (3) long and horizontal.^1^

332. Lacrimal prominence: (0) absent; (1) triangular hornlet; (2) ridge continuous with raised surface of lateral edge of nasals^1^

333. Lacrimal dorsal ramus, width/height ratio: (0) squared ramus in section, value of ratio closer to “1”; (1) higher than wide ramus, value of ratio closer to “0”; (2) wider than high ramus, value of ratio much more than “1” (Measured at mid-length of the ramus).^1^

334. Postorbital, length of the ventral ramus: (0) longer than the anteroposterior length of the bone; (1) equal or shorter than the anteroposterior length of the bone. ^1^

335. Postorbital, sharp and long ridge that runs along the lateral margin of the bone: (0) absence; (1) presence. ^1^

336. Postorbital, articular surface for frontals: (0) taller than long; (1) notably anteroposteriorly longer than tall. ^1^

337. Postorbital, length of the articular surface for the frontals: (0) short, does not reach the mid-length of the anteroposterior length of the bone in medial view; (1) long, reaching the middle of the major length of the bone in this view. ^1^

338. Prefrontal, presence of a ventral process: (0) present; (1) absent. ORDERED^1^

339. Prefrontal forming part of the orbit in lateral view: (0) present; (1) absent. ^1^

340. Frontal length (taken only in dorsal view): (0) frontals shorter than parietals; (1) being equal or larger than the parietal length; (2) being almost two times the length of the parietals; (3) being almost four times the length of the parietals.^1^

341. Frontal lateral expansion: (0) strong and posteriorly placed expansion; (1) moderately expansion extending from the posterior corner to the middle of the bone; (2) absent; (3) strong and posterior expansion extending unto the half of the bone. ^1^

342. Frontal shape in dorsal view: (0) subtriangular; (1) subquadrangular^1^

343. Parietals, in lateral view: (0) skull table at the same level that of the nuchal crest; (1) nuchal crest above the level of the skull table (2) skull table above the nuchal crest.^1^

344. Parietals: (0) fused; (1) separated. ^1^

345. Pterygoids, shape: (0) short, stout with wide palatine process; (1) slender, palatine process small. ^1^

346. Surangular, length/high ratio: (0) height less than eight times the length; (1) more than eight times the length. ^1^

347. Prearticular, tip of the anterior process: (0) dorsoventrally thicker and dull; (1) strongly tapering and dorsoventrally thin. ^1^

348. Prearticular, delimiting the milohioid fenestra: (0) present, with a shallow concavity forming the ventral margin; (1) present, with a prominent anterior process forming the ventral margin; (2) absent. ^1^

349. Ischium, shape of the distal end in cross section: (0) round, length equal to width; (1) oval, more anteroposteriorly long than transversally wide; (2) laminar, much more anteroposteriorly long than transversally wide (almost three times). ^1^

350. Ischium distal end in lateral view, maximum width: (0) more than 2 times, (1) less than 2 times the minimum width or (2) equal in width. ^1^

351. Osseous interischiadic symphysis: (0) strongly contacted, placed in the middle of the shaft; (1) weakly contacted, symphysis placed only in the distal end. ^1^

**New characters added in this analysis:**

352. Dorsal vertebrae with a bifurcated lamina anterior to the transverse process and forming an accessory fossa: (0) absent; (1) present.

353. Proximal caudal vertebrae, shape of pre- and postzygapophyseal articular facets: round (0) and elongated (1).

354. Tibia, direction of the cnemial crest: upturned (0); anteriorly directed (1).

355. Tibia, dorsal curvature of the shaft: curved (0); shaft straight (1).

356. Astragalus, presence of transversal groove in anterior view: with two grooves (0); with only one groove (1).

357. Astragalus, width of ascending process respect the astragalar body: process does not reach the entire width of the body and forms a step-like outline in anterior view (0); process reach the entire width of the astragalar body and exhibits a continuous outline anteriorly (1).

**Sinapomorphic traits of some clade of both analyses (with and without fragmentary taxa)**

**Character supporting Coelurosauria (both analyses):**

19-1: Maxilla, position of the maxillary fenestra with respect to the anterior corner of antorbital fossa: maxillary fenestra absent or anterior margin ends posterior to the anterior margin of the antorbital fossa (0); ends at the anterior margin of antorbital fossa (1).

51-0: Postorbital, anterior process: dorsally oriented (0); anteriorly oriented (1).

55-0: Lacrimal, horn: absent (0); present as a small rugosity or conical horn (1); present as a robust rugose ridge (2).

101-0: Cervical vertebrae, epipophyses: poorly developed and rounded (0); well developed and finger-like (1).

113-0: Caudal vertebrae, ventral surface: flat (0); double keel (1); single keel (2).

155: Pubis, obturator opening: foramen (0); incipient notch (1); wide and well-developed opening (2) (1; Benson et al., 2010a: 172).

184-1: Femur, lateral condyle: does not project further distally than medial condyle (0); projects distinctly further than medial condyle and distal surface of medial condyle is gently flattened in comparison (1).

202-1: Pes, shafts of metatarsals: not appressed (0); appressed (1).

203-1: Metatarsal III, distal articular surface in anterior view: poorly extended dorsally and slightly excavated (0); well-developed ginglymoid, very extended proximally (1).

209-1: Metatarsal III, ratio of length to minimum width: short and stout, ratio smaller than 10 (0); slender, ratio larger than 10 (1).

228-1: Ulnar shaft: straight (0); caudally bowed (1).

290-1: Sacral centrums, with a constriction at mid-length: (0) absent; (1) present.

308-1: Metatarsal III, in anterior view: (0) subequal in length and distal width with other metatarsals, (1) longer and laterally wider distal end than the other metatarsals.

309-1: Margins of the shafts of metatarsals IV: (0) sinuous or concave in the entire length, (1) straight only curving in the distal end, or (2) straight in the entire length.

332-0: Lacrimal prominence: (0) absent; (1) triangular hornlet; (2) ridge continuous with raised surface of lateral edge of nasals.

**Character supporting Tyrannosauroidea + Megaraptora (both analyses):**

15-2: Premaxilla, number of teeth: 4 (0); 3 (1); 5 (2); more than 5 (3); absent (4)

86-1: Articular, retroarticular process: long, narrow and rod-shaped (0); wide, with a posterior groove (1)

135-1: Metacarpal III, length: >0.75 length of metacarpal II (0); <0.75 length of metacarpal II (1).

158-1: Pubis, pubic tubercle: absent (0); present as a convexity on the anterior margin of the pubis (1); present as a rugose flange that is discretely offset from the anterior margin of the pubis and is bordered posteriorly by heavy rugosities on the lateral surface on the obturator region of the pubis (2).

160-1,2: Pubis, length of pubic boot: ≤40% length of the bone (0); ≥40% length of the bone (1); ≥60% length of the bone (2).

245-1: Ilium, lateral surface of ilium with large external foramina and internal pneumatic spaces: absent (0); present (1).

279-1: Cervical vertebrae, anterior and middle cervical vertebra centra: anterior and posterior articular surfaces at the same horizontal level (0); posterior articular surface strongly ventrally offset from the anterior one (1).

335-1: Postorbital, sharp and long ridge that runs along the lateral margin of the bone: (0) absence; (1) presence.

340-2: Frontal length (taken only in dorsal view): (0) frontals shorter than parietals; (1) being equal or larger than the parietal length; (2) being almost two times the length of the parietals; (3) being almost four times the length of the parietals.

341-1: Frontal lateral expansion: (0) strong and posteriorly placed expansion; (1) moderately expansion extending from the posterior corner to the middle of the bone; (2) absent; (3) strong and posterior expansion extending unto the half of the bone.

**Character supporting Tyrannosauroidea (both analyses):**

13-1: Premaxilla, orientation of tooth row: strongly parasagitally (anteroposteriorly) (0); first two teeth oriented mediolaterally and third and fourth teeth oriented parasagitally (1); entire tooth row oriented mediolaterally and all teeth visible in anterior view (2).

27-2: Premaxilla, depth/length ratio under external nares: 0.5-1.25 (0); <0.5 (1); >1.25 (2)

51-1: Postorbital, anterior process: dorsally oriented (0); anteriorly oriented (1)

91-1: Mandibular, arcade contour: V-shaped (0); U-shaped (1)

95-1: Axis, distal extreme of neural spine: not expanded (0); expanded, forming a spine table (1) (1).

175-1: Femur, fossa on the medial surface of the head, laterals to the trochanteric fossa, form: absent or shallow (0); deep (1).

226-1: Ilium, anterior margin of preacetabular blade: straight or convex (0); with a rostrodorsal notch (1).

233-0: Humerus, length relative to the femur: 50-70% (0); 50-30% (1); 20-30% (2)

242-2: Vertical ridge on iliac blade above acetabulum: absent (0); developed as a low swollen ridge with associated foramina (1); present as a well-developed ridge (2).

274-1: Articular, mediolateral width of jaw muscle attachment site: less than (0) or equal to or greater than (1) width of glenoid for articulation with quadrate.

275-1: Articular, smooth non-articular region between glenoid and attachment site for depressor mandibular muscles: present (0); absent (1)

285-1: Premaxillary symphysis, ventral view: acute, V-shaped (0) or broad, U-shaped (1).

323-0: Manual ungual curvature: (0) moderate; (1) extremely curved; (2) straight.

**Character supporting Megaraptora (with fragmentary taxa):**

126-1: Humerus, form of distal condyles: lateral and medial condyles expanded equally (offset from shaft in anterior or posterior view is equal) (0); medial condyle expanded further medially than the lateral condyle is laterally (1)

141-1: Manual ungual I-2, transverse compression: absent; sub-rectangular to sub-triangular in proximal view (0); strongly transversely compressed, being oval with a distinct dorsoventral axis in proximal view (1).

151-1: Ilium, supraacetabular crest, maximum lateral projection relative to ischial peduncle: significantly greater (0); subequal (1).

179-2: Femur, anterior trochanter: distally positioned, under the ventral level of the femoral head (0); more proximally positioned, but distal to the greater trochanter (1); at the same level or proximal to the greater trochanter (2).

180-0: Femur, fourth trochanter: robust (0); reduced or absent (1).

181-1: Femur, extensor groove: absent (0); wide and shallow (1); narrow and deep (2).

185-1: Femur, mediodistal crest: absent or poorly developed (0); extended, representing more than a quarter of femoral length (1).

190-1: Tibia, medial expansion of distal medial malleolus: slightly expanded (0); expanded 40% or more than tibial mid-shaft width (1).

198-1: Astragalus, with a prominent posterolateral extension: absent (0); present (1).

204-1: Metatarsal III, extensor fossa of the distal end: superficial (0); deeply excavated with a very wide and crescent-shaped flexor fossa (1).

310-2: Second and third metatarsal contact: (0) by a concave-convex surface, (1) by a smooth surface, or (2) by a flat surface.

**Character supporting Megaraptora (without fragmentary taxa):**

141-1: Manual ungual I-2, transverse compression: absent; sub-rectangular to sub-triangular in proximal view (0); strongly transversely compressed, being oval with a distinct dorsoventral axis in proximal view (1).

198-1: Astragalus, with a prominent posterolateral extension: absent (0); present (1).

214-1: Sacrum, ventral surface: keeled (0); flattened, usually with a ventral sulcus (1)

**Character supporting Megaraptoridae (without fragmentary taxa):**

127-1: Ulna, shape of the olecranon process: anteroposteriorly short and square shaped in lateral view (0); anteroposteriorly long, being extended at the base of the coronoid process, and widely sub-triangular in lateral view (1).

128-1: Ulna, olecranon: wide, transversely expanded (0); transversely compressed (1).

129-1: Ulna, lateral tuberosity: absent or poorly developed (0); developed as a prominent crest, that is distally continuous with a longitudinal ridge formed at the intersection of the craniolateral and caudolateral surfaces of the ulnar shaft (1).

145-1: Manual unguals, ventral margin of manual unguals I and II: flat or slightly convex (0); a well-developed ridge that connects the flexor tubercle (1).

292-1: Fibula, length compared with femoral length: subequal (0); shorter (ca. 70%) (1).

322-2: Manual ungual length: (0) Short; (1) moderate; (2) extremely long; ORDERED.

325-1: Metatarsal III, area of the proximal surface: (0) similar in size to metatarsal II and IV; (1) clearly larger than metatarsals II and IV; (2) clearly smaller than metatarsal II and IV. ORDERED.

***Murusraptor +* (*Maiposaurus* + *Tratayenia* + *Aerosteon*)( with fragmentary taxa)**

200-0: Calcaneum, transverse development: moderately wide (0); strongly compressed, being a disc-like element (1).

201-0: Calcaneum, shape in lateral or medial view: roughly symmetric, with wide angles in the posterior border (0); strongly assymetric, with a right angles in the posterior border (1).

216-1: Manual unguals, proximodorsal lip: absent (0); present (1).

355-0: Tibia, dorsal curvature of the shaft: curved (0); shaft straight (1).

**South American megaraptorids (without fragmentary taxa):**

2-2: Teeth, denticles density: mesial and distal denticles subequal (0); more mesial denticles than distal denticles (mesial denticles considerably smaller) (1); mesial denticles absent (2).

292-2: Flexor tubercle of ungual phalanxs transversally divided by a lateromedial sulcus: (0) absent; present, but sulcus is not anteroposteriorly extended (1); present, sulcus forming a anteroposteriorly wide and smooth platform (2).

355-0: Tibia, shaft with dorsal curvature: curved (0), straight (1).

***Tratayenia + Maiposaurus + Aerosteon*** **(without fragmentary taxa)**

352-1: Dorsal vertebrae, bifurcated prezygodiapophyseal fossa forming an accessory fossa: (0) absent; (1) present.

353-0: Proximal caudal vertebrae, shape of pre- and postzygapophyseal articular facets: round (0) and elongated (1).

Supplementary information B

**Two different “megaraptoran forms” in Australia.**

The phylogenetic analysis supports that the South American forms are more closely related each other than with other species, even within contemporaneous species from different landmasses such as *Australovenator* and UNPSJB-PV-944/958. Furthermore, the fragmentary evidence shows us that the Australian taxa are more similar each other than with the species from South America. These fossils have not been included in the data matrix because of its fragmentary nature. Nevertheless, the megaraptoran records from Strzelecki group, Eumeralla formation and Griman Creek formation were taken as independent OTUs and extensively compared with those Australian records that forms part of our phylogenetic analysis. To chart our results, we put those three OTUs within the Fig. 18.


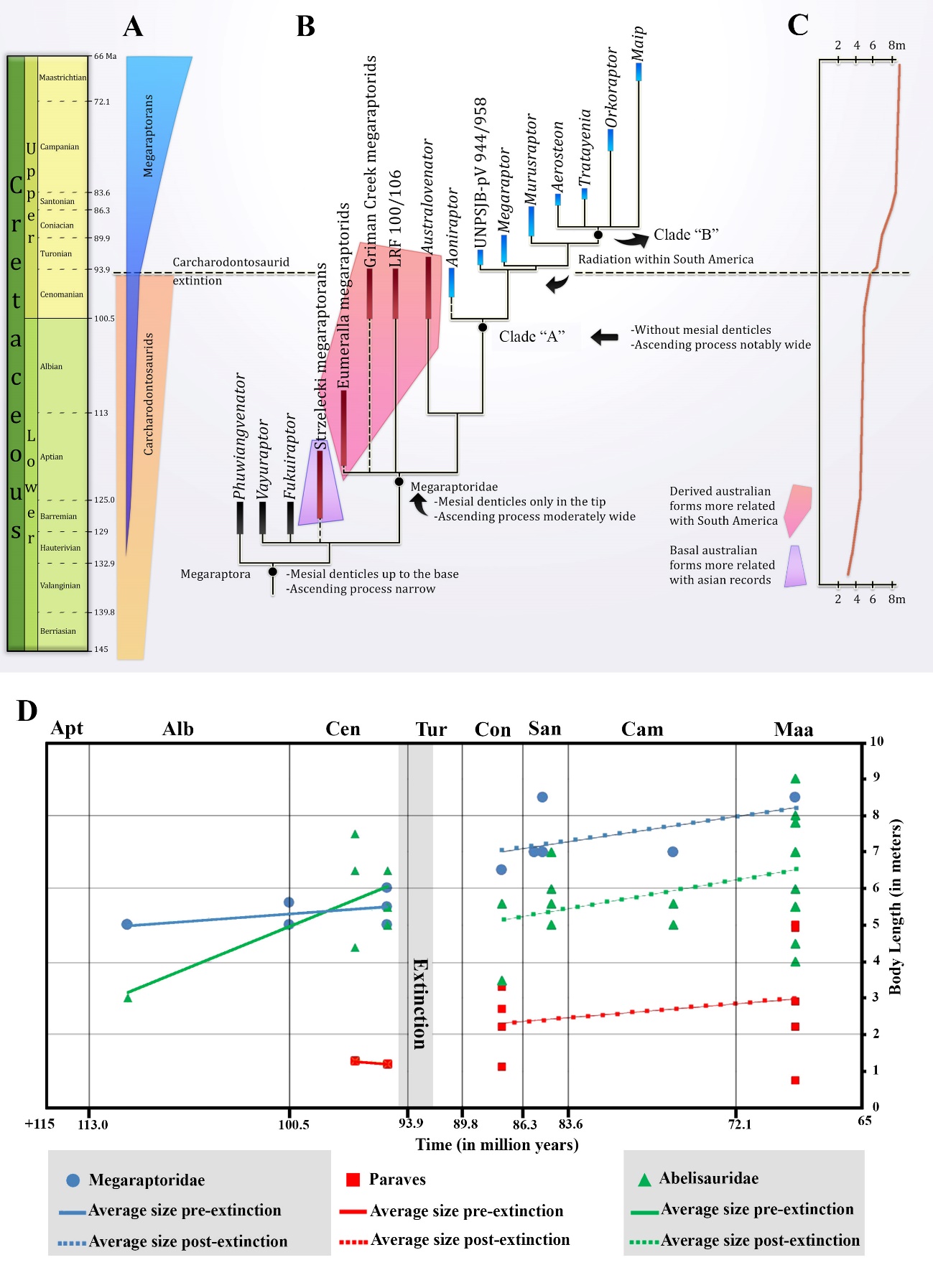


As commented in the paragraph “Other potential characters for the South American megaraptorids”, `*Rapator ornitholestoides´* and *Australovenator*^18-19^ shows more similarities between each other than with *Megaraptor* (Fig. 17 C). Moreover, the available ulnae of Australia (NMV P186076, LRF 100/106 and *Australovenator*)^19-20^ are more similar to each other than with the known ulna of *Megaraptor* (Fig. 17 D).

Furthermore, within Australia the older specimens (those of Strzelecki Group; Barremian-Aptian) shares features with basal megaraptorans such as *Fukuiraptor* or the Thai taxa, while younger records (such as those from the Albian through Lower Turonian Eumeralla, Winton, and Griman Creek formations) resembles more with more derived megaraptorids (Fig. 18 B). In this sense, the presence of tooth with mesial denticles until de base of the crown is a feature observed in *Fukuiraptor* (Fig. 16 A) but also the isolated tooth from Strzelecki Group (MV P186353, MV P210084, MV P212859 and MV P230871). In contrast, teeth of *Australovenator* (Cenomanian–?Turonian) show mesial denticles on the apex of the crown (Fig. 16 A´). Also, the teeth of the Megaraptoridae cf. *Australovenator wintonensis* (NMV P252264 and NMV P239459) from Eumeralla Formation (Albian) shows a wear facet on its tip not allowing the preservation of mesial denticles but the rest of the crown is well-preserved and devoid of such structures, similarly to *Australovenator*. In the derived South American forms (Coniacian-Maastrichtian), the teeth loss the mesial denticles (Fig. 16 A´´), as it is observed in *Megaraptor, Orkoraptor, Murusraptor* and the megaraptorid tooth from Chorrillo Formation (MACN-Pv 19066). Other feature is the presence, on the astragalus, of a narrower ascending process in Barremian-Aptian forms (*Vayuraptor, Phuwiangvenator* and the isolated astragalus from Strzelecki Group [NMV P150070]; Fig. 16 E); which represents approximately 60% of the transversal width of the astragalar body. On the other side, in the Albian-to- Turonian forms (such as *Australovenator* and the Megaraptoridae cf. *Australovenator wintonensis* [NMV P253701]; Fig. 16 E´) the ascending process is slightly wider and represents approximately a 65% of the astragalar body. This feature is more developed on the Santonian megaraptorid *Aerosteon* which exhibits an ascending process being a 75% of the width of the astragalar body (Fig. 16 E´´).

In sum, within Australia there is different “megaraptoran forms”. The older ones (those of Strzelecki group) represents basal megaraptorans forms more related with contemporaneous forms from Japan and Thailand while the younger forms (those of Eumeralla, Winton, and Griman Creek formations) represents derived megaraptorids forms more related with the South American forms.

References.

1. Grillo, O. N., & Delcourt, R. Allometry and body length of abelisauroid theropods: *Pycnonemosaurus nevesi* is the new king. *Cret Res*. **69**, 71-89. (2017).
2. Porfiri, J. D., Novas, F. E., Calvo, J. O., Agnolín, F. L., Ezcurra, M. D, & Cerda, I.A. Juvenile specimen of *Megaraptor* (Dinosauria, Theropoda) sheds light about tyrannosauroid radiation. *Cret Res*. **51**:35-55. (2014)
3. Novas, F. E. *Megaraptor namunhuaiquii* gen. et. sp. nov., a large-clawed, Late Cretaceous Theropod from Argentina. *J. Vert. Pal.* **18**, 4-9 (1998).
4. Lamanna, M. C., Casal, G. A., Martínez, R. D., & Ibiricu, L. M. Megaraptorid (Theropoda: Tetanurae) Partial Skeletons from the Upper Cretaceous Bajo Barreal Formation of Central Patagonia, Argentina: Implications for the Evolution of Large Body Size in Gondwanan Megaraptorans. *An of Carn Mus*. **86**(3), 255-294. (2020).
5. Sereno, P. C., Martínez, R. N., Wilson, J.A., Varricchio, D.J., & Alcober, O.A. Evidence for avian intrathoracic air sacs in a new predatory dinosaur from Argentina. *PLoS One* **3**, e3303 (2008).
6. Coria, R. A., & Currie, P. J. A new megaraptoran dinosaur (Dinosauria, Theropoda, Megaraptoridae) from the Late Cretaceous of Patagonia. *PLoS One*. 11:e0157973. (2016)
7. Lambe, L. M. (1917). The Cretaceous theropodus dinosaur *Gorgosaurus* (No. 83). Ottawa, Government printing bureau.
8. Aranciaga-Rolando, M. A., & Novas, F. E. A reanalysis of *Murusraptor barrosaensis* Coria & Currie (2016) affords new evidence about the phylogenetical relationships of Megaraptora. *Cret Res*. **99**:104-127. (2019)
9. Novas, F. E., Ezcurra, M. D., Agnolin, F. L., Pol, D., & Ortiz, R. New Patagonian Cretaceous theropod sheds light about the early radiation of Coelurosauria. *Rev Mus Argentino Cienc Nat*. **14**:57-81. (2013)
10. Porfiri, J. D., Juárez-Valieri, R. D., Santos, D. D. D. A new megaraptoran theropod dinosaur from the Upper Cretaceous Bajo de la Carpa Formation of northwestern Patagonia. *Cret. Res*. doi: 10.1016/j.cretres.2018.03.014. (2018)
11. Apesteguía, S., Smith, N. D., Juárez-Valieri, & R., Makovicky, P. J. An Unusual New Theropod with a Didactyl Manus from the Upper Cretaceous of Patagonia, Argentina. *PLoS ONE*. **11**(7): e0157793. (2016)
12. Samathi, A., Chanthasit, P., & Sander, P. M. Two new basal coelurosaurian theropod dinosaurs from the Lower Cretaceous Sao Khua Formation of Thailand. *Acta Palaeontol. Pol*. **64**(2):239-260. (2019)
13. Motta, M. J., Aranciaga-Rolando, A. M., Rozadilla, S., Agnolín, F. E., Chimento, N. R., Brissón-Egli, F. Novas, F. E. New theropod fauna from the Upper Cretaceous (Huincul Formation) of northwestern Patagonia, Argentina. *New Mex Mus Nat Hist Sci Bull*. **7**:231-253. (2016)
14. Goloboff, P. A., & Catalano, S. A. TNT version 1.5, including a full implementation of phylogenetic morphometrics. *Cladistics*, **32**(3), 221-238. (2016)
15. Goloboff, P. A., Farris, J. S., & Nixon, K. C. TNT, a free program for phylogenetic analysis. *Cladistics*, **24**(5), 774-786. (2008).
16. Aranciaga-Rolando, A. M. A., Brisson-Egli, F., Sales, M. A., Martinelli, A. G., Canale, J. I., & Ezcurra, M. D. A supposed Gondwanan oviraptorosaur from the Albian of Brazil represents the oldest South American megaraptoran. *Cret Res*. **84**:107-119. (2018)
17. White, M. A., Bell, P. R., Cook, A. G., Poropat, S. F., & Elliott, D. A. The dentary of *Australovenator wintonensis* (Theropoda, Megaraptoridae); implications for megaraptorid dentition. *PeerJ*, **3**, e1512. (2015)
18. White, M. A., Cook, A. G., Hocknull, S. A., Sloan, T., Sinapius, G. H., & Elliott, D. A. New forearm elements discovered of holotype specimen *Australovenator wintonensis* from Winton, Queensland, Australia. *PloS one*. **7**(6):e39364. (2012)
19. White, M. A., Falkingham, P. L., Cook, A. G., Hocknull, S. A., & Elliott, D. A. Morphological comparisons of metacarpal I for *Australovenator wintonensis* and *Rapator ornitholestoides*: implications for their taxonomic relationships. *Alcheringa*. **37**:1-7. (2013)
20. Bell, P. R., Cau, A., Fanti, F., & Smith, E. A large-clawed theropod (Dinosauria: Tetanurae) from the Lower Cretaceous of Australia and the Gondwanan origin of megaraptorid theropods. *Gond Res.* doi:10.1016/j.gr.2015.08.004. (2015)
